# Transcriptional coupling of telomeric retrotransposons with the cell cycle

**DOI:** 10.1126/sciadv.adr2299

**Published:** 2025-01-03

**Authors:** Mengmeng Liu, Xiao-Jun Xie, Xiao Li, Xingjie Ren, Jasmine L. Sun, Zhen Lin, Rajitha-Udakara-Sampath Hemba-Waduge, Jun-Yuan Ji

**Affiliations:** ^1^ Department of Biochemistry and Molecular Biology, Tulane University School of Medicine, Louisiana Cancer Research Center, 1700 Tulane Avenue, New Orleans, LA 70112, USA.; ^2^ Department of Molecular and Cellular Medicine, College of Medicine, Texas A&M University Health Science Center, College Station, TX 77843, USA.; ^3^ Institute for Human Genetics and Department of Neurology, University of California, San Francisco, San Francisco, CA 94143, USA.; ^4^ Department of Pathology, Tulane University School of Medicine, Louisiana Cancer Research Center, 1700 Tulane Avenue, New Orleans, LA 70112, USA.

## Abstract

Unlike most species that use telomerase for telomere maintenance, many dipterans, including *Drosophila*, rely on three telomere-specific retrotransposons (TRs)—*HeT-A*, *TART*, and *TAHRE*—to form tandem repeats at chromosome ends. Although TR transcription is crucial in their life cycle, its regulation remains poorly understood. This study identifies the Mediator complex, E2F1-Dp, and Scalloped/dTEAD as key regulators of TR transcription. Reducing the activity of the Mediator or Sd/dTEAD increases TR expression and telomere length, while overexpressing E2F1-Dp or depleting Rbf1 stimulates TR transcription. The Mediator and Sd/dTEAD regulate this process through E2F1-Dp. CUT&RUN (Cleavage under targets and release using nuclease) analysis shows direct binding of CDK8, Dp, and Sd/dTEAD to telomeric repeats, with motif enrichment revealing E2F- and TEAD-binding sites. These findings uncover the Mediator complex’s role in controlling TR transcription and telomere length through E2F1-Dp and Sd, coupling the transcriptional regulation of the TR life cycle with host cell-cycle machinery to protect chromosome ends in *Drosophila*.

## Teaser

The life cycle of telomeric retrotransposons is transcriptionally linked to host cell-cycle machinery via E2F1 in *Drosophila*.

## INTRODUCTION

Telomeres protect chromosome ends and play critical roles in chromosome replication and genome stability in species with linear chromosomes. While telomerase-based telomere maintenance is common among most eukaryotes, many dipteran insects, including *Drosophila melanogaster*, lack the telomerase enzyme and the short telomeric DNA repeats it generates. Instead, they have evolved an exceptional strategy for telomere elongation (*1*–*3*). Exploring these alternative telomere maintenance strategies has contributed to a deeper understanding of the diversity, complexity, and richness of life on Earth.

In *Drosophila*, telomere elongation is accomplished through the transposition of telomere-specific retrotransposons (TRs), which are directed to chromosome ends and serve a critical role in maintaining chromosome stability (*1*–*4*). Decades of genetic and cytological research have identified three TRs involved in this process: *HeT-A* (*healing transposon*) (*5*, *6*), *TART* (*telomere-associated retrotransposon*) (*7*), and *TAHRE* (*telomere-associated and HeT-A–related element*) (*8*). Randomly transposed copies of these three TRs form head-to-tail arrays, which constitute the telomeres in *Drosophila* (*1*–*4*). *TART* and *TAHRE* contain two open reading frames (ORFs): ORF1 encodes Gag proteins, while ORF2 encodes a Pol protein with reverse transcriptase and endonuclease activities (*8*, *9*). In contrast, *HeT-A* lacks ORF2 and only encodes a Gag-like protein (*10*). Following transcription by host-cell RNA polymerase II (Pol II), the sense-strand transcripts of *HeT-A* and *TART* are translated in cytoplasm to produce Gag proteins. These Gag proteins then associate with the sense-strand RNAs from all three TRs, transporting them back into the nucleus and to the chromosome ends, where they serve as templates for reverse transcription and incorporation at telomeres (*1*–*4*). The *HeT-A* Gag protein oligomerizes with the Gag proteins of *TART* and *TAHRE*, thereby facilitating the telomere-targeting function of all three elements (*11*–*13*).

Translation and retrotransposition are constitutive processes, making the transcriptional regulation of TRs, the initial step in their transposition life cycle, a key factor in maintaining telomere homeostasis. While both sense and antisense strands of *TART* elements are transcribed, *HeT-A* and *TAHRE* elements are primarily expressed in the sense orientation (*3*). In *Drosophila* ovaries, the expression of TRs, particularly that of *HeT-A* and *TAHRE*, is repressed by Piwi-interacting RNA (piRNA)–medicated silencing (*14*–*17*). However, this silencing does not occur in somatic cells (*18*). In addition, several transcriptional factors and cofactors have been implicated in the regulation of TR expression. For instance, depleting Trf2 (TATA box–binding protein–related factor 2) and Woc (without children) in ovaries results in increased TR expression (*19*). Components of the insulator complex, such as BEAF32, Chromator (Chro, also known as Chriz), and DREF, have been shown to directly repress *TART* expression, but not *HeT-A*, during oogenesis (*20*). Furthermore, several chromosomal proteins, including Chro, Z4 (encoded by *putzig*, or *pzg*), and the TRF2/DREF complex, are known to bind to telomeric repeats (*21*). *HeT-A* transcription is decreased in *JIL-1* kinase mutant larvae but increased in *Z4*/*pzg* mutant larvae (*22*). Despite these findings, the mechanism controlling TR transcription, especially in somatic cells, is still not fully understood.

Transposable elements, including both transposons and retrotransposons, along with parasitic viruses like DNA viruses, RNA viruses, and retrovirus, are the most common forms of parasitic nucleic acids that target eukaryotes (*23*). Similar to retroviruses, retrotransposons (class I transposable elements) depend on host-cell Pol II–dependent transcription for a crucial part of their life cycle (*23*). The activation of non-TR retrotransposons has been associated with various health-related issues, including sterility, cancer, aging, and other diseases, indicating its detrimental impact (*24*). Deciphering the regulatory mechanisms controlling the transcription of TRs is key to uncovering how dipteran cells have adapted these cellular parasites to maintain genome stability.

In eukaryotes, the Mediator complex functions as the major coactivator for the transcription of both protein-coding and most noncoding RNA genes (*25*, *26*). This complex, comprising around 30 unique and conserved subunits, is organized into four modules: head, middle, tail, and CDK8 kinase module (*25*, *26*). The head, middle, and tail modules can be purified together as the small or core Mediator complex, which can reversibly interact with the CDK8 kinase module to form the large Mediator complex (*27*). This modular arrangement provides the Mediator complex with a large, flexible surface area that facilitates interactions with a variety of transcriptional activators and cofactors in diverse developmental and physiological contexts (*25*, *26*). Recent cryogenic electron microscopy studies have shown that the head and middle modules of the Mediator complex directly interact with the Pol II C-terminal domain, creating a molecular bridge between Pol II and enhancer-bound transcription factors (*28*–*30*).

Phylogenetic studies of Mediator subunits across the eukaryotic kingdom have revealed a primitive core Mediator proto-complex, composed of 17 highly conserved subunits, which likely existed in the protoeukaryote approximately 1 to 2 billion years ago (*31*). It has been hypothesized that the Mediator complex originally evolved as a defense mechanism against harmful transposable elements and retroviruses in protoeukaryotes, possibly by inhibiting the activities of activators used by parasitic transposons and retroviruses (*32*). This evolutionary arms race may have driven the complexity of Pol II–dependent transcription and the Mediator complex in eukaryotes (*32*). Despite these intriguing insights, it remains unclear whether and how the Mediator complex regulates the transcription of TRs and other retrotransposons in *Drosophila*.

In this study, we analyze the transcriptional regulation of *Drosophila* TRs and demonstrate its role in maintaining cell-cycle-dependent telomere homeostasis. We identified multiple subunits of the Mediator complex and two transcriptional factors, E2F1-Dp and Scalloped (Sd/dTEAD), as key regulators of TR transcription and telomere length in *Drosophila*. The E2F1-Dp dimer is an essential transcription factor that regulates DNA replication in polyploid cells undergoing endocycling and controls the G_1_ to S phase transition in the mitotic cell cycle (*33*–*35*). Our mutational analyses suggest that the large Mediator complex represses TR transcription through E2F1-Dp and Sd/dTEAD, while the small Mediator complex is necessary for E2F1-Dp–dependent TR transcription. Furthermore, CUT&RUN (cleavage under targets and release using nuclease) analyses reveal the direct binding of CDK8, Dp, and Sd/dTEAD to telomeric repeats. These findings identify the three TRs as direct transcriptional targets of E2F1-Dp, similar to other E2F1-Dp targets involved in DNA replication during the S phase. Together, our results illustrate the robust coupling between TR transcription and the host cell-cycle machinery, which together regulate telomere dynamics to support the TR life cycle and maintain genomic stability in *Drosophila*.

## RESULTS

### Increased expression of TRs and longer telomeres in *Cdk8* and *CycC* mutants

**Fig. 1.**
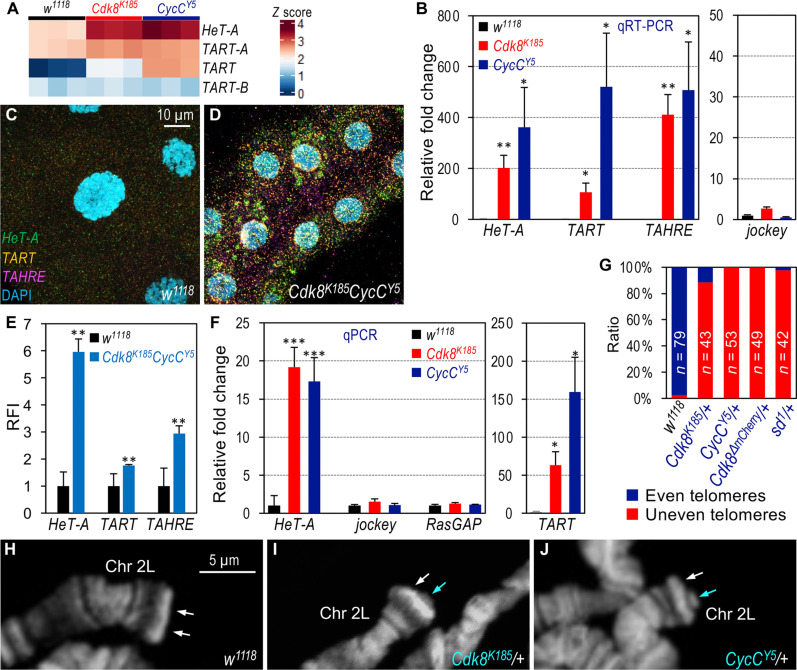
Elevated expression of TRs and increased telomere length in *Cdk8* and *CycC* mutant larvae. (**A**) Heatmap showing the expression levels of *HeT-A* and *TART* in triplicate samples from *w^1118^* (control), *Cdk8^K185^*, and *CycC^Y5^* mutant larvae at the third instar wandering stage. (**B**) qRT-PCR analysis of *HeT-A*, *TART*, and *TAHRE* expression in *Cdk8^K185^* (red) and *CycC^Y5^* (dark blue) mutant larvae, with *w^1118^* (black) as the control. The expression of the non–LTR-retrotransposon *jockey* (*jockey gag*) serves as a negative control. (**C** and **D**) Detection of *HeT-A* (green), *TART* (orange), and *TAHRE* (magenta) mRNA transcripts using HCR RNA-FISH in the salivary glands of control [(C) *w^1118^*] and *Cdk8 CycC* double mutants [(D) genotype: “+; +; *Cdk8^K185^ CycC^Y5^*”]. Nuclei are stained with 4′,6-diamidino-2-phenylindole (DAPI, blue). Scale bar, (C) 10 μm. (**E**) Quantification of the relative fluorescence intensity (RFI) of the three TRs in *Cdk8^K185^ CycC^Y5^* double mutants, normalized to the control (*w^1118^*). (**F**) Telomere length measurement by qPCR analysis of *HeT-A* and *TART* using genomic DNA from *Cdk8^K185^* and *CycC^Y5^* mutant larval brains. *RasGAP* (neighboring gene of *Cdk8*) and *jockey* serve as specificity and negative controls, respectively. The *TAHRE* level was undetectable in this experiment. Statistical significance: *P < 0.05, **P < 0.01, ***P < 0.001 (one-tailed unpaired *t* tests). (**G**) Bar chart showing the frequency of even versus uneven telomeres in the indicated genotypes. The total number of karyotypes analyzed for each genotype is noted within the bars. (**H** to **J**) Visualization of longer telomeres in polytene chromosomes from *Cdk8^K185^/+* (I) and *CycC^Y5^/+* (J) heterozygous larvae. Uneven chromosomal ends, indicated by arrows, are compared to the control [(H) *w^1118^*]. Scale bar, 5 μm.

Homozygous null mutants of *Cdk8* (*Cdk8^K185^*) or *CycC* (*CycC^Y5^*) are lethal but can survive to the pupal stage due to maternal contributions of CDK8 and CycC (*36*, *37*). To investigate the defects in *Cdk8* and *CycC* mutants, we analyzed global gene expression profiles in late third-instar larvae using RNA sequencing (RNA-seq), followed by “Pathway” and “Gene Ontology” cluster analyses (*38*). Consistent with previous reports, we confirmed that mutations in *Cdk8* and *CycC* affect the transcription of genes involved in lipogenesis (via sterol regulatory element–binding protein) (*39*, *40*), metamorphosis (via ecdysone receptor) (*37*), and DNA replication (via E2F1) (*41*, *42*). Unexpectedly, we also observed a notable up-regulation of retrotransposons such as *HeT-A* and *TART* (Fig. 1A and fig. S1, A and D; reduced expression of *Cdk8* and *CycC* is shown in fig. S1E). *HeT-A* and *TART*, along with *TAHRE*, are unique TRs crucial for maintaining telomere length in many dipteran insects (*2*, *3*, *43*, *44*). Of these, *HeT-A* is the most abundant, followed by *TART*, while *TAHRE* is relatively rare at telomeres (*8*, *45*). We verified the up-regulation of these TR transcripts in both *Cdk8* and *CycC* mutants through quantitative reverse transcription polymerase chain reaction (qRT-PCR), with the non-LTR (long terminal repeat) retrotransposon *jockey* (*jockey gag*) and the non-retrotransposon RNA Pol II–transcribed *RasGAP* gene serving as controls (Fig. 1B).

To further validate the effect of *Cdk8* and *CycC* mutation on TR expression, we used hybridization chain reaction RNA fluorescence in situ hybridization (HCR RNA-FISH). This sensitive technique allows the multiplexed and quantitative detection of mRNA transcripts in individual cells (*46*). The large polyploid cells in larval salivary glands enable the visualization of individual mRNA transcripts at the cellular level (*40*). As shown in Fig. 1C and fig. S1F, the three TRs are normally expressed at low levels. However, their expression is markedly increased in *Cdk8^K185^ CycC^Y5^* double-mutant larvae (Fig. 1D and fig. S1G; quantified in Fig. 1E). These observations, consistent with both RNA-seq and qRT-PCR analyses, suggest a key role for CDK8 and CycC in negatively regulating TR expression.

To assess the effect of up-regulated TR expression on telomere length in *Cdk8* and *CycC* mutants, we used qPCR to measure the number of TRs in telomeres, an established method to quantify TR repeats (*47*, *48*). We focused on diploid larval brains to avoid the varying ploidy levels present in other larval tissues. The copy numbers of *HeT-A* and *TART* were substantially increased (Fig. 1F; *TAHRE* was too low to be detected in this experiment). We confirmed that this increase corresponded to longer telomeres by directly visualizing them on polytene chromosomes. To compare the effect of mutations, *Cdk8^K185^* and *CycC^Y5^* mutants were independently crossed to wild-type flies to create heterozygotes, juxtaposing altered mutant telomeres with wild-type telomeres (*21*, *49*). Compared to the control (Fig. 1H), uneven polytene chromosome ends were observed in *Cdk8^K185^/+* (Fig. 1I) and *CycC^Y5^/+* (Fig. 1J) larvae. These effects were quantified across more than 40 sets of karyotypes for each genotype (Fig. 1G), indicating that loss of *Cdk8* and *CycC* has a dominant effect on telomere length. Notably, both *Cdk8^K185^* and *CycC^Y5^* alleles were created before 2007 (*36*), indicating that telomeres in these mutants have progressively elongated over time.

The three TRs (*HeT-A*, *TART*, and *TAHRE*) form terminal repeats, known as HTT arrays, at telomeres. Proximal to each HTT array are unique telomere-associated sequences (TAS), consisting of several kilobases of satellite DNA (*2*, *43*). Reporter genes inserted near or within TAS are typically transcriptionally silenced, a phenomenon known as the telomeric position effect (TPE). TPE has been instrumental in studying heterochromatin-induced gene silencing and telomere dynamics (*43*). Given the effects of *Cdk8* and *CycC* mutations on telomere length, we hypothesized that mutations in these genes would dominantly modify TPE. To test this, we crossed *Cdk8* and *CycC* mutant females with males carrying the fourth chromosome-linked TAS-inserted *118E-15* element, which places the *hsp70-white^+^* transgene under TPE (fig. S1H) (*50*). Multiple mutant alleles, including *Cdk8^K185^* (fig. S1I), *CycC^Y5^* (fig. S1J), and * Cdk8^K185^ CycC^Y5^* double mutants (fig. S1K), strongly enhanced the TPE phenotype in males (fig. S1H). This effect was quantified by measuring optical density at 485 nm (fig. S1L). Collectively, these findings support a crucial role for CDK8 and CycC in maintaining telomere homeostasis in *Drosophila*.

### Validation of CDK8-CycC’s role in regulating TR expression and telomere length

**Fig. 2.**
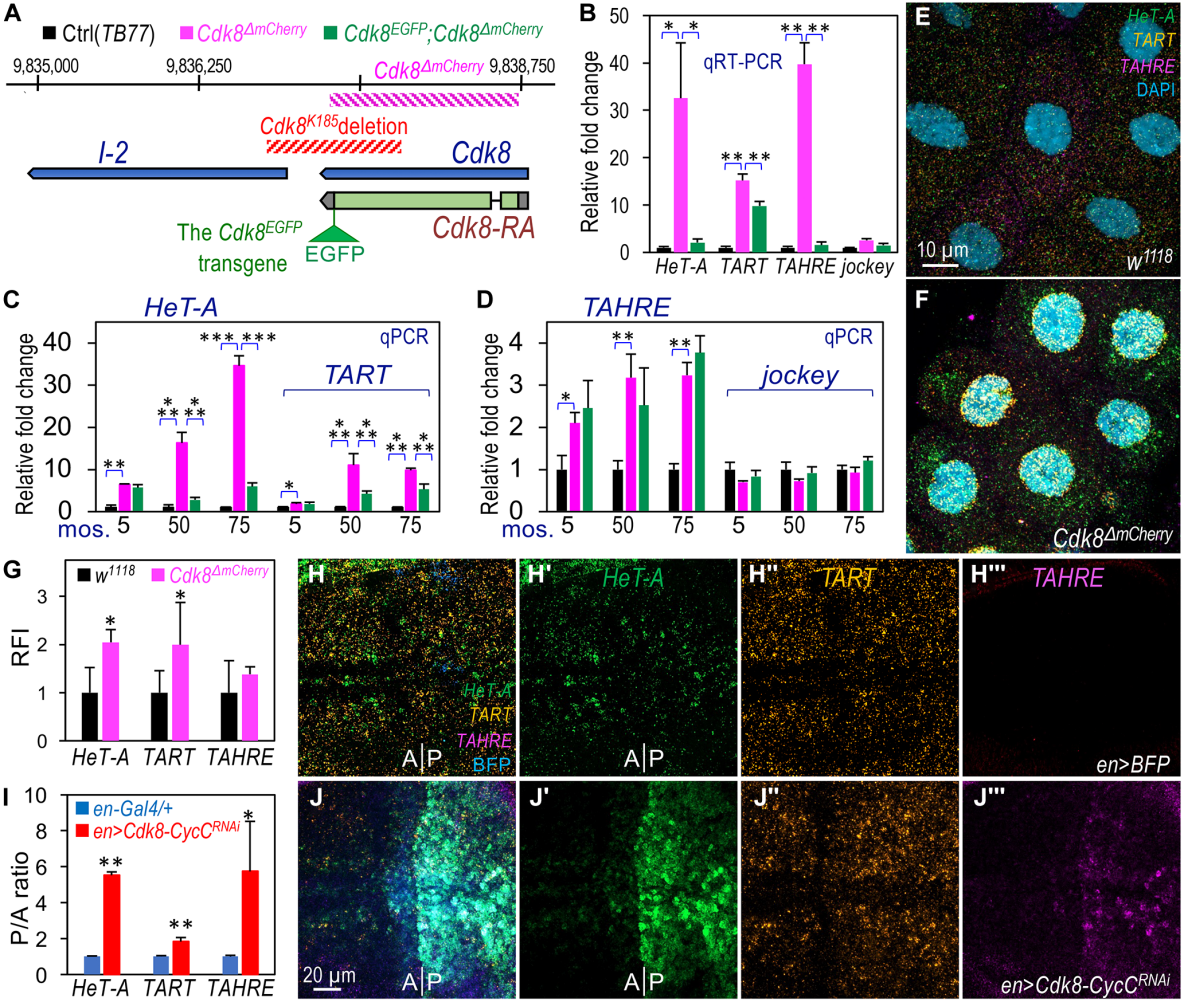
Validating the role of CDK8-CycC in regulating TR expression and telomere length. (**A**) Diagram of the genomic region of the *Cdk8* locus, highlighting the deleted regions (hatched lines). An EGFP tag is attached to the C terminus of the CDK8 protein (CDK8^EGFP^), with the rescue construct integrated into the second chromosome at the *attP40* site. (**B**) qRT-PCR analysis of mRNA levels of *HeT-A*, *TART*, and *TAHRE* in *Cdk8*^Δ*mCherry*^ (magenta), *Cdk8^EGFP^*; *Cdk8*^Δ*mCherry*^ (green), and *TB77* (control, black) larvae. (**C** and **D**) Telomere length measured by qPCR analysis using genomic DNA from *Cdk8*^Δ*mCherry*^, *Cdk8^EGFP^*; *Cdk8*^Δ*mCherry*^, or *TB77* (control) larvae. Note that the *Cdk8*^Δ*mCherry*^ allele was created in 2016, and the rescued animals were obtained in 2017. qPCR analyses using genomic DNA were conducted three times, in 2017, 2022, and 2024. (**E** to **G**) Detection of TR transcripts in salivary gland cells of control (E: *w^1118^*) and *Cdk8*^Δ*mCherry*^ homozygous mutant larvae (F) using the HCR RNA-FISH assay. Scale bar, (E) 10 μm. (G) Quantification of the RFI of the three TRs in *Cdk8*^Δ*mCherry*^ larvae. (**H** and **J**) Detection of mRNA transcripts of *HeT-A* (green), *TART* (orange), and *TAHRE* (magenta) in the wing disc of controls “*en-Gal4/+*; *UAS-BFP/+*” (H) and “*en-Gal4/+*; *UAS-Cdk8^RNAi^ CycC^RNAi^/UAS-BFP*” (J) using the HCR RNA-FISH assay. The posterior compartment is marked by the expression of BFP (blue fluorescent protein). A closer view of Fig. 2H is shown in fig. S2G. Scale bar, (J) 20 μm. (**I**) Ratio of fluorescence intensity in the posterior compartment and anterior compartment (P/A ratio; see fig. S2G for measurement) in the same wing discs of the indicated genotypes, with three to five discs analyzed. Specific genotypes are color-coded. Statistical significance: *P < 0.05, **P < 0.01, ***P < 0.001 (one-tailed unpaired *t* tests).

The original *Cdk8^K185^* null allele deletes part of *I-2* in addition to *Cdk8* (Fig. 2A) (*36*, *37*), which prompted us to generate a new *Cdk8* null allele using the CRISPR-Cas9 gene-editing system. We replaced the coding region of *Cdk8* with the *mCherry* gene, creating the *Cdk8*^Δ*mCherry*^ allele (Fig. 2A). Homozygous *Cdk8*^Δ*mCherry*^ mutants were pupal lethal. We confirmed the mutation by sequencing and verified the absence of detectable CDK8 protein (fig. S2A). The *Cdk8*^Δ*mCherry*^ mutants were rescued to viability and fertility by a transgenic genomic fragment of the *Cdk8* locus tagged with enhanced green fluorescent protein (EGFP) at the C terminus of CDK8 (*Cdk8^EGFP^*) (*37*).

We then tested whether the expression of TRs was altered in the *Cdk8*^Δ*mCherry*^ mutant larvae. As shown in Fig. 2B, the expression of TR transcripts was higher in *Cdk8*^Δ*mCherry*^ mutant larvae compared to the rescuing *w^1118^*; *Cdk8^EGFP^*; *Cdk8*^Δ*mCherry*^ and *TB77* control larvae. The *TB77* line [genotype: “*y,sc,v*”; (*51*)] was used for generating the *Cdk8*^Δ*mCherry*^ allele. In contrast, little difference was observed in the levels of the non-TR *jockey* (Fig. 2B). The longer telomeres in *Cdk8*^Δ*mCherry*^ mutants were confirmed by direct visualization in polytene chromosomes from *Cdk8*^Δ*mCherry*^/+ heterozygotes (fig. S2C cf. the control shown in fig. S2B; quantified in Fig. 1G). Consistent with these findings, the *Cdk8*^Δ*mCherry*^ allele also enhanced the TPEs (fig. S2D). These observations suggest a notable increase in telomere length in *Cdk8*^Δ*mCherry*^ mutants compared to controls.

To further validate this observation, we performed qPCR using genomic DNA samples. Over a 5-year period, we observed a continuous increase in *HeT-A* and *TART* copy numbers, but not in the rescued animals (genotype: *w^1118^*; *Cdk8^EGFP^*; *Cdk8*^Δ*mCherry*^) (Fig. 2C). The effect on *TAHRE* was marginal, likely because of very low copy numbers of the *TAHRE* element in telomeres (Fig. 2D). A statistically significant difference was noted between the telomere lengths of *TB77* and “*w^1118^*; *Cdk8^EGFP^*; *Cdk8*^Δ*mCherry*^,” possibly indicating a gradual shortening in mutant fly strains before the rescuing transgene was introduced over this 5-year period. By excluding potential contributions from unknown mutations in the genetic background, this genetic rescue provides critical evidence supporting the causal relationship between CDK8 loss, increased TR transcription, and telomere elongation. Using the HCR RNA-FISH assay, we also observed a notable increase in *HeT-A* and *TART* expression in polyploid salivary gland cells from *Cdk8*^Δ*mCherry*^ mutant larvae (Fig. 2F and fig. S2F) compared to the control (Fig. 2E and fig. S2E; quantified in Fig. 2G). *TAHRE* transcripts were scarcely detectable using this method.

Endogenous *HeT-A* is primarily expressed in actively proliferating diploid tissues, such as imaginal discs and larval neuroblasts (*52*–*54*). To test the effect of *Cdk8* and *CycC* mutations on TR expression in proliferating diploid tissues, we depleted CDK8 and CycC in wing discs and assessed TR expression using the HCR RNA-FISH assay. Low levels of *HeT-A* and *TART* were detected in the wing pouch region, while *TAHRE* transcripts were barely detectable (Fig. 2H and fig. S2G). TR expression was lower in the anterior nonproliferating cell zone (*55*) compared to the surrounding dividing cells (Fig. 2H and fig. S2G), an observation that will be explored further below. Upon depletion of CDK8 and CycC in the posterior compartment of wing discs using *en-Gal4* driven RNA interference (RNAi), the expression of all three TRs markedly increased in the cells of the posterior compartment compared to the anterior compartment (Fig. 2J; quantified in Fig. 2I; refer to fig. S2G for quantification). Similar observations were obtained with the depletion of either *Cdk8* or *CycC* alone using *en-Gal4* (fig. S3B, S3B′, and S3B″ and fig. S3C, S3C′, and S3C″). In contrast, these genetic manipulations had little effect on *jockey* expression (fig. S3A‴ to S3C‴). These findings further support the inhibitory role of CDK8-CycC in regulating TR expression and telomere length.

### Elevated TR expression and longer telomeres in other Mediator subunit mutants

**Fig. 3.**
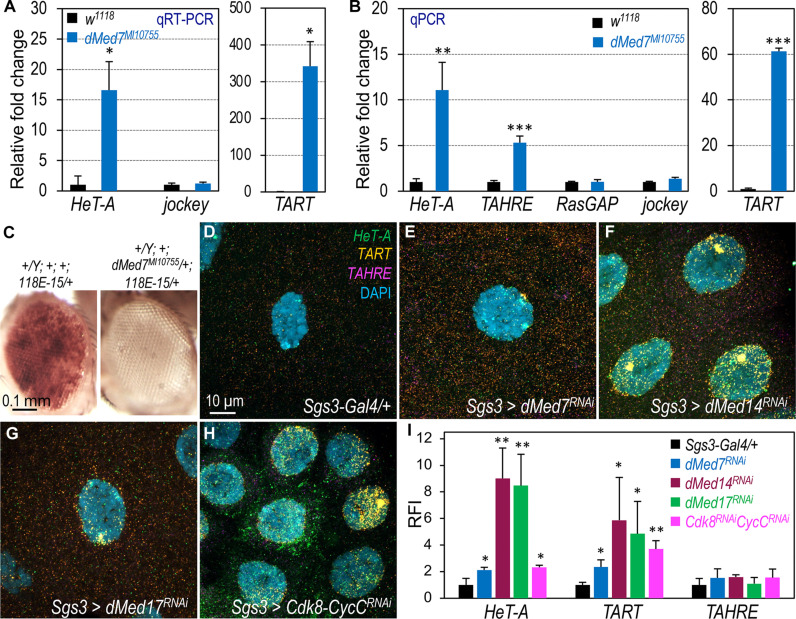
Disruption of additional Mediator subunits leads to increased TR expression. (**A**) qRT-PCR analysis of *HeT-A* and *TART* expression in *dMed7^MI10755^* mutant larvae at the third instar wandering stage. The expression of *TAHRE* was too low to be detected in this experiment. (**B**) Telomere length measurement by qPCR analysis of TRs using genomic DNA from *dMed7^MI10755^* mutant larvae. (**C**) Dominant enhancement of telomeric position variegation by the *dMed7^MI10755^* allele. (**D** to **H**) Detection of *HeT-A* (green), *TART* (orange), and *TAHRE* (magenta) mRNA transcripts using HCR RNA-FISH in the salivary glands of the indicated genotypes. Nuclei are stained with DAPI (blue). Genotype details: (D) *Sgs3-Gal4/+*; *+* (control); (E) *Sgs3-Gal4/+*; *UAS-dMed7^RNAi^/+*; (F) *Sgs3-Gal4/+*; *UAS-dMed14^RNAi^/+*; (G) and *Sgs3-Gal4/+*; *UAS-dMed17^RNAi^/+*; and (H) *Sgs3-Gal4/+*; *UAS-Cdk8^RNAi^ CycC^RNAi^/+.* Scale bar, (D), 10 μm. (**I**) Quantification of the RFI of the samples shown in (D) to (H). Specific genotypes are color-coded. Statistical significance: *P < 0.05, **P < 0.01, ***P < 0.001 (one-tailed unpaired *t* tests).

The effects of CDK8 and CycC, two conserved subunits of the Mediator complex, on TR expression and telomere length led us to investigate whether mutations in other Mediator subunits might similarly affect TR expression and telomere length. Classic mutant alleles for most Mediator subunits are either unavailable or cause embryonic lethality, such as *dMed12* (*kohtalo*) and *dMed13* (*skuld*) (*36*, *56*, *57*). Our search for Mediator subunits that are not embryonic lethal led to the identification of an uncharacterized *dMed7^MI10755^* allele. MED7, a subunit of the “middle” module of the Mediator complex, plays a role in facilitating the assembly of the Mediator–Pol II holoenzyme (*58*). The *dMed7^MI10755^* allele features a Minos-based mutagenic gene trap cassette inserted in the 3′ untranslated region (3′UTR) region of *dMed7* locus (fig. S4A), which we validated by PCR using genomic DNA (fig. S4B). Homozygous *dMed7^MI10755^* mutants survive until third instar (fig. S4C). In these mutants, we observed an increase in the expression of *HeT-A* and *TART* transcripts (Fig. 3A and fig. S4D; *TAHRE* was not detectable), which correlated with a notable increase of telomere length as quantified using qPCR (Fig. 3B). Moreover, the *dMed7^MI10755^* mutant dominantly enhanced TPEs (Fig. 3C; quantified in fig. S1L), indicating an extension of heterochromatic telomeres in these mutants. Using HCR RNA-FISH, we observed that depletion of dMed7 specifically in salivary gland cells increased the expression of *HeT-A* and *TART* (Fig. 3E) compared to the control (Fig. 3D; quantified in Fig. 3I; images of single channels are shown in fig. S5).

Among the 30 Mediator complex subunits, MED14 and MED17 are essential central scaffold subunits necessary for assembling the small Mediator complex (*30*). To further test the involvement of the Mediator complex in regulating TR transcription, we depleted dMed14 and dMed17 in salivary gland cells and used the HCR RNA-FISH assay to detect TR transcripts. Similar to *dMed7*, depletion of *dMed14* (Fig. 3F), *dMed17* (Fig. 3G), or both *Cdk8* and *CycC* (Fig. 3H) in salivary gland cells also resulted in an up-regulation of *HeT-A* and *TART* expression (quantified in Fig. 3I and fig. S5). *TAHRE* transcripts were barely detectable in this assay (Fig. 3, D to I, and fig. S5). Together, these analyses reveal that depleting multiple subunits of the small Mediator complex leads to similar effects as the loss of *Cdk8* and *CycC*, resulting in elevated TR transcription in different types of cells. The simplest interpretation of these observations is that the large Mediator complex acts as a transcriptional repressor of TRs.

### Increased TR expression and elongated telomere length in *scalloped* mutants

Despite decades of research into the mechanisms by which TRs regulate telomere length in *Drosophila*, the specific transcription factors controlling TR transcription remain largely unknown. In the budding yeast *Saccharomyces cerevisiae*, two transcriptional activators—Ste12 and Tec1—are reported to regulate the expression of the Ty1 retrotransposons (*59*). In addition, certain nonessential yeast Mediator subunits also modulate Ty1 expression (*60*). Through a BLAST (Basic Local Alignment Search Tool) search, we identified Sd as a potential homolog of Tec1 in *Drosophila*; however, no homologs of Ste12 were found. Sd, a TEAD/TEF-family transcription factor that functions downstream of the Hippo signaling pathway, is crucial for organ size regulation and is of particular interest due to the frequent dysregulation of the Hippo signaling pathway in various human cancers (*61*–*63*). This, coupled with the fact that retrotransposons are key contributors to genetic variation and are often abnormally expressed and inserted in cancer (*64*, *65*), prompted us to investigate whether Sd/dTEAD plays a role in TR expression or telomere length maintenance.

**Fig. 4.**
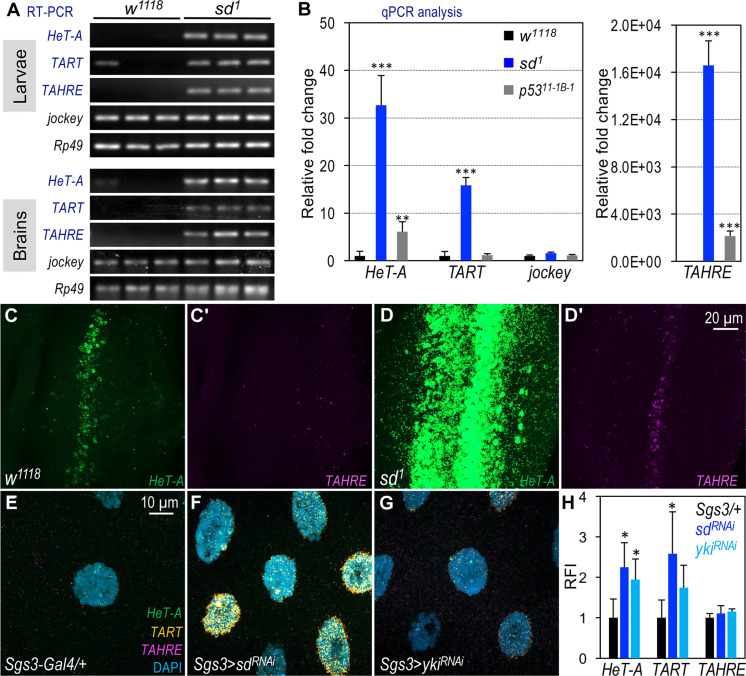
Effects of *sd* and *yki* mutations on TR expression and telomere length. (**A**) qRT-PCR analysis of TR transcript levels in both *sd^1^* homozygous mutant larvae and larval brain samples. The results are visualized via nucleic acid gel electrophoresis. (**B**) Telomere length measured by qPCR analysis using genomic DNA from *w^1118^* (control), *sd^1^* mutants, and *p53^11-1B-1^* mutant larvae. Statistical significance: **P < 0.01, ***P < 0.001 (one-tailed unpaired *t* tests). (**C** to **D′**) Detection of *HeT-A* [green; (C) and (D)] and *TAHRE* [magenta; (C′) and (D′)] mRNA transcripts in eye discs of the control (*w^1118^*; C/C′) and *sd^1^* mutant larvae [(D) and (D′)] using the HCR RNA-FISH assay. Scale bar, 20 μm. (**E** to **G**) Detection of *HeT-A* (green), *TART* (orange), and *TAHRE* (magenta) mRNA transcripts using the HCR RNA-FISH assay in salivary glands. Genotype details: (E) *Sgs3-Gal4/+*;*+* (control); (F) *Sgs3-Gal4/+*; *UAS-sd^RNAi^/+*; and (G) *Sgs3-Gal4/+*; *UAS-yki^RNAi^/+*. Scale bar, (E) 10 μm. (**H**) Quantification of the RFI of the samples shown in (E) to (G), with specific genotypes color-coded.

To examine the effect of the *sd* mutation on TR expression and telomere length, we conducted qRT-PCR and qPCR assays using *sd^1^* mutant larvae. The *sd^1^* mutation, induced by x-ray irradiation, is a hypomorphic allele resulting in fully viable *sd^1^* homozygotes that display a notched wing phenotype (*66*). Our analyses revealed a substantial up-regulation in mRNA levels of all three TRs in *sd^1^* mutant larvae (fig. S6, A and B) and dissected larval brains (Fig. 4A). In addition, the HCR RNA-FISH assay showed elevated TR expression in the eye discs of *sd^1^* mutants (Fig. 4, D and D′, and fig. S6F) compared to the control (Fig. 4, C and C′, and fig. S6E). Similarly, an increase of *HeT-A* expression was observed in *sd^1^* wing discs (fig. S6H′) relative to the control (fig. S6G′). Furthermore, the TR copy number in *sd^1^* mutant larvae was higher than that of *jockey* (Fig. 4B). Notably, when compared to *p53* mutants (*Drosophila TP53*), a known repressor of retrotransposon expression in *Drosophila,* zebrafish, and mammalian cells (*48*), *sd^1^* mutants exhibited a higher TR copy number (Fig. 4B). Visualization of polytene chromosomes in *sd^1^/+* larvae showed longer telomeres than those in the control (fig. S6D cf. fig. S6C; quantified in Fig. 1G). Similar to the mutants of *Cdk8^K185^*, *CycC^Y5^*, and *dMed7^MI10755^*, *sd^1^* also dominantly enhanced telomeric position variegation (fig. S7A). Collectively, these findings suggest that the loss of Sd/dTEAD increased TR expression and telomere length.

The transcriptional cofactor Yorkie (Yki) physically and genetically interacts with Sd/dTEAD (*67*–*69*). To further explore the roles of Sd and Yki in TR expression, we depleted them from salivary glands and examined TR expression using the HCR RNA-FISH assay. Compared to the control (Fig. 4E), depletion of either *sd* (Fig. 4F) or *yki* (Fig. 4G) increased the levels of *HeT-A* and *TART*, although the effect on *TAHRE* was less pronounced (fig. S7, B to D; quantified in Fig. 4H). These results suggest that both *sd* and *yki* act as negative regulators of *HeT-A* and *TART* transcription.

The effects on TR expression resulting from the depletion of *sd* and *yki* resemble the phenotypes observed upon depletion of multiple subunits of the Mediator complex. Mass spectrometry analyses of both *Drosophila* and cultured human cancer cells have shown that Yki and its human homolog YAP can bind multiple Mediator subunits, including MED1, MED12, MED14, MED15, MED19, MED23, MED24, and MED31 (*70*, *71*). We thus hypothesize that the large Mediator complex is recruited to the telomeric DNA through an Sd-Yki complex, collectively repressing the expression of the TRs, as further explored below.

### Key role of Rbf1 and E2F1-Dp in regulating TR transcription

Gene expression is regulated by a complex and dynamic interplay involving various DNA-bound activators and repressors, interacting protein complexes, RNA polymerases with their associated general transcription factors, chromatin modifiers, as well as long-range chromatin dynamics and genome organization (*72*–*74*). The identification of the Mediator complex and the Sd-Yki complex as negative regulators of TR transcription led us to explore whether TR transcription relies on specific transcriptional activators. We considered E2F1 and its dimerization partner Dp as potential activators of TR expression for several reasons. First, TRs are primarily expressed in proliferating cells, particularly during early S phase (*54*). The E2F1-Dp complex, along with other components of the Rb-E2F pathway, is essential for regulating the transcription of genes involved in DNA replication during the G_1_-to-S phase transition of the cell cycle (*34*, *35*). Second, CDK8 interacts with E2F1 and inhibits E2F1-dependent gene expression in *Drosophila* (*41*). Third, E2F1 can compete with Yki for binding to Sd, forming an E2F1-Sd repressor complex that controls cell survival and organ size (*75*). Moreover, the *Drosophila* RB ortholog, Rbf1 (*76*), was recently reported to coimmunoprecipitate with Yki in S2R^+^ cells (*77*).

**Fig. 5.**
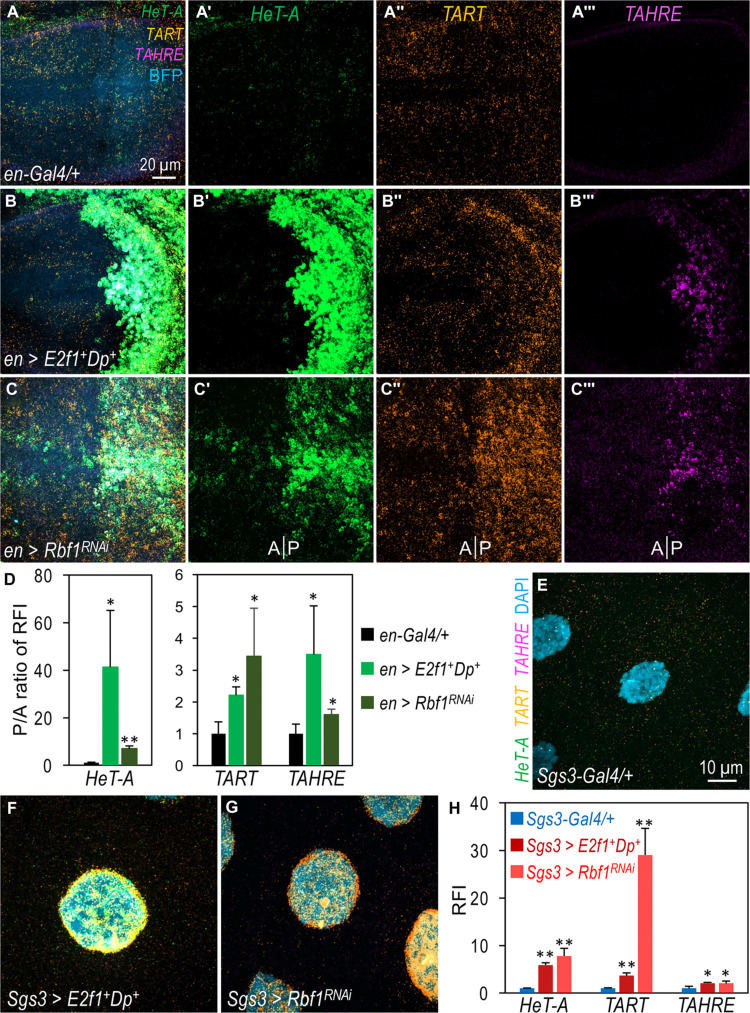
The role of RBF1-E2F1-Dp complex in regulating TR expression. mRNA transcripts of *HeT-A* [green; (A′) to (C′)], *TART* [orange; (A′) to (C″)], and *TAHRE* [magenta; (A‴) to (C‴)] in wing discs (**A** to **D**) and salivary glands (**E** to **H**) were detected using the HCR assay. Overexpression of E2F1-Dp or depletion of Rbf1 in the posterior compartment of the wing discs led to increased mRNA levels of all three TRs. [(D) and (H)] Quantification of the results in (A) to (C) and (E) to (G), respectively. Statistical significance: *P < 0.05, **P < 0.01 (one-tailed unpaired *t* tests). Detailed genotypes: [(A), control] *en-Gal4/+*; *UAS-BFP/+*; (B) *en-Gal4/+*; *UAS-E2f1^+^ UAS-Dp^+^/UAS-BFP*; (C) *en-Gal4/+*; *UAS-Rbf1^RNAi^/UAS-BFP*; (E) *Sgs3-Gal4/+*;*+* (control); (F) *Sgs3-Gal4/+*; *UAS-E2f1^+^ UAS-Dp^+^/+*; and (G) *Sgs3-Gal4/+*; *UAS-Rbf1^RNAi^/+*. Scale bars, 20 μm in (A) applies to (A) to (C); 10 μm in (E) applies to (E) to (G).

To investigate the role of the Rb-E2F pathway in telomere biology, we first examined whether the overexpression of E2F1-Dp affects TR expression. Using HCR RNA-FISH, we observed that the expression of the three TRs in the posterior compartment of wing discs was increased when E2F1 and Dp were coexpressed using *en-Gal4* (Fig. 5B cf. the control in Fig. 5A; quantified in Fig. 5D). This suggests that gain of E2F1-Dp is sufficient to drive TR expression. Since Rbf1 acts as a repressor of E2F1-Dp activity during the G_1_ phase of the cell cycle (*34*, *35*), we hypothesized that depleting Rbf1 would also stimulate TR transcription. Depletion of Rbf1 in wing discs using *en-Gal4* resulted in a substantial induction of TR expression (Fig. 5C; quantified in Fig. 5D). In salivary gland cells, both the overexpression of E2F1-Dp (Fig. 5F) and the depletion of Rbf1 (Fig. 5G) led to a strong up-regulation of all three TRs compared to the control (Fig. 5E and fig. S8; quantified in Fig. 5H). These observations collectively indicate that the activation or gain of E2F1-Dp is sufficient to stimulate TR transcription in both diploid wing disc cells and polyploid salivary gland cells.

**Fig. 6.**
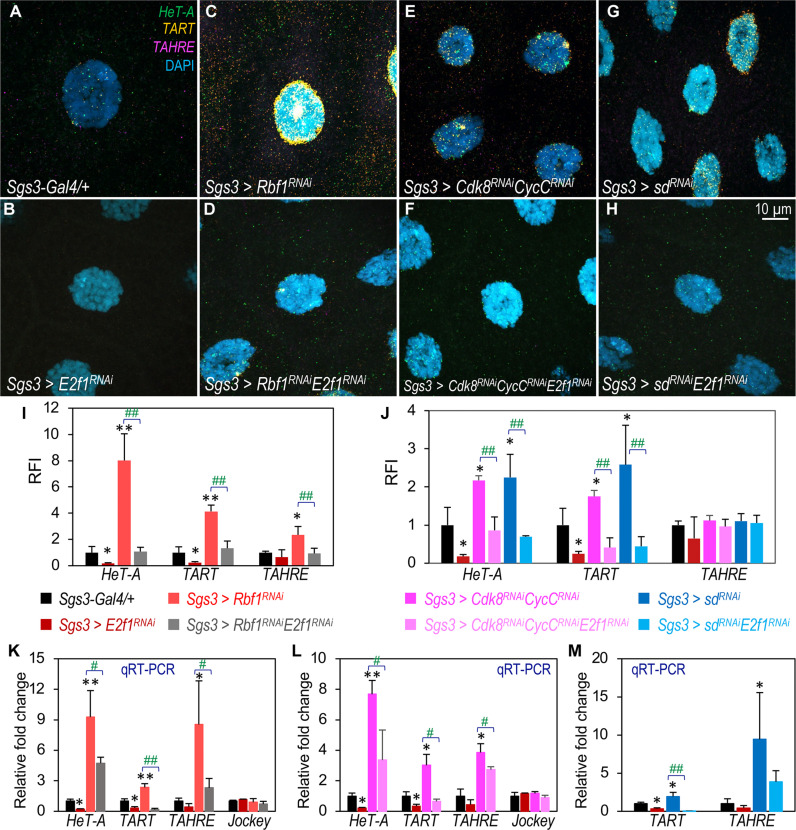
E2F1 dependency in TR expression upon depletion of Rbf1, Cdk8-CycC, and Sd/dTEAD. (**A** to **H**) Detection of mRNA transcripts of *HeT-A* (green), *TART* (orange), and *TAHRE* (magenta) in salivary glands using the HCR RNA-FISH assay. The detailed genotypes are as follows: (A) *Sgs3-Gal4/+*; *+* (control); (B) *Sgs3-Gal4/+*; *UAS-E2f1^RNAi^/+*; (C) *Sgs3-Gal4/+*; *UAS-Rbf1^RNAi^/+*; (D) *Sgs3-Gal4/+*; *UAS-Rbf1^RNAi^/UAS-E2f1^RNAi^*; (E) *Sgs3-Gal4/+*; *UAS-Cdk8^RNAi^ CycC^RNAi^/+*; (F) *Sgs3-Gal4/+*; *UAS-Cdk8^RNAi^ CycC^RNAi^/UAS-E2f1^RNAi^*; (G) *Sgs3-Gal4/+*; *UAS-sd^RNAi^/+*; and (H) *Sgs3-Gal4/+*; *UAS-sd^RNAi^/UAS-E2f1^RNAi^*. The scale bar, (H) 10 μm. (**I** to **J**) Quantification of the RFI for each genotype, with specific color-coding for clarity. (**K** to **M**) Relative fold change in mRNA levels in dissected salivary glands as determined by qRT-PCR. Genotypes in these panels are color-coded as in (I) and (J). Statistical significance: * indicates comparisons with the control (*Sgs3-Gal4/+*;*+*), while # indicates comparisons with the respective controls noted in the chart. *P < 0.05, ** or ##*P* < 0.01 (one-tailed unpaired *t* tests).

Using HCR RNA-FISH, we found that the depletion of E2F1 in salivary gland cells potently reduced the expression of *HeT-A* and *TART* (Fig. 6B, cf. the control in Fig. 6A and fig. S9; quantified in Fig. 6I). The expression of endogenous *TAHRE* was notably lower compared to *HeT-A* and *TART* (Fig. 6A). These results indicate that E2F1 is required for the transcription of *HeT-A* and *TART*, two major TRs. Next, we asked whether the effect of *Rbf1* loss on TR expression is dependent on E2F1. As shown in Fig. 6D, co-depleting *Rbf1* and *E2f1* abolished the effects of Rbf1 reduction (Fig. 6C) on TR expression (fig. S9; quantified in Fig. 6I). These results from the HCR RNA-FISH assay were validated using qRT-PCR on dissected salivary glands of the same genotypes (Fig. 6K), with *jockey* serving as a negative control. Similar results were observed with Dp depletion alone or with the co-depletion of Dp and Rbf1 (fig. S10B). Overall, these findings support the model for the inhibitory role of Rbf1 on E2F1-Dp–dependent transcription (*34*, *35*), suggesting a crucial role of the Rb-E2F pathway in regulating TR transcription.

### Essential role of E2F1-Dp in mediating the transcriptional inhibition of TRs by CDK8 and Sd/dTEAD

To test whether the effects of CDK8 and Sd/dTEAD loss on TR expression were also dependent on E2F1, we simultaneously depleted E2F1 along with either CDK8 or Sd in salivary gland cells and assessed TR expression using an HCR RNA-FISH assay. As expected, depleting *Cdk8-CycC* increased the expression of *HeT-A* and *TART* (Fig. 6E; quantified in Fig. 6J) compared to the control (Fig. 6A). However, co-depleting both CDK8-CycC and E2F1 strongly mitigated the effects of CDK8-CycC depletion on *HeT-A* and *TART* expression (Fig. 6F and fig. S11). Similar effects were observed when Dp was depleted along with CDK8-CycC (fig. S10C; quantified in fig. S10E). These findings were further validated using qRT-PCR assays on dissected salivary glands (Fig. 6L). Thus, these data suggest that the effect of CDK8-CycC reduction on *HeT-A* and *TART* expression is dependent on E2F1-Dp.

Next, we extended our analysis to determine whether the effect of Sd depletion on TR expression also depends on E2F1-Dp. Depletion of Sd alone led to increased expression of *HeT-A* and *TART* (Fig. 6G). In contrast, co-depleting Sd with E2F1 (Fig. 6H, and fig. S11) or Dp (fig. S10) suppressed this up-regulation (quantified in Fig. 6J and fig. S10E). The effects on *TART* were confirmed by qRT-PCR (Fig. 6M), although the results for *HeT-A* and *TAHRE* were inconclusive because of large variations. In summary, these observations suggest that the effects of disrupting the Mediator complex or Sd on TR expression are dependent on E2F1-Dp. This indicates that the Mediator complex modulates telomere length homeostasis by restraining the expression of TRs through the transcription factors Sd/dTEAD and E2F1-Dp.

### Direct binding of CDK8, Dp, and Sd to telomeric HTT repeats

To examine whether the Mediator complex, E2F1-Dp, and Sd/dTEAD directly or indirectly regulate TR transcription, we used the CUT&RUN, a sensitive high-throughput method for mapping genomic binding of chromatin-associated proteins (*78*). Identifying direct binding of these factors to the HTT repeat would suggest a direct regulatory mechanism. However, a notable technical challenge was the lack of ChIP (chromatin immunoprecipitation)–grade antibodies specific to CDK8, E2F1-Dp, and Sd. To overcome this, we used CRISPR-Cas9 to introduce an EGFP tag into the endogenous *Cdk8* gene (see Materials and Methods). For Sd and Dp, we used two preexisting EGFP-tagged lines where the endogenous genes were tagged with EGFP. Notably, homozygotes of all three strains—*Cdk8^EGFP^*, *Dp^EGFP^*, and *Sd^EGFP^*—were fully viable and fertile, suggesting that the introduction of EGFP tags does not disrupt the normal functions of these three proteins.

**Fig. 7.**
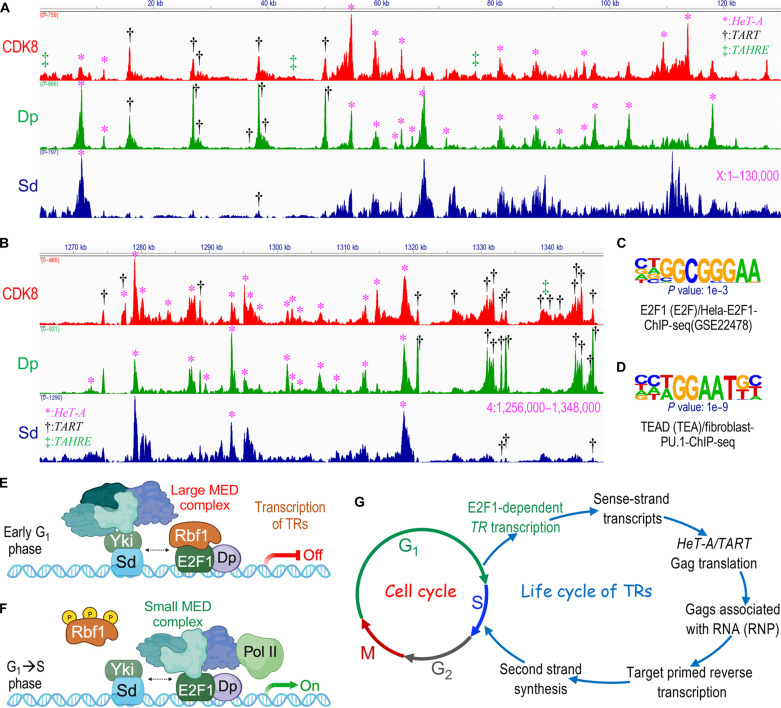
Direct binding of CDK8, Dp, and Sd/dTEAD to telomeric HTT repeats. Genomic tracks illustrate the binding peaks of CDK8 (red), Dp (green), and Sd/dTEAD (dark blue) at the telomeres of two chromosomal regions: XL [(**A**) with the chromosome end on the left] and 4R [(**B**) with the chromosome end on the right]. To identify specific sequences within the XL (A) and 4R (B) telomeres, individual BLAST searches were performed. The locations of the three TRs are marked with different symbols (* for *HeT-A*, † for *TART*, and ‡ for *TAHRE*). (**C** and **D**) Identification of two top-matching consensus motifs from the Dp (C) and Sd/dTEAD (D) binding sequences through the Homer Known Motif Enrichment analysis (50 bp). The motifs identified are “E2F1 (E2F)/Hela-E2F1-ChIP-seq (GSE22478)” for Dp (C) and “TEAD (TEA)/Fibroblast-PU.1-ChIP-seq” for Sd/dTEAD (D). (**E** and **F**) Proposed model for the transcriptional regulation of TRs: (E) During the early G_1_ phase, the large Mediator complex represses TR transcription via Sd and E2F1-Dp. (F) As the cell transitions through the G_1_-S phase, the small Mediator complex becomes crucial for activating TR transcription, mainly through E2F1-Dp (F). (**G**) Schematic model depicting the transcriptional coupling of the TR life cycle with the host cell cycle via E2F1-Dp, ensuring chromosome integrity in *Drosophila*. RNP, telomere ribonucleoprotein; TRs, telomere-specific retrotransposons.

Using wing discs from these EGFP-tagged lines, we conducted the genome-wide analysis of the binding profiles of CDK8, Dp, and Sd by CUT&RUN assay. Specifically, we focused on binding at the telomeres of the *X* chromosome’s left arm (*XL*) and chromosome 4’s right arm (*4R*), as assembled by the *Drosophila* Heterochromatin Genome project (*45*). Our analyses of the *XL* telomere region, approximately 130 kb in length, revealed around 20 called peaks of CDK8 binding, 23 called peaks of Dp binding, and two called peaks of Sd binding. Despite the lower number of called peaks for Sd compared to CDK8 and Dp, a substantial overlap was observed among the binding peaks of these three proteins (Fig. 7A). Several non-called Sd peaks, which were not identified by bioinformatic algorithms, also overlapped with common CDK8- and Dp-binding peaks (Fig. 7A), indicating the possible colocalization of all three proteins at many sites.

Because these telomeric regions are not annotated in the *Drosophila* genome assembly Release r5.9, we conducted BLAST searches using the sequences associated with each of the identified peaks. Our results revealed a substantial overlap between most of these peaks and the three TRs: *HeT-A*, *TART*, and *TAHRE* (Fig. 7A). This supports our model that CDK8, Dp, and Sd directly regulate TR transcription. Similarly, analysis of the *4R* telomere, spanning approximately 92 kb, revealed more than 30 called peaks of CDK8 binding, more than 20 called peaks of Dp binding, and 5 called peaks of Sd binding, with substantial overlap between them (Fig. 7B). As with the *XL* telomere, all of these called peaks corresponded with the three TRs (Fig. 7B). Notably, multiple *HeT-A* and *TART* elements cluster at the telomeres of *XL* and *4R*, similar to patterns depicted previously (*45*). We also observed binding of these three proteins in the telomere regions of the chromosome *2L* (fig. S12A) and *3L* (fig. S12C), but further analysis was not possible because of the lack of an assembly of the repetitive DNA sequence in these regions. Similarly, CUT&RUN sequencing data could not be mapped for telomeres of *2R* (fig. S12B), *3R* (fig. S12D), and *4L* (fig. S12E). As a positive control, we confirmed the distinct binding patterns of CDK8, Dp, and Sd on their common target gene, *cyclin E* (fig. S12F).

To further explore binding specificity, motif enrichment analyses were performed on sequences associated with Dp-called peaks, revealing consensus motifs for E2F1 (Fig. 7C) and E2F3 (fig. S12G), characterized by the consensus sequence of GCGGGAA or its reverse complement TTCCCGC, resembling known *Drosophila* E2F1-binding motifs (*79*, *80*). Similarly, analyses of Sd-binding sequences identified TEAD (Fig. 7D) and TEAD2 (fig. S12H) motifs, characterized by the consensus sequence [T/A]GGAAT[G/T].

Further examination of these consensus motifs identified two potential E2F1-binding sites and several TEAD-binding sites in the 3′UTR of *HeT-A* elements (figs. S13A, S14, and S15). Comparable analyses identified several potential E2F1- and TEAD-binding sites in both 5′UTR and 3′UTR of *TART-A* element (figs. S13B and S16), as well as in the 3′UTR of *TART-B* (figs. S13B and S17), *TART-C* (figs. S13B and S18), and *TAHRE* (figs. S13C and S19). An E2F1-binding motif is also found in the 5′ repeat region of the *TART-C* element (figs. S13B and S18). These binding patterns of E2F and Sd/dTEAD are particularly intriguing as they are located near the transcription start sites of the full-length sense strand of *HeT-A* (figs. S13A, S14, and S15) and *TAHRE* (figs. S13C and S19), as well as the short sense strand of *TART-A/B/C1* elements (fig. S13B) and the antisense RNA start site of *TART-A/B* (fig. S13B) (*2*). Multiple E2F1-binding motifs, TT[C/G][C/G]CGC, were identified within these TRs (highlighted in magenta in figs. S14 and S16 to S19).

To validate the CUT&RUN results, we performed a ChIP-qPCR assay using *Dp^EGFP^* or *sd^EGFP^* homozygous embryos. As shown in fig. S20 (A and B), there was notable enrichment of Dp^EGFP^ or Sd^EGFP^ at multiple loci within *HeT-A*, *TART*, and *TAHRE* elements, using samples from *w^1118^* embryos as negative controls (fig. S20C). In addition, a comparison of our Dp^EGFP^ CUT&RUN data with a previous ChIP sequencing (ChIP-seq) study using an anti-dDp antibody in pupal muscle tissue (*80*) revealed a substantial similarity in Dp enrichment peaks at the two telomeric regions (fig. S20, D and E). Collectively, these observations provided compelling evidence for the direct regulation of TR transcription by CDK8, E2F1-Dp, and Sd/dTEAD.

## DISCUSSION

Telomeres protect chromosome ends and are crucial for genome stability. Our study suggests that the large Mediator complex is recruited to telomeric repeats through its interaction with Sd/dTEAD and E2F1-Dp, thereby repressing TR transcription and modulating telomere length in *Drosophila*. Depletion or loss of Sd, Rbf1, and multiple Mediator subunits, or the overexpression of E2F1-Dp, stimulates TR transcription. The effects of Sd/dTEAD, Rbf1, and CDK8 depletion are dependent on E2F1-Dp. Our CUT&RUN analyses reveal direct binding of CDK8, Dp, and Sd to the telomeric HTT repeats. These findings support a model where, during early G_1_, the large Mediator complex directly represses TR transcription via Sd/dTEAD and E2F1-Dp (Fig. 7E). In contrast, during the G_1_-S phase transition, the small or core Mediator complex activates TR transcription through E2F1-Dp (Fig. 7F). This model suggests that TR transcription is synchronized with the G_1_-S transition of the host cells, occurring just before telomere elongation in the S phase (Fig. 7G). This precise coupling between the TR life cycle and the host cell-cycle machinery via E2F1-Dp establishes a symbiotic relationship that benefits both.

### Identification of three TRs as targets of E2F1-Dp

An unexpected discovery of our study is that the three TRs are direct transcriptional targets of E2F1-Dp, the master transcription factor that controls the G_1_-S phase transition (*34*, *35*). Several key observations support this: The overexpression of wild-type E2F1 and Dp strongly stimulates TR transcription in various cell types; TR transcription is repressed by Rbf1, with its depletion up-regulating TR expression; and knocking down either E2F1 or Dp reduces TR transcription, particularly for *Het-A* and *TART*. Furthermore, depleting E2F1 or Dp effectively abolishes the effect of Rbf1 reduction on TR up-regulation. Collectively, these results establish a strong causal link between E2F1-Dp activity and the regulation of TR transcription.

The temporal and spatial expression patterns of TRs in developing tissues closely correlate with the cell division cycle and E2F1 activity. For example, TR expression is observed in cells located behind the morphogenetic furrow in eye discs (*81*) and is notably elevated in *sd^1^* mutants (Fig. 4, D and D′, and fig. S6, F to F‴). In wing discs, TR expression is reduced in the anterior compartment of the nonproliferating cell zone compared to the asynchronously dividing cells outside this zone (Fig. 2H and fig. S2G). These observations suggest a developmental context in which E2F1-Dp regulates TR expression in actively growing, patterning, and proliferating tissues. Furthermore, the direct binding of Dp to the TRs in the telomeres of *XL* and *4R* (Fig. 7), along with the identification of E2F1 consensus binding sites in all three TRs (figs. S13 to S19), provides further validation for their classification as bona fide E2F1-Dp targets in vivo. Collectively, these observations support a model in which E2F1-Dp plays a crucial role in regulating TR transcription in various cellular contexts during development, affecting both diploid mitotic and polyploid endoreplicating cells.

Previous studies have shown that pRB-E2F complexes, along with histone deacetylases HDAC1 and HDAC2, repress the transcription of the retrotransposon LINE-1 (long interspersed nuclear elements, or L1 elements) in cultured mouse and human cells (*82*). In mouse embryonic fibroblast cells lacking Rb family members (pRb, p107, and p130) or in cells treated with the HDAC inhibitor trichostatin A, LINE-1 expression increases, accompanied by a decrease in epigenetic silencing markers such as histone H3 and H4 trimethylation and H3 deacetylation (*82*). In addition, pRB, E2F1, and E2F4 are enriched at LINE-1 elements, and E2F-binding sites are identified in their 5′UTR region (*82*). The loss of Rb family members also reduces HDAC1 and HDAC2 binding at the LINE-1 promoter, suggesting that Rb proteins may repress LINE-1 by recruiting these deacetylases (*82*). Furthermore, a pRB-EZH2 complex has been shown to repress LINE-1 and other repetitive DNA sequences, including simple repeats, satellites, and endogenous retrovirus in mammalian cells (*83*). pRB associates with these sequences in a cell cycle–independent manner, while the enrichment of H3K27me3 and recruitment of EZH2 to these elements is pRB dependent (*83*). This suggests that E2F1 may recruit the pRB-EZH2 complex to LINE-1 and other repetitive sequences, thereby silencing their expression through H3K27me3 (*83*).

Together, these studies establish the role of pRB and E2F family members in repressing the transcription of LINE-1 elements and other repetitive DNA sequences. The loss of RB family members, likely along with their cofactors such as EZH2 and HDACs, leads to altered histone modifications and the derepression of these transposable elements (*82*, *83*). However, since E2F1, E2F2, and E2F3a are transcriptional activators, while E2F3b, and E2F4-E2F8 are transcriptional repressors (*34*, *35*), it remains unclear whether the derepression of LINE-1 and other repetitive elements caused by pRB loss depends on the activator functions of E2F1, E2F2, and E2F3a in mammals, similar to what we have observed in *Drosophila*.

### Dual role of the Mediator complex in TR transcription

The CDK8 module binds to the small Mediator complex to form the large Mediator complex, which typically represses Pol II–dependent transcription (*25*, *26*). Depletion of multiple large Mediator subunits leads to up-regulation of TR transcription, indicating its negative regulatory role. In *Drosophila*, five Mediator subunits—MED1, MED15, MED19, MED23, and MED31—have been shown to bind to the Yki transcription factor (*70*). Similarly, in humans, Mediator subunits MED12, MED14, MED23, and MED24 interact with YAP in bile duct carcinoma cells (*71*). This mechanism appears to be conserved, as ChIP-seq analyses reveal that 87% of YAP-binding sites overlap with MED1-binding sites (*71*). Moreover, the Yki homolog TAZ interacts with MED15 in human embryonic stem cells (*84*). Given that depleting either *sd* or *yki* up-regulates TR transcription, similar to depleting subunits of the large Mediator complex, the most parsimonious model suggests that Sd-Yki facilitates the recruitment of the large Mediator complex to HTT repeats.

Furthermore, depletion of Rbf1, which represses E2F1-Dp activity, strongly up-regulates TR expression. The effect of Mediator complex depletion on TR transcription is considerably weaker compared to Rbf1 depletion. Although the efficiency and kinetics of RNAi-mediated depletion of these factors might influence the effects on TR transcription, we postulate that E2F1-dependent transactivation of TRs likely relies on the small Mediator complex. Depleting small Mediator subunits would disrupt both TR repression by the large Mediator complex and E2F1-dependent TR transactivation via the small Mediator complex. Our previous work demonstrated that two subunits of the Mediator complex, CDK8 and CycC, negatively regulate E2F1-dependent transcription (*41*), likely through CDK8-mediated phosphorylation of E2F1 (*41*, *42*).

The polyploid nuclei of salivary gland cells in *Cdk8* mutants (e.g., Fig. 1D) or with CDK8 depletion (e.g., Fig. 3H) appear smaller compared to their corresponding control samples. This observation is not an artifact, as E2F plays a critical role in regulating endoreplication of polytene chromosomes, and DNA content is known to correlate with cell growth and size (*33*). Therefore, factors like Mediator subunits that regulate E2F activities are expected to influence DNA endoreplication in salivary gland cells, although the primary focus of this study is on TR transcription.

Our findings favor the model in which the small Mediator complex participates in E2F1-dependent TR transcription. It is plausible that the Mediator complex plays a dual role in regulating TR transcription. During the early G_1_ phase, the large Mediator complex represses TR transcription, primarily via Sd-Yki and Rbf1-E2F1-Dp complexes (Fig. 7E). In contrast, during the G_1_-S phase transition and into early S phase, the small Mediator complex becomes essential for activating TR transcription, primarily through E2F1-Dp (Fig. 7F). Further studies are required to identify specific Mediator subunits interacting with E2F1 or Dp.

### Context-dependent interaction between Sd-Yki and Rbf1-E2F1-Dp complexes

Sd/dTEAD, a key transcription factor downstream of the Hippo signaling pathway, controls organ size and is often dysregulated in human cancers (*61*, *62*). The interplay between the Rb-E2F1 network and Hippo signaling regulates cell-cycle exit and apoptosis (*75*, *85*). In eye imaginal discs, E2F1 synergizes with Yki-Sd to activate shared target genes, leading to excessive cell proliferation and tissue growth (*85*). In contrast, in wing discs, E2F1 represses the expression of Yki targets such as *Diap1*, *expanded*, and *bantam*, thereby regulating apoptosis and organ size (*75*). This mechanism involves competition between E2F1 and Yki for binding to Sd/dTEAD, resulting in the formation of the E2F1-Sd repressor complex, with Rbf1 modulating this process by reducing the interaction between E2F1 and Sd (*75*). This molecular mechanism is conserved in human cells (*75*). These findings suggest that E2F1 interferes with the binding of Yki/YAP to Sd/dTEAD, thereby suppressing the expression of Yki-YAP target genes (*75*). However, the interplay between the Rbf1-E2F1 and Hippo pathways is further complicated by the fact that *dE2f1* is a transcriptional target of Sd-Yki (*69*, *75*). This potential feedback loop likely regulates the activities of both pathways. If so, the intricate interplay between Rbf1-E2F1-Dp and Sd-Yki is probably influenced by the specific promoter structures of their shared target genes and the particular contexts of the biological processes they regulate. Supporting this notion, Yki-Sd and E2F1 can coactivate common target genes such as *Dachs*, *Dp*, and *PCNA* in eye discs (*85*).

Our study demonstrates that Rbf1-E2F-Dp and Sd-Yki regulate TR transcription and telomere homeostasis. Specifically, Sd/dTEAD and Yki act as negative regulators of TR expression, while E2F1 and Dp are required for TR transcription. We propose that this protein complex responds to growth factors and other extracellular stimuli that regulate cell proliferation. It would be intriguing to explore whether additional components of the Hippo signaling pathway also regulate TR transcription through the Mediator complex.

### Dominant effect of *Cdk8* and *CycC* mutations on telomere length

While changes in TR transcription are transient, substantial changes in telomere lengths in *Drosophila* occur over extended periods, ranging from months to years. Longer telomeres have been observed in the *Cdk8^K185^* and *CycC^Y5^* mutant alleles generated before 2007 (*36*), as well as in the *Cdk8*^Δ*mCherry*^ allele created in 2016 (this study). Our analysis of *Cdk8*^Δ*mCherry*^ mutant larvae over a 5-year period (2017, 2022, and 2024) reveals a progressive increase in telomere length, which remains stable in the rescued larvae (Fig. 2C). This suggests that long-term genomic effects on telomere lengths require prolonged observation across multiple generations. Moreover, qPCR analyses of genomic DNA reveal longer telomeres in *CycC^Y5^*, *dMed7^MI10755^*, or *sd^1^* homozygous mutant larvae. These results are further supported by confocal imaging of polytene chromosomes from heterozygous larvae and the dominant enhancement of telomeric position variegation by the mutant alleles. Since homozygous mutants of *Cdk8^K185^*, *Cdk8*^Δ*mCherry*^, *CycC^Y5^*, and *dMed7^MI10755^* are lethal, the observation of longer telomeres in these mutants suggests a dominant effect on telomere length, indicating that TR transcription is sensitive to the dosage of the Mediator complex.

Our previous study demonstrated that *Cdk8* mutation has a dominant effect on the transcription of E2F1 target genes (*41*). In a dominant modifier genetic screen using *E2f1^RNAi^* phenotypes, *Cdk8* was identified as a strong suppressor. Reducing CDK8 levels by half suppressed *E2f1^RNAi^*-induced phenotypes in *Drosophila* eyes and wings, indicating an increase in E2F1 activity in a *Cdk8* heterozygous background (*41*). CDK8 directly interacts with and phosphorylates E2F1, thereby inhibiting its activities (*41*, *42*). Thus, reducing CDK8 levels by half may stabilize the remaining E2F1 proteins, rescuing the *E2f1^RNAi^* phenotypes (*41*). These observations show the dominant effect of *Cdk8* mutation on E2F1 activity and target gene expression.

Our current findings demonstrate that E2F1 directly regulates TR transcription. Overexpression of E2F1-Dp or depletion of the E2F1-Dp repressor Rbf1 stimulates TR transcription, whereas the depletion of E2F1 or Dp abolishes it. Dp directly binds to telomeric regions, where E2F-binding motifs have been identified in all three TR types, indicating that these TRs are bona fide E2F1 target genes, similar to other E2F1-regulated genes essential for DNA replication during the S phase. Furthermore, TR expression increases in heterozygous *Cdk8* or *CycC* mutants during the G_1_-S phase transition or the endoreplication cycle in salivary gland cells. This up-regulation may contribute to telomere elongation over successive generations.

The correlation between the relative fold changes in TR transcripts is generally higher than that observed in qPCR data using genomic DNA samples from homozygous mutant larvae of *Cdk8^K185^*, *Cdk8*^Δ*mCherry*^, *CycC^Y5^*, *dMed7^MI10755^*, or *sd^1^*. This discrepancy likely stems from the inherent strengths and limitations of the complementary methods used to assess TR transcription and telomere length. Therefore, the focus should be on identifying robust changes relative to controls rather than on precise fold changes. Moreover, increased TR mRNA expression does not always lead to higher rates of de novo transposition events and net telomere lengthening. Previous studies have shown that higher retrotransposon expression does not necessarily correlate with increased somatic transposition in *Drosophila* (*86*, *87*). Although these studies did not specifically examine TRs (*86*, *87*), the substantial telomere lengthening observed in those mutants suggests potential effects on the accessibility of telomere ends to the telomere ribonucleoprotein. Perhaps changes in chromatin structure in these mutants during the cell cycle might promote telomere conformations conducive to de novo telomere addition.

### Coupling of TR life cycle with host cell-cycle machinery

The RB-E2F network is crucial for regulating the G_1_-S transition (*34*, *35*). Identifying the three TRs as direct targets of E2F1 elucidates how the TR life cycle is coupled with the host cell-cycle machinery (Fig. 7G). The TR life cycle relies on various host components, including RNA polymerase, activating transcription factors, and the synthesis of Gag proteins encoded by the TRs, as well as the reverse transcriptase encoded by *TART* and *TAHRE* (*1*–*3*). Given the influence of the cell cycle on various cellular processes, it is logical that the TR life cycle would adapt to the host cell cycle to ensure the success of both TRs and host cells.

Most eukaryotes resolve the “end replication problem” by using telomerase to add telomere repeats to chromosome ends. However, in some dipteran insects like *Drosophila*, an alternative mechanism involving TR retrotransposition has evolved. Despite their differences, both mechanisms couple telomere elongation with S phase replication. For instance, *HeT-A* mRNA is most abundant during S phase (*52*, *53*), and Orf1p (from *HeT-A*) is primarily detected at the G_1_-S boundary and early S phase, but not during M phase (*54*). The direct regulation of TR transcription by E2F1-Dp suggests a model in which telomere addition is synchronized with cell-cycle progression. This illustrates a robust coupling between the TR life cycle and host cell-cycle machinery, ensuring effective telomere maintenance and genomic stability throughout multiple rounds of cell divisions (Fig. 7G).

Studies of the “noncanonical” telomeres in *Drosophila* have provided broader insights into chromosome biology and the coevolution of retrotransposons and host genomes (*2*). Our findings demonstrate that TR transcription is tied to the G_1_-S phase transition and occurs before retrotransposition during DNA replication in S phase. This finding illustrates the close integration of TR life cycle with the cell-cycle machinery of the host cells.

### Limitations of the study

While the data document a coupling between the TR life cycle and host cell-cycle machinery in somatic cells, such as diploid imaginal disc cells and polyploid salivary gland cells, mediated by several shared key factors, their roles in germline cells were not examined in this study. The longer telomeres observed in mutant alleles of *Cdk8*, *CycC*, *Med7*, and *sd* over generations suggest that a similar mechanism may operate in germline cells, warranting further investigation. In the female germ line, TR transcription—particularly of *HeT-A* and *TAHRE*—is repressed by the Piwi-piRNA complex (*14*–*17*), a pathway that is inactive in somatic cells (*18*). It remains unclear whether the factors identified in this study coordinate with the Piwi-piRNA pathway to regulate TR transcription in germline stem cells. Mutations in DNA or chromatin-binding proteins, such as BEAF32, Chro, DREF, JIL-1, Trf2, Woc, and Z4/Pzg (*19*–*22*) have been found to disrupt TR expression. More research is needed to understand how these factors are orchestrated to control different aspects of TR transcription in both somatic and germline cells.

## MATERIALS AND METHODS

### Fly stocks and maintenance

Flies were maintained at 25°C on a standard medium consisting of cornmeal, molasses, and yeast. The *w^1118^ Drosophila* strain was used as the control group. Null alleles of *Cdk8* (*FRT80B Cdk8^K185^/TM3 Sb^1^*) and *CycC* (*FRT82B CycC^Y5^/TM3 Sb^1^*) were provided by H.-M.Bourbon. The *TM3 Sb^1^* balancer chromosomes in these strains were replaced by *TM6B Tb^1^* balancer chromosomes to facilitate the identification of homozygous mutant larvae. Subsequently, these strains were outcrossed with the *w^1118^* line for over six generations at the early stage of this study (*37*). The *Cdk8^K185^ CycC^Y5^/TM6B Tb^1^* stain was obtained through genetic recombination. For analyses involving these null alleles, the *w^1118^* strain was used as the control. The *118E-15* strain used for TPE assays was obtained from L. Wallrath. The specific *Drosophila* strains and their genotypes are listed in table S1.

### Analyses of polytene chromosomes

To analyze larval polytene chromosomes, we followed the established procedure as described previously (*37*).

### Generation of the *Cdk8*Δ*^mCherry^* and *Cdk8^EGFP^* alleles using the CRISPR-Cas9 technique

To generate the *Cdk8*^Δ*mCherry*^ allele, we used CRISPR-Cas9–mediated homology-directed repair approach (*88*). We designed two guide RNAs (*dCdk8-sgRNA1*: AACACAGCCTTAACCAGGGA and *dCdk8-sgRNA2*: TCGTTGAAATATCTTTCCGA) using the online CRISPR design tool available at https://www.flyrnai.org/crispr/. These single-guide RNAs (sgRNAs) were designed to create double-strand breaks near the transcription start and termination sites of the *Cdk8* gene. Next, we inserted the sgRNAs into the *U6b*-sgRNA-short vector following the procedure as previously described (*89*). Meanwhile, we constructed the *dCdk8-4XP3-mCherry* donor vector as previously described (*88*). For the injection process, we combined the plasmid mix as follows: *dCdk8-sgRNA1* at 75 ng/μl, *dCdk8-sgRNA2* at 75 ng/μl, and *dCdk8-4XP3-mCherry* at 100 ng/μl. These plasmids were injected into embryos obtained from *P{nos-Cas9}attP40* flies, resulting in the generation of the *Cdk8*^Δ*mCherry*^ mutants. To identify successful *Cdk8*^Δ*mCherry*^ mutants, we screened for the expression of mCherry in the eyes using a fluorescent microscope, followed by genotyping PCR and Sanger sequencing to confirm the desired mutations. Since the *Cdk8*^Δ*mCherry*^ allele was created in the *TB77* background [genotype: *y,sc,v*; (*51*)], the TB77 strain served as the control for analyses involving the *Cdk8*^Δ*mCherry*^ allele.

To create the *Cdk8^EGFP^* allele, we used the CRISPR Optimal Target Finder tool available at http://targetfinder.flycrispr.neuro.brown.edu to design two sgRNAs. These sgRNAs were positioned near the transcription start site and transcription termination site to replace the entire *dCdk8* gene. We then cloned those two sgRNAs into the *pCFD3-dU6:3gRNA* vector following the protocol outlined previously (*90*), and the primer redesign was facilitated using the NEB Builder tool, with the primer sequences provided in table S2 for reference. For the assembly of the doner constructs, we used the NEBuilder HiFi DNA Assembly Cloning Kit (NEB, #E5520). This involved integrating both upstream and downstream homology arms, each spanning 1000 base pair (bp), along with the last intron-exon regions and the EGFP coding fragment, into the pGEM-T vector (Promega, A1360). The *Cdk8-EGFP* rescue construct, as described previously (*37*), served as the PCR template for amplifying the EGFP-fused *Cdk8* gene region. The injection of embryos with the donor vector and the two sgRNA constructs within the pCFD3 vector was conducted by Rainbow Transgenic Flies (https://rainbowgene.com/). Transgenic flies carrying the EGFP tag at the C terminus of CDK8 were identified through classical fly genetics methods. Validation was subsequently carried out by performing on extracted genomic DNA and confirming the results through sequencing.

### Western blot analysis

Western blot analysis was conducted using the same protocol as previously described (*37*). We used the following antibodies: anti-dCDK8 (polyclonal antibody from guinea pig, diluted at 1:1000, provided by H.-M. Bourbon) (*91*), and anti-actin monoclonal antibody (diluted at 1:4000, Thermo Fisher Scientific).

### RNA preparation, qRT-PCR, and RNA-seq analysis

Total RNA was isolated from third instar wandering larvae, as well as from dissected larval central nervous system or salivary glands, using TRIzol reagent (Invitrogen). The isolated RNAs were quantified, treated with deoxyribonuclease I to eliminate any genomic DNA contamination, and reverse transcribed using the High-Capacity cDNA Reverse Transcription kit from Applied Biosystems. In our qPCR analysis, we used SYBR Green from Applied Biosystems. The qPCR primers are listed in table S2, including those for *HeT-A*, *TART*, and *TAHRE*, as previously described (*92*), as well as *TAHRE-GAG* and *jockey-GAG* (*47*). *Rp49* was used as the internal control using the same primer pair reported earlier (*37*). For RNA-seq analysis, we followed the established protocol outlined in (*40*). The RNA-seq data generated for this study have been deposited in National Center for Biotechnology Information (NCBI’s) Gene Expression Omnibus (GEO) and are available under the GEO Series accession number GSE278499.

### DNA extraction and qPCR

For DNA extraction, five wandering stage larvae were collected and homogenized in 500 μl of squishing buffer, consisting of 0.1 M tris-HCl (pH 9.0), 0.1 M EDTA, and 1% SDS. The homogenate was incubated at 70°C for 30 min. Next, 70 μl of 8.0 M potassium acetate was added to the homogenate, and the samples were left on ice for an additional 30 min. The mixture was centrifuged at 12,000 rpm at 4°C for 15 min, and the supernatant was transferred into an Eppendorf tube. To precipitate the DNA, 0.5 volumes of isopropanol were added to the supernatant. The mixture was then centrifuged again at 12,000 rpm for 5 min to pellet the DNA, which was then washed with 1.0 ml of 70% ethanol and was resuspended in 50 μl of distilled water. For qPCR analysis, we used SYBR Green (Applied Biosystems) and the same primers used in the qRT-PCR analyses (table S2).

### The HCR RNA-FISH assay

We followed the protocol for the multiplexed in situ HCR as previously described (*40*). There are three subfamilies of *TART* elements (*9*), and our analyses focused on *TART-A*. The following probe sets and amplifiers were obtained from Molecular Instruments: the B1-Alexa Fluor 488 amplifiers were used in conjunction with the probe set designed for *HeT-A* (lot number, PRO329; GenBank, accession #: X68816.1); the B2-Alexa Fluor 594 amplifiers were used with the probe sets designed for *TART-A* (lot number, PRO330; GenBank, AJ566116.1); and the B3-Alexa Fluor 647 amplifiers were applied alongside with the probe sets designed for *TAHRE* (lot number, PRQ334; GenBank, AJ542581.2) and *Jockey* (lot number, RTQ037; GenBank, AY032740.1; probe sets were designed recognizing region of 361-1806 of the *Jockey* element). Confocal images were captured using a Zeiss LSM900 confocal microscope system and processed with Adobe Photoshop 2021. The HCR results were quantified using ImageJ. For the HCR results in salivary gland cells, the relative fluorescence intensity (RFI) was measured across the entire cell with the background noise (the RFI reading from regions out of the cells) subtracted. The quantification of RFI of HCR results in wing discs is illustrated in fig. S2G. Significance levels were determined using one-tailed unpaired *t* tests (*N* > 3 for each experiment).

### Identification of the genome-wide CDK8-, Dp-, and Sd-binding sites through CUT&RUN sequencing

We performed the CUT&RUN assay following the same protocol outlined previously (*93*). We used the CUT&RUN assay kit purchased from Cell Signaling Technology (#86652) using IgG from the kit as the negative control, along with the anti-GFP antibody obtained from Abcam (ab290). The analysis of the CUT&RUN sequencing data was conducted using *Drosophila* genome (assembly Release r5.9) as the reference. The sequencing data from the CUT&RUN assay conducted in this study have been deposited in NCBI’s GEO under the accession number GSE280471. To visualize the gene tracks, we used the Integrative Genomics Viewer (IGV 2.14.1) browser, setting the *y* axis to autoscale for optimal presentation and clarity.

### Peaking calling and motif enrichment analyses

Sequencing reads were aligned to the *D. melanogaster* genome (DMEL 6.45) using Burrows-Wheeler aligner (*94*). Peaks in the resulting coverage files were identified with the “callpeak” command in MACS2 (*95*), and motifs within these peaks were subsequently identified using the “findMotifsGenome” command in Homer (*96*).

### ChIP analyses

ChIP analyses were performed using *Dp^EGFP^* embryos collected for 24 hours, following a published protocol (*97*) with minor adjustments. After fixation and quenching of cross-linking, the chromatin was fragmented to sizes ranging 100 to 300 bp using a Covaris S220 Focused Ultrasonicator. Subsequently, the fragmented chromatin was incubated overnight at 4°C with GFP-Trap coupled to magnetic agarose beads (Bulldog, GTMA-020). After washing, the chromatin was eluted from the beads, and the DNA was decross-linked from the protein. Last, qPCR was performed using the purified DNA as template. The specific primers are listed in table S2, including those for *TART*, as reported previously (*98*).

### Statistical analyses

For each genotype analyzed in this study, we performed a minimum of three independent biological replicates. *P* values were calculated using Microsoft Excel, and SD is represented by error bars in the figures. Significance levels, determined using one-tailed unpaired *t* tests, were denoted as follows: *P < 0.05; **P < 0.01; ***P < 0.001.
