# Supplementary material for: Transcriptional coupling of telomeric retrotransposons with the cell cycle
Source: Sci Adv. 2025 Jan 3;11(1):eadr2299. doi: 10.1126/sciadv.adr2299 (PMC11698117; doi:10.1126/sciadv.adr2299)
Supplement: Supplementary file 1 — Figs. S1 to S20 Tables S1 and S2 [file sciadv.adr2299_sm.pdf]

Science Advances
AAAS

Supplementary Materials for

Transcriptional coupling of telomeric retrotransposons with the cell cycle

Mengmeng Liu *et al.*

Corresponding author: Jun-Yuan Ji, ji@tulane.edu

Sci. Adv. **11**, eadr2299 (2025)
DOI: 10.1126/sciadv.adr2299

This PDF file includes:

Figs. S1 to S20
Tables S1 and S2

Fig. S1.

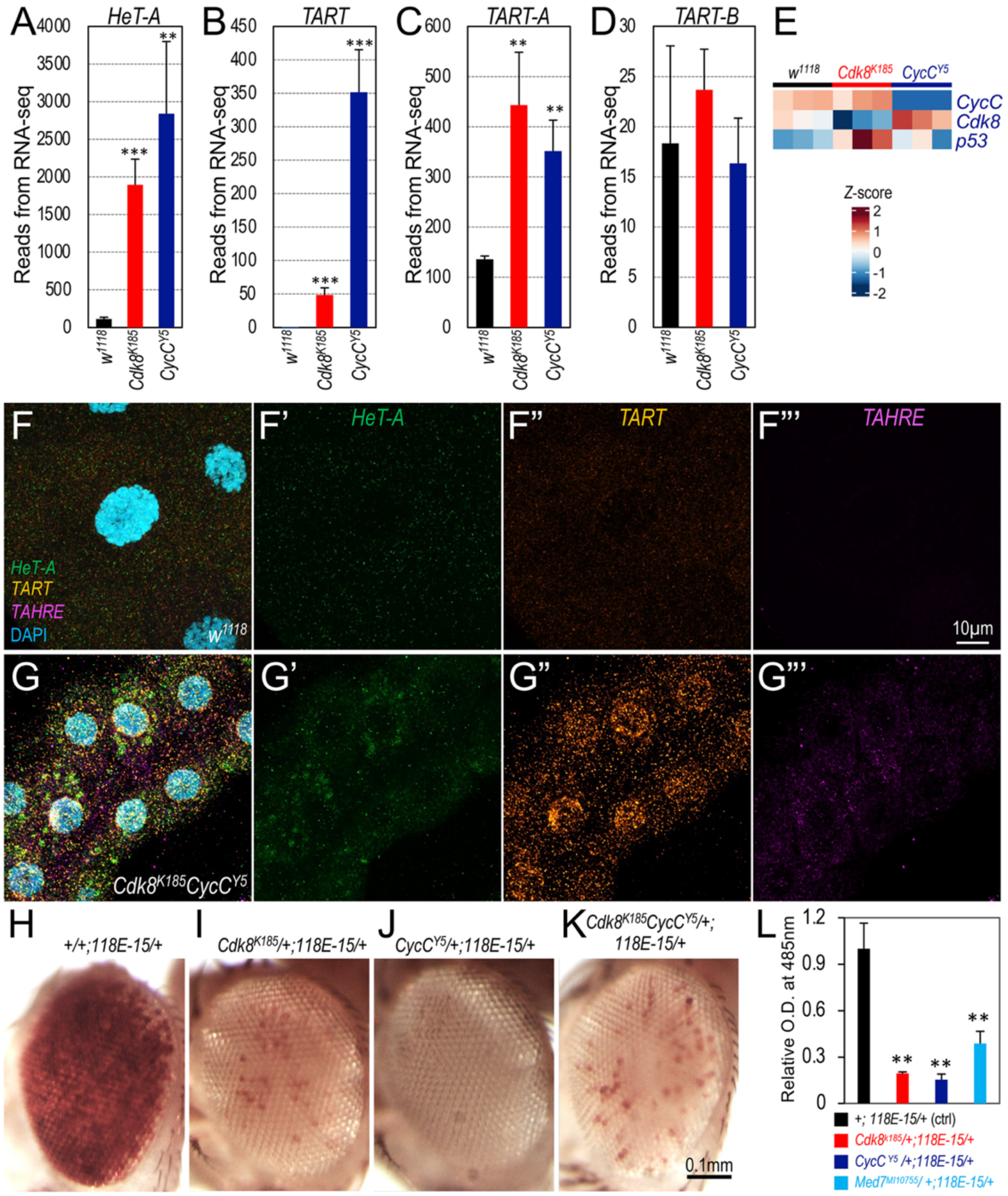

Fig. S1. Effects of *Cdk8* and *CycC* mutations on TR expression and telomere length. (A-D) RNA-seq analysis of transcript levels for *HeT-A* (A), *TART* (B), *TART-A* (C), and *TART-B* (D) in triplicate samples from control (w^{1118}), $Cdk8^{K185}$, and $CycC^{Y5}$ mutant third instar wandering larvae. (E) Heatmap illustrating the expression levels of *CycC*, *Cdk8*, and *p53* in triplicate samples from w^{1118} (control), $Cdk8^{K185}$, and $CycC^{Y5}$ mutant larvae at the same stage. (F, G) HCR RNA-FISH detecting the three TRs in salivary glands of (F) control (w^{1118}) and (G) $Cdk8^{K185}$ $CycC^{Y5}$ double mutants (genotype: '+; +; $Cdk8^{K185}$ $CycC^{Y5}$'). Scale bar in (F'''): 10 µm. (H-K) Dominant enhancement of telomeric position variegation assessed via mottled eye color phenotypes in male adult flies (*118E-15*). Genotypes: (H) w^{1118}/Y; +*;* +*; 118E-15/*+; (I) +/Y; +*;* $Cdk8^{K185}$/+*; 118E-15/*+; (J) +/Y; +*;* $CycC^{Y5}$/+*; 118E-15/*+; and (K) +/Y; +*;* $Cdk8^{K185}$ $CycC^{Y5}$/+*; 118E-15/*+. Scale bar in (K): 0.1 mm. (L) Quantification of red pigment in adult fly heads, measured at O.D. 485nm. Genotypes are color-coded for clarity in the chart below the figure.

Fig. S2.

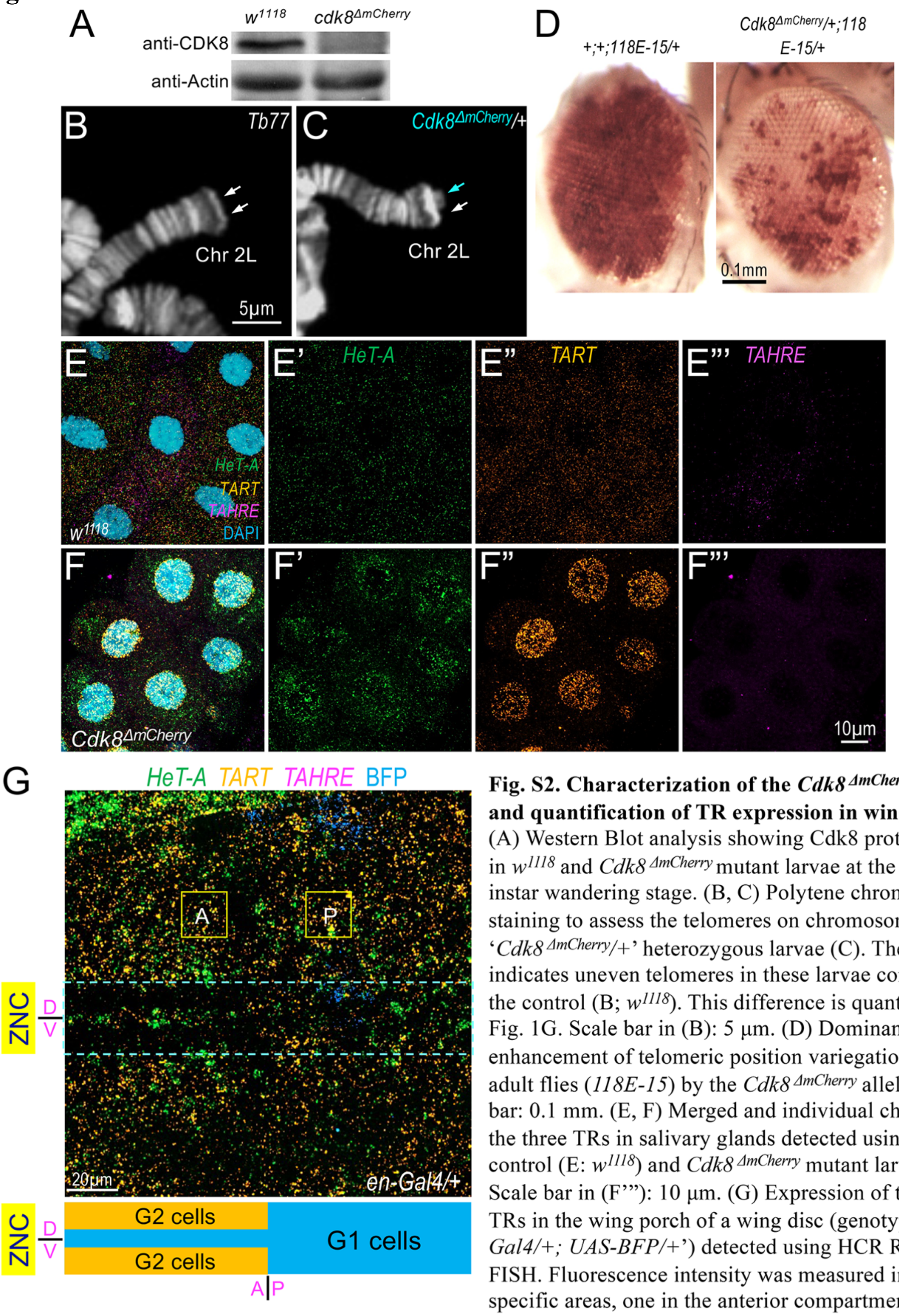

Fig. S2. Characterization of the *Cdk8* $^{\Delta mCherry}$ allele and quantification of TR expression in wing discs. (A) Western Blot analysis showing Cdk8 protein levels in w^{1118} and *Cdk8* $^{\Delta mCherry}$ mutant larvae at the third instar wandering stage. (B, C) Polytene chromosome staining to assess the telomeres on chromosome 2L in '*Cdk8* $^{\Delta mCherry}$/+' heterozygous larvae (C). The staining indicates uneven telomeres in these larvae compared to the control (B; w^{1118}). This difference is quantified in Fig. 1G. Scale bar in (B): 5 µm. (D) Dominant enhancement of telomeric position variegation in male adult flies (*118E-15*) by the *Cdk8* $^{\Delta mCherry}$ allele. Scale bar: 0.1 mm. (E, F) Merged and individual channels of the three TRs in salivary glands detected using HCR in control (E: w^{1118}) and *Cdk8* $^{\Delta mCherry}$ mutant larvae (F). Scale bar in (F'''): 10 µm. (G) Expression of the three TRs in the wing porch of a wing disc (genotype: '*en-Gal4/+; UAS-BFP/+*') detected using HCR RNA-FISH. Fluorescence intensity was measured in two specific areas, one in the anterior compartment (labeled as 'A') and another in the posterior compartment ('P'). ImageJ was used to quantify the fluorescence intensities and calculate the posterior-to-anterior (P/A) ratio. Notably, in the Zone of Nonproliferating Cells (ZNC) of the wing disc, TR expression is lower in cells in the G2 phase compared to those in the G1 phase. Scale bar: 20 µm.

Fig. S3.

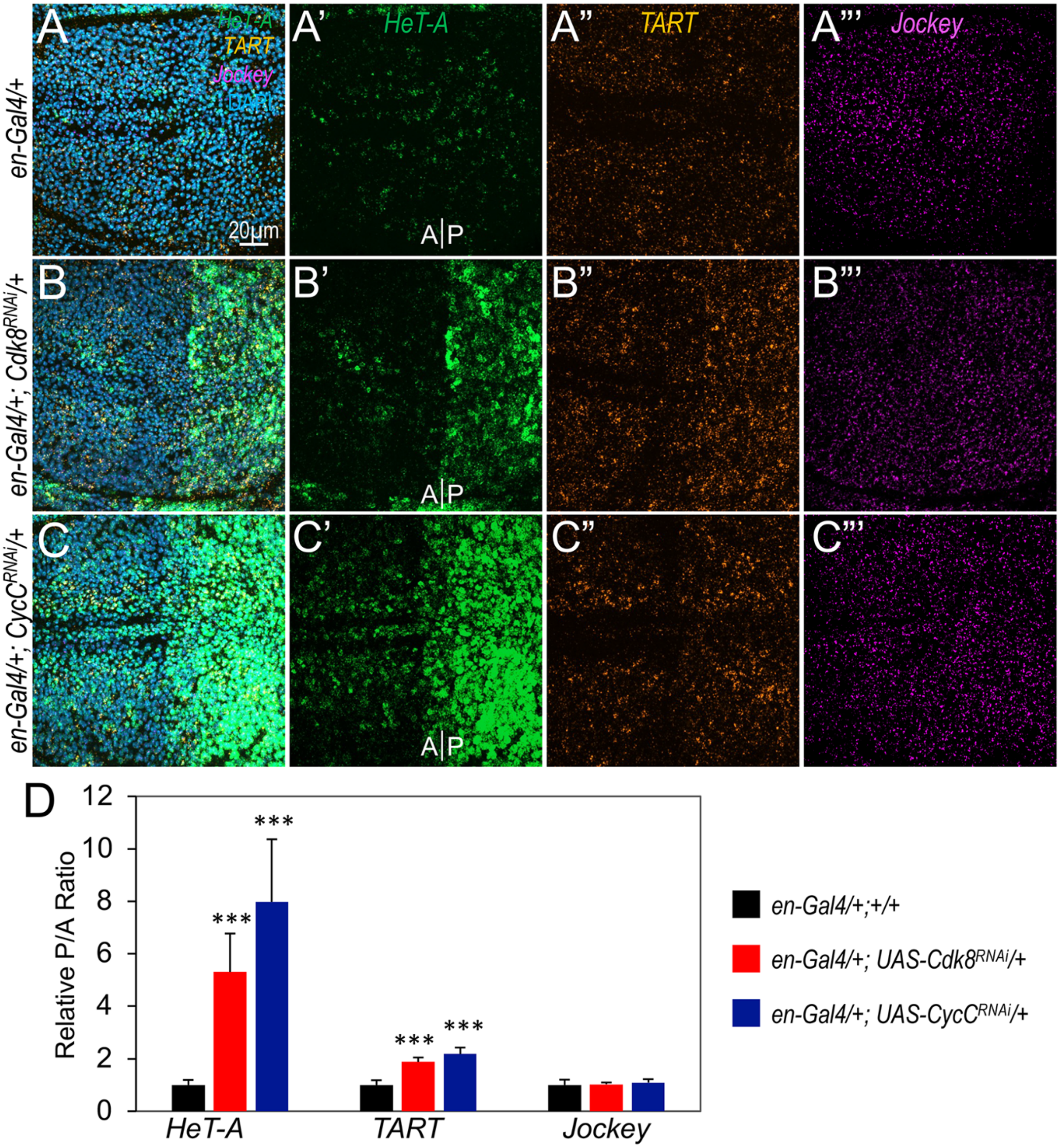

Fig. S3. Depletion of *Cdk8* or *CycC* increases the expression of *HeT-A* and *TART*, but not *Jockey*. Detection of mRNA transcripts for *HeT-A* (green), *TART* (orange), and *Jockey* (magenta) using HCR RNA-FISH assay in wing discs of the following genotypes: (A) '*en-Gal4/+; +*'; (B) '*en-Gal4/+; UAS-Cdk8^{RNAi}/+*'; and (C) '*en-Gal4/+; UAS-CycC^{RNAi}/+*'. Scale bar in (A): 20 µm. (D) Posterior-to-anterior (P/A) fluorescence intensity ratio calculated for each genotype (3-5 discs per group). Statistical significance was assessed using one-tailed unpaired *t*-tests, with significance indicated as follows: *** p<0.001.

Fig. S4

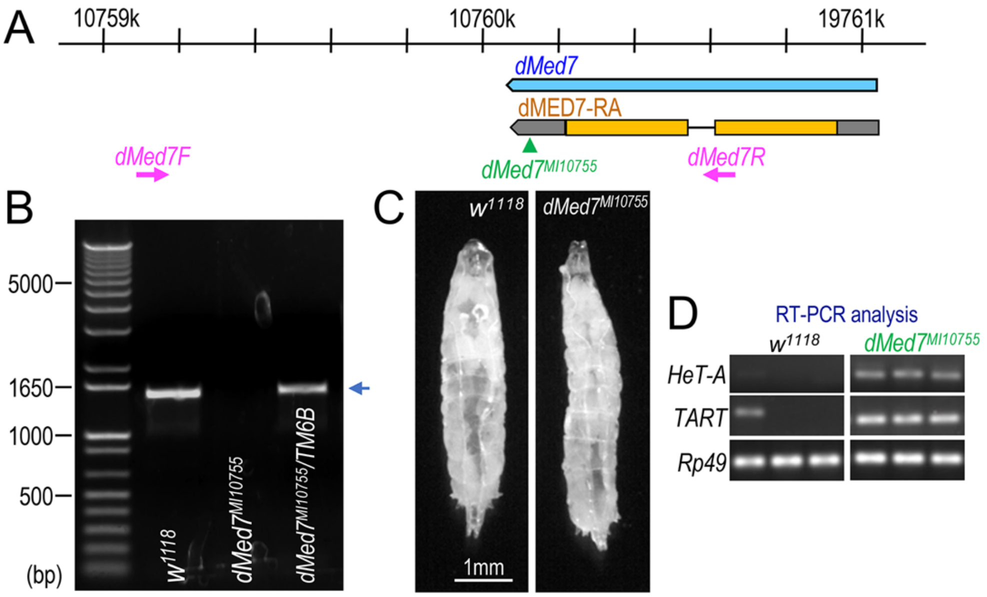

Fig. S4. Characterization of the *dMed7^{MI10755}* mutant allele. (A) Schematic representation of the *dMed7* genomic locus, with the green arrow indicating the insertion site of the *dMed7^{MI10755}* allele. (B) PCR validation of the *dMed7^{MI10755}* allele using primers (*dMed7F* and *dMed7R*) shown in (A). No PCR products were observed in genomic DNA from *dMed7^{MI10755}* homozygous mutant larvae, confirming the presence of the transposon insertion. (C) Homozygous *dMed7^{MI10755}* mutants exhibit developmental arrest at the third instar larval stage. (D) qRT-PCR analysis demonstrated upregulation of *HeT-A* and *TART* transcripts in *dMed7^{MI10755}* mutant larvae. However, the transcript levels of *TAHRE* were too low to be detected in this assay.

Fig. S5.

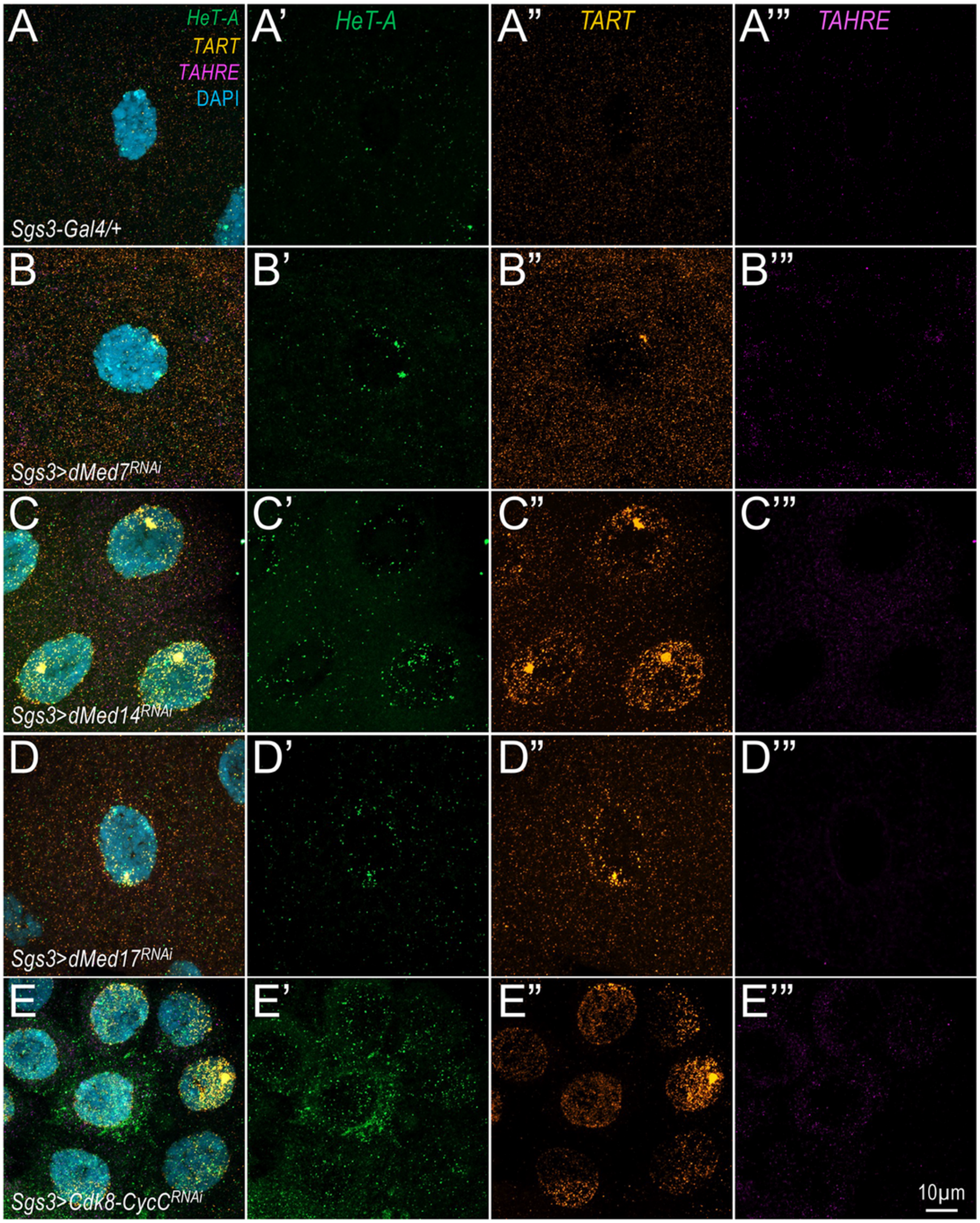

Fig. S5. Detection of the three TRs in salivary glands using the HCR RNA-FISH assay: merged and individual channels. *HeT-A* (green; A'-E'), *TART* (orange; A''-E''), *TAHRE* (magenta; A'''-E'''), and DAPI (blue) staining in salivary gland cells. The analyzed genotypes are: (A) *Sgs3-Gal4/+; +* (control); (B) *Sgs3-Gal4/+; UAS-dMed7*^{RNAi}/+; (C) *Sgs3-Gal4/+; UAS-dMed14*^{RNAi}/+; (D) and *Sgs3-Gal4/+; UAS-dMed17*^{RNAi}/+; and (E) *Sgs3-Gal4/+; UAS-Cdk8*^{RNAi} *CycC*^{RNAi}/+. Scale bar in (E'''): 10 µm.

Fig. S6.

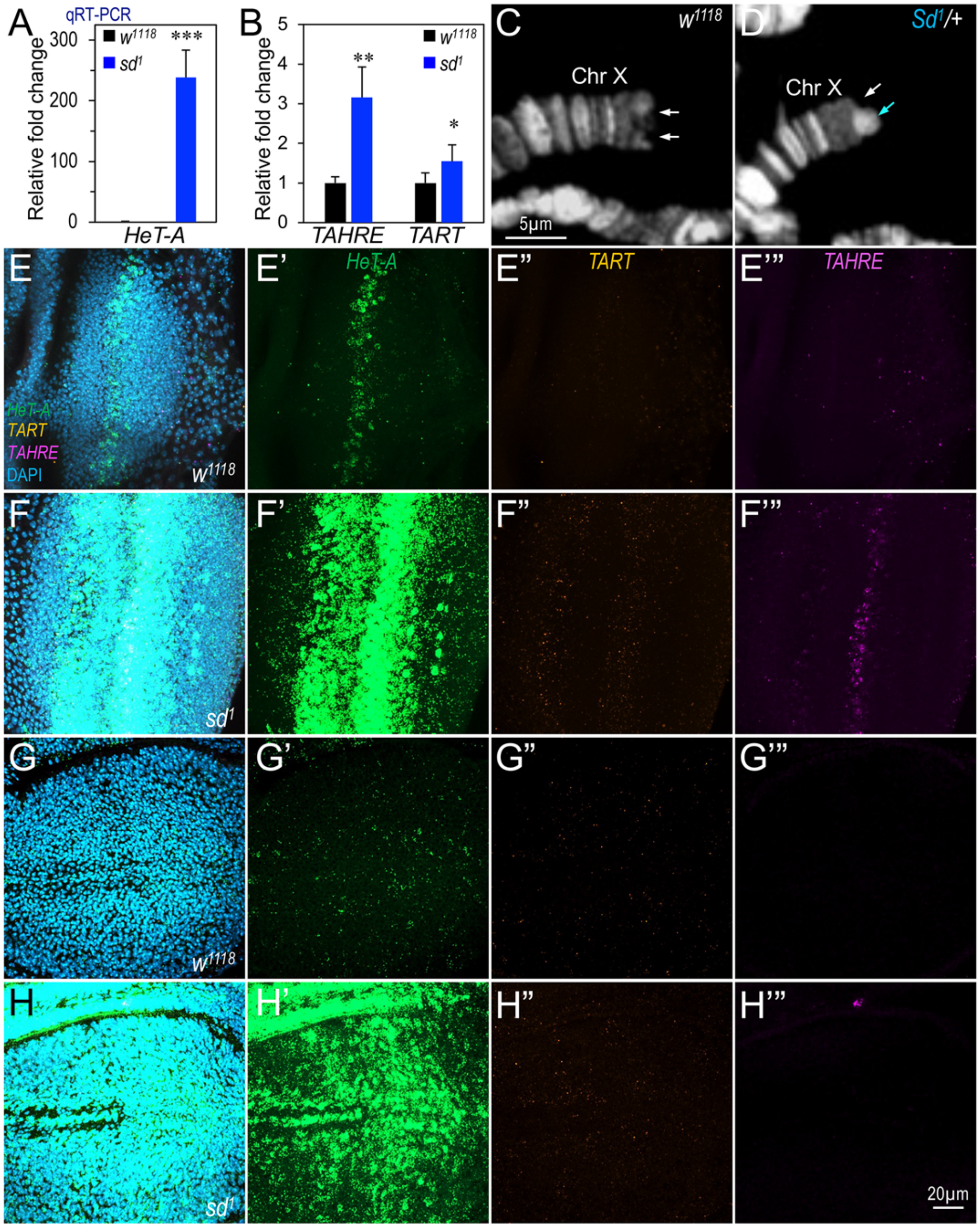

Fig. S6. Further analysis of the effect of the *sd* mutation on TR expression. (A, B) qRT-PCR analysis showing transcript levels of TRs in w^{1118} (control) and sd^1 homozygous mutant larvae. (C, D) Polytene chromosome staining to assess telomere ends in polytene chromosomes from (C) w^{1118} (control) and (D) 'sd^1/+' heterozygous mutant larvae. Uneven telomere ends in sd^1/+ heterozygous mutants are indicated by arrows. Quantification of this phenotype is shown in Fig. 1G. Scale bar in (C): 5 µm. (E-H) Detection of *HeT-A* (green; E'-H'), *TART* (orange; E"-H"), and *TAHRE* (magenta; E"'-H"') mRNA transcripts using the HCR RNA-FISH assay in eye discs (E, F) and wing discs (G, H) from third instar larvae at the wandering stage. Nuclei were stained with DAPI (blue). Eye discs: elevated TR expression along the morphogenetic furrow in sd^1 mutants (F'-F"') compared to the control (w^{1118}, E'-E"'). Wing discs: Significant upregulation of *HeT-A* was detected in sd^1 mutants (H') compared to the control (w^{1118}, G'). Scale bar in (H"'): 20 µm.

Fig. S7.

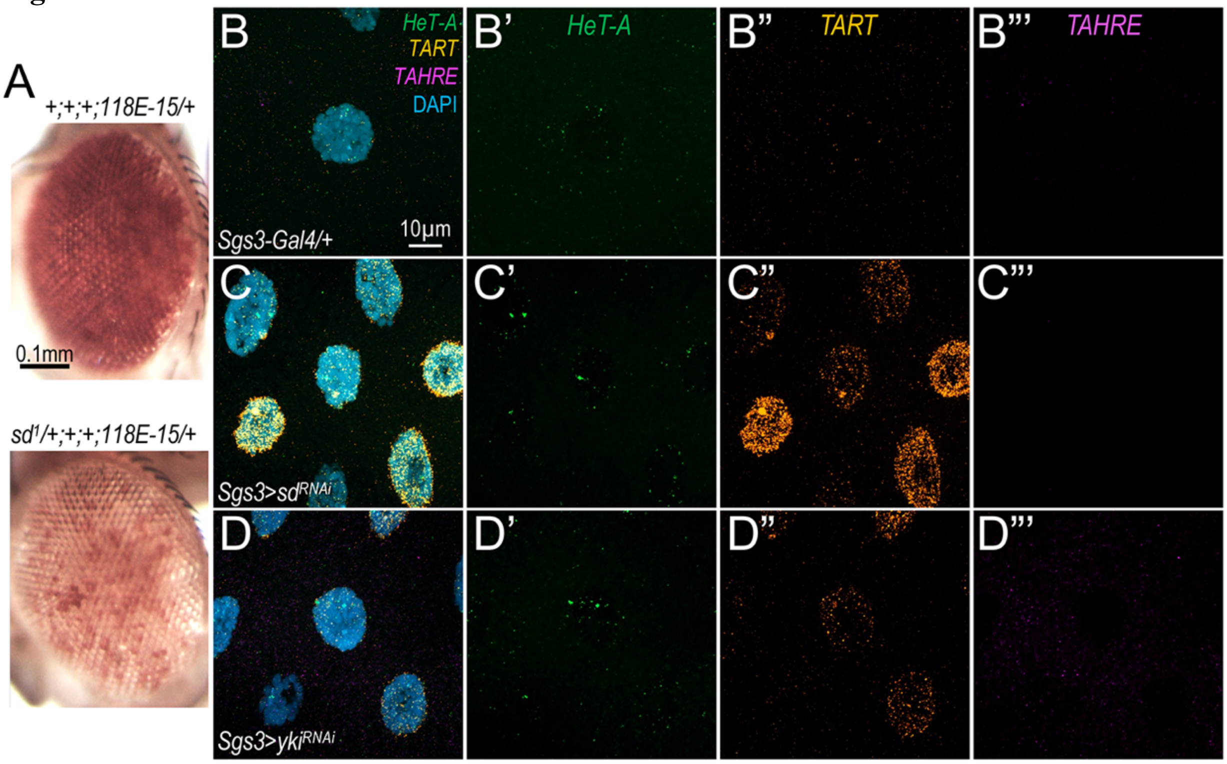

Fig. S7. Effect of Sd and Yki on TR expression and telomere homeostasis. (A) Dominant enhancement of the telomeric position variegation observed in *118E-15* female adult flies. The mottled eye color phenotype in the control genotype ('w^{1118}; +; +; *118E-15/+*') was enhanced by the presence of the sd^{1} mutant allele (genotype: 'sd^{1}/w^{1118}; +; +; *118E-15/+*'). Scale bar: 0.1 mm. (B-D) Detection of the three TRs using HCR RNA-FISH in salivary glands, showing merged and individual channels: (B) *Sgs3-Gal4/+; +* (control); (C) *Sgs3-Gal4/+; UAS-*sd^{RNAi}/+; and (D) *Sgs3-Gal4/+; UAS-*yki^{RNAi}/+. Scale bar in (B): 10 µm.

Fig. S8.

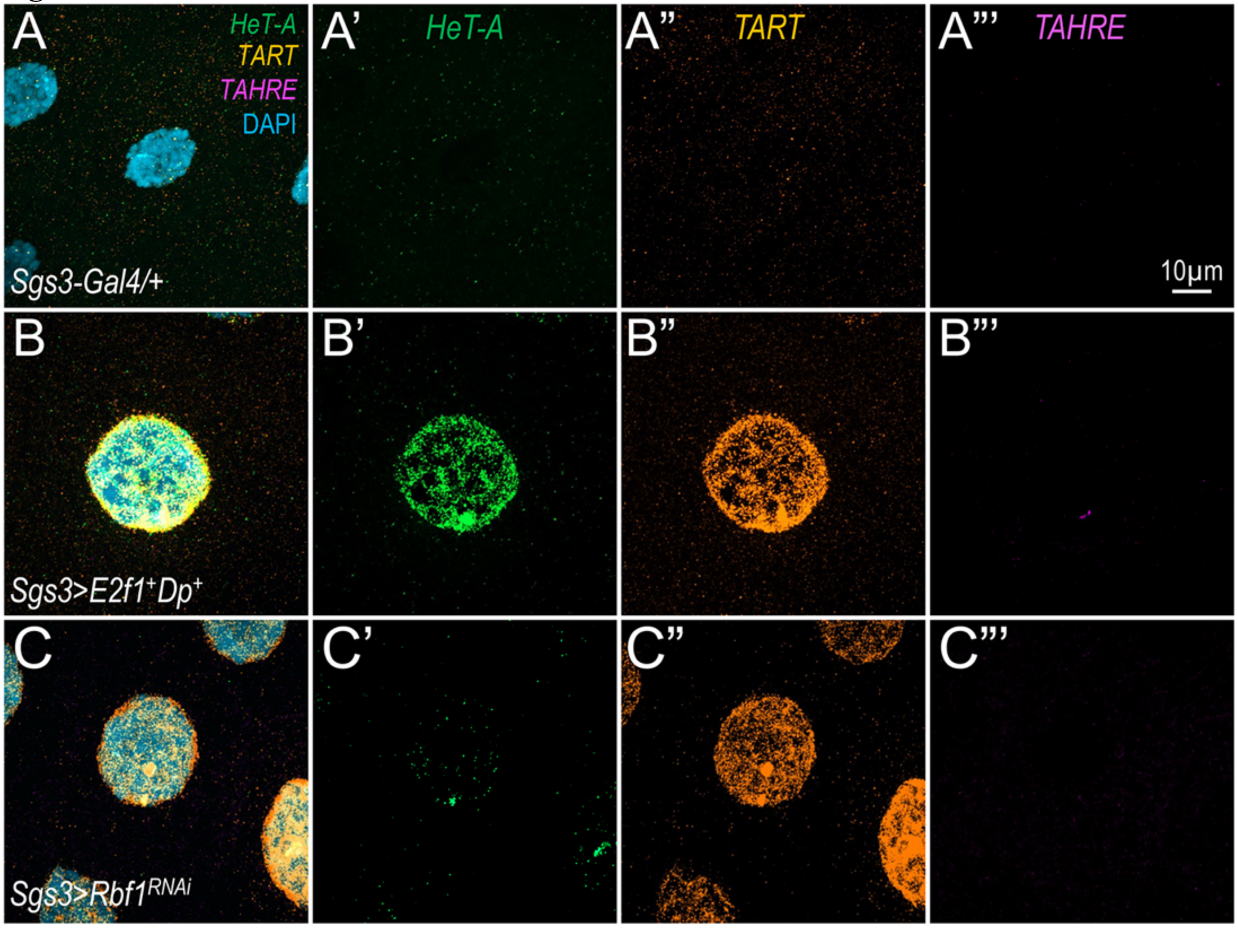

Fig. S8. Merged and individual channels of the three TRs in salivary glands detected using HCR RNA-FISH assay. Detection of *HeT-A* (green; A'-C'), *TART* (orange; A''-C''), *TAHRE* (magenta; A'''-C'''), and DAPI (blue) in salivary glands of the following genotypes: (A) *Sgs3-Gal4/+; +* (control); (B) *Sgs3-Gal4/+; UAS-E2f1^{+} UAS-Dp^{+}/+*; and (C) *Sgs3-Gal4/+; UAS-Rbf1^{RNAi}/+*. Scale bar in (A'''): 10 µm.

Fig. S9.

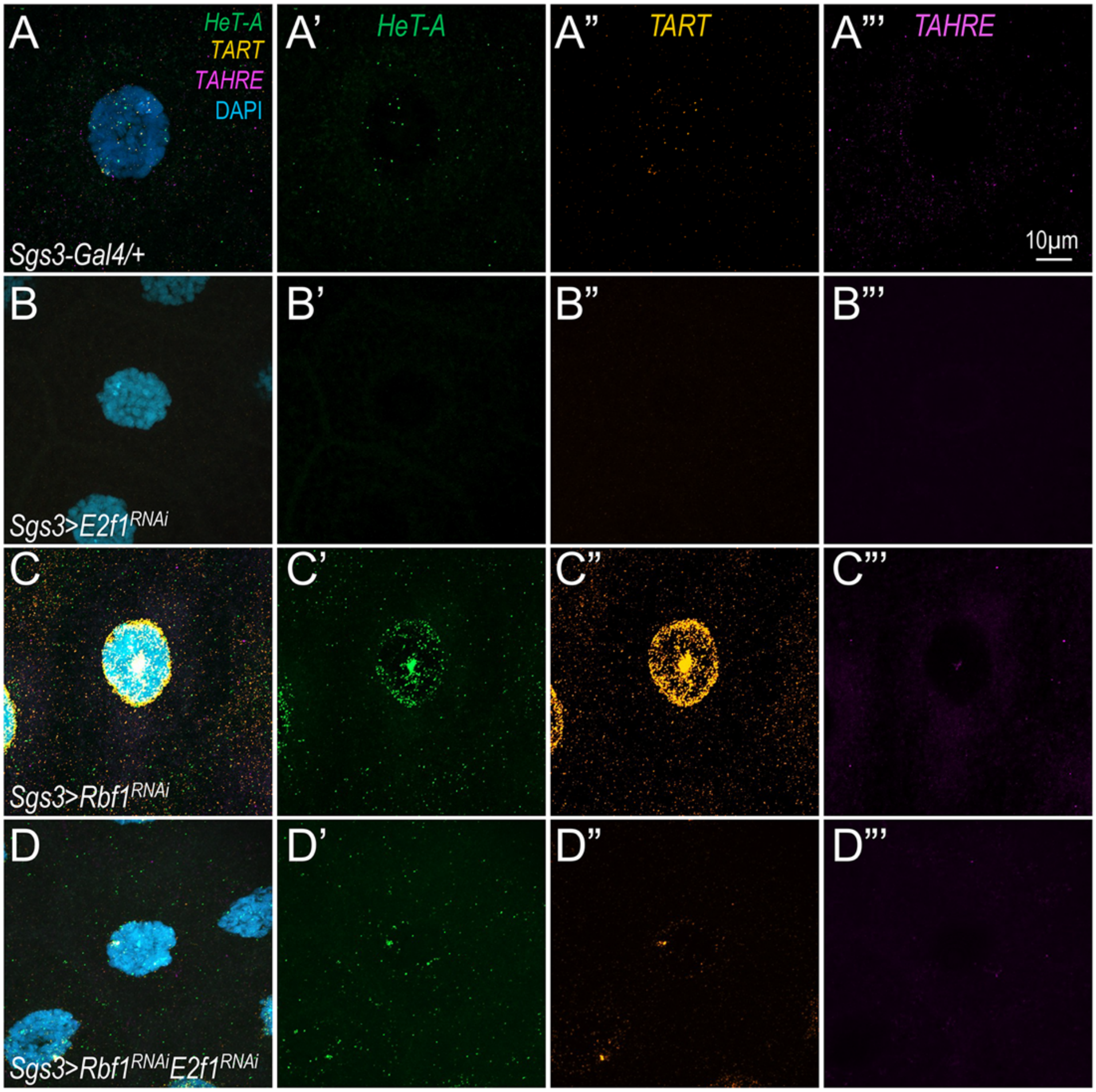

Fig. S9. Merged and individual channels of the three TRs in salivary glands detected using HCR RNA-FISH assay. Detection of *HeT-A* (green; A'-D'), *TART* (orange; A"-D"), *TAHRE* (magenta; A"'-D"'), and DAPI (blue) in salivary glands of the following genotypes: (A) *Sgs3-Gal4/+; +* (control); (B) *Sgs3-Gal4/+; UAS-E2f1*^{RNAi}/+; (C) *Sgs3-Gal4/+; UAS-Rbf1*^{RNAi}/+; (D) *Sgs3-Gal4/+; UAS-Rbf1*^{RNAi}*/UAS-E2f1*^{RNAi}. Scale bar in (A"'): 10 µm.

Fig. S10.

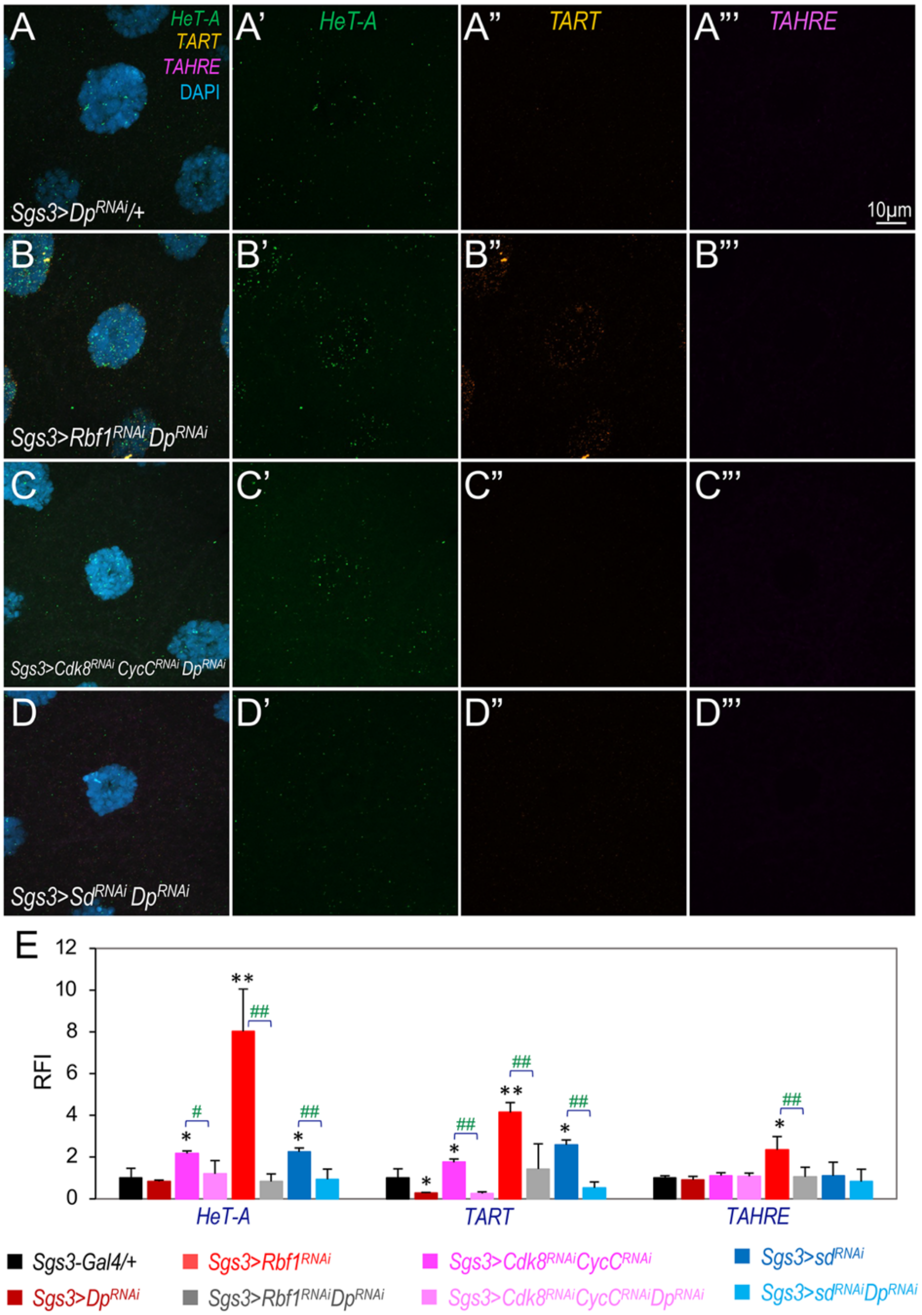

Fig. S10. Dependency of TR expression on Dp following depletion of Rbf1, Cdk8-CycC, and Sd/dTEAD. (A-D) Expression of *HeT-A* (green; A'-D'), *TART* (orange; A"-D"), and *TAHRE* (magenta; A"'-D"') detected in salivary glands using the HCR RNA-FISH assay for the following genotypes: (A) *Sgs3-Gal4/+; UAS-Dp*^{RNAi}/+; (B) *Sgs3-Gal4/+; UAS-Rbf1*^{RNAi}*/UAS-Dp*^{RNAi}; (C) *Sgs3-Gal4/+; UAS-Cdk8*^{RNAi} *CycC*^{RNAi}*/UAS-Dp*^{RNAi}; and (D) *Sgs3-Gal4/+; UAS-sd*^{RNAi}*/UAS-Dp*^{RNAi}. Additional controls are provided in Fig. 6. Scale bar in (A"'): 10 µm. (E) Quantification of relative fluorescence intensity (RFI) for the TR transcripts, with specific genotypes color-coded. * indicates comparisons with the control genotype ('*Sgs3-Gal4/+; +*'), and # indicates comparisons with the corresponding controls, as specified in the chart. Statistical significance: * or # $p<0.05$, ** or ## $p<0.01$ (one-tailed unpaired *t*-tests).

Fig. S11.

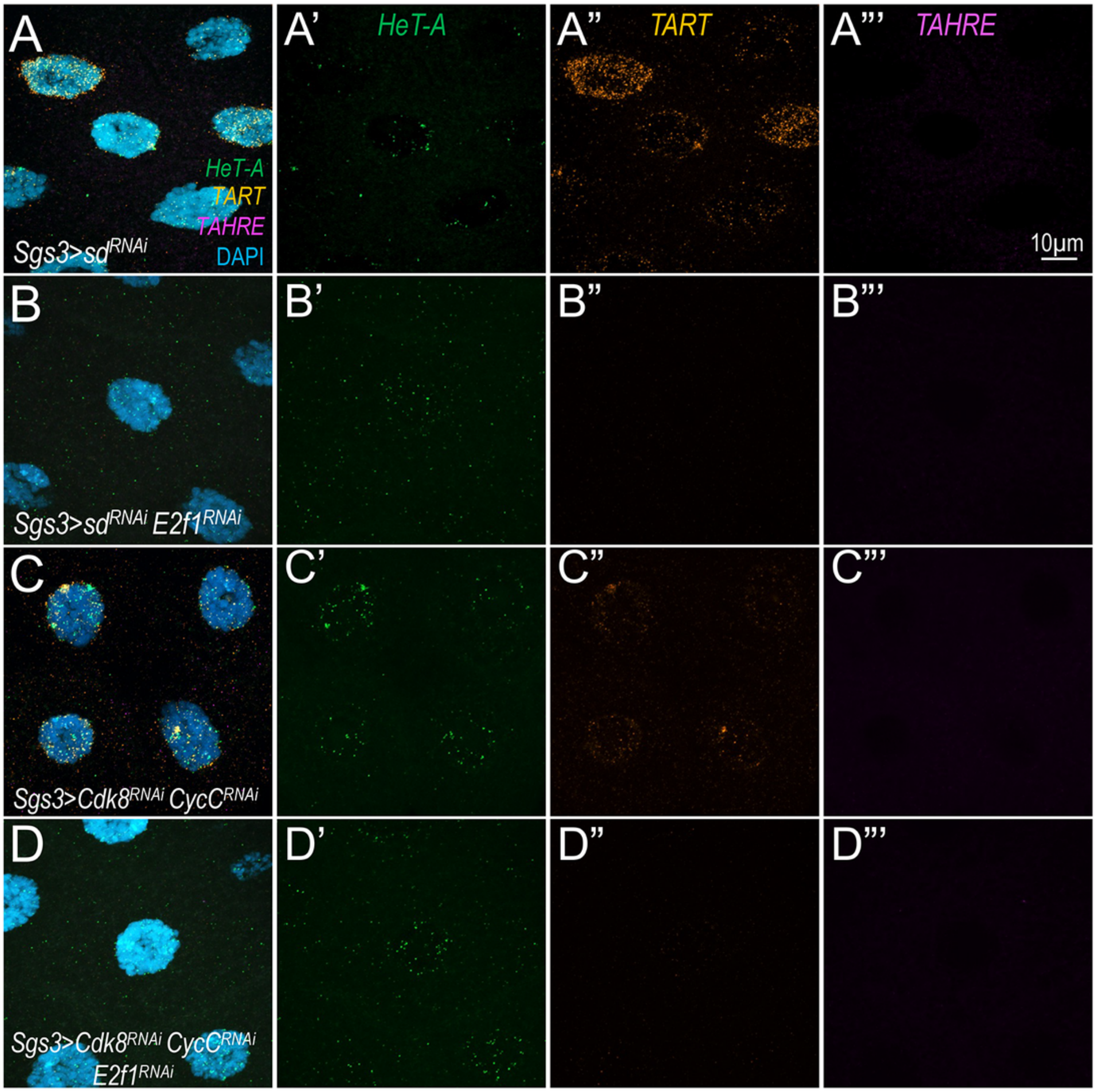

Fig. S11. Merged and individual channels of the three TRs in salivary glands detected using HCR RNA-FISH assay. Detection of *HeT-A* (green; A'-D'), *TART* (orange; A"-D"), *TAHRE* (magenta; A"'-D"'), and DAPI (blue) in salivary glands for the following genotypes: (A) *Sgs3-Gal4/+; UAS-sd^{RNAi}/+*; (B) *Sgs3-Gal4/+; UAS-sd^{RNAi}/UAS-E2f1^{RNAi}*; (C) *Sgs3-Gal4/+; UAS-Cdk8^{RNAi} CycC^{RNAi}/+*; and (D) *Sgs3-Gal4/+; UAS-Cdk8^{RNAi} CycC^{RNAi}/UAS-E2f1^{RNAi}*. Scale bar in (A"'): 10 μm.

Fig. S12.

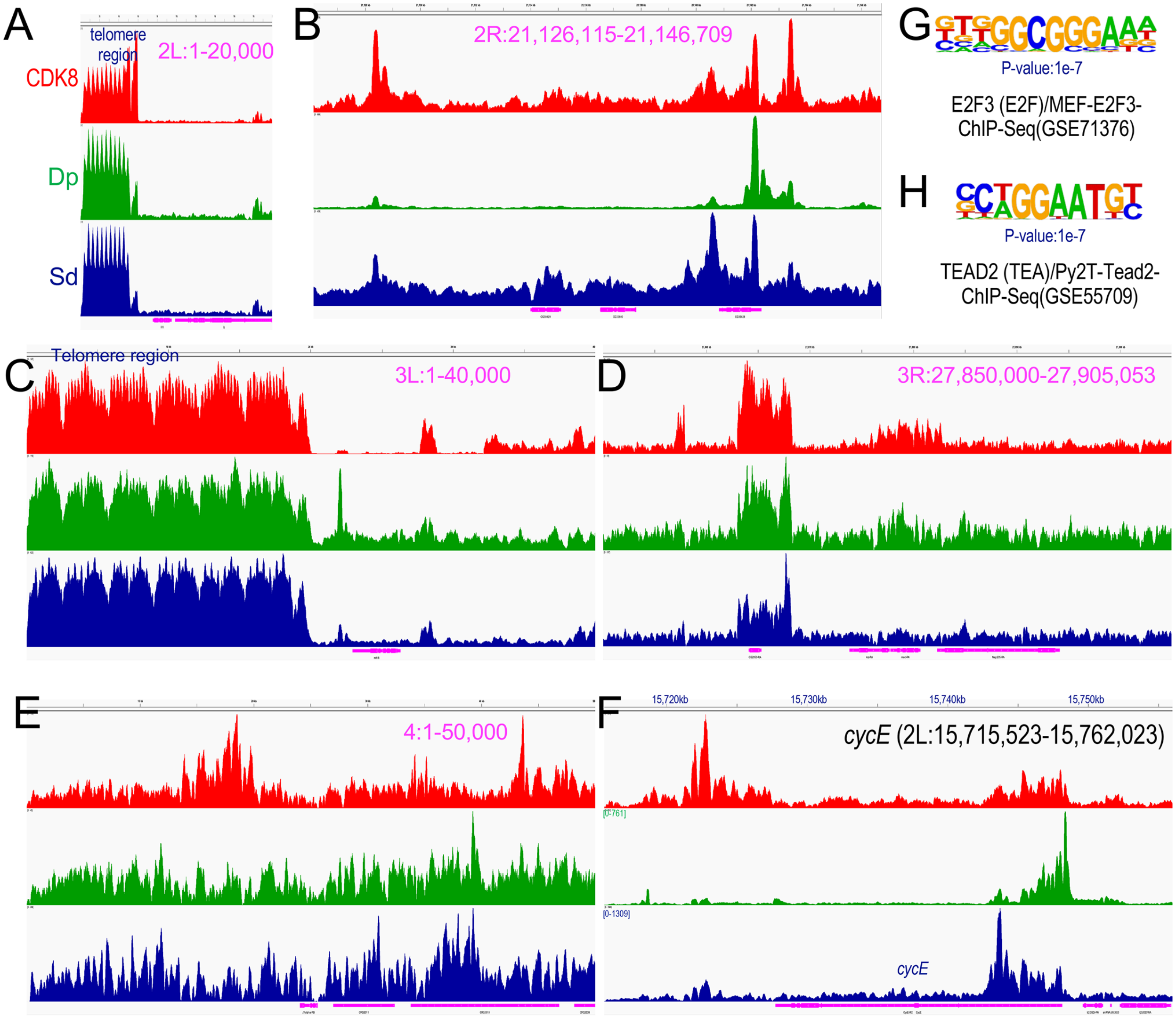

Fig. S12. Additional analyses of CUT&RUN data. Genomic tracks displaying binding peaks for Cdk8 (red), Dp (green), and Sd (dark blue) at telomeric regions of different *Drosophila* chromosomes: (A) Telomere of 2L (left end of chromosome); (B) Telomere of 2R (right end of chromosome), (C) Telomere of 3L, (D) Telomere of 3R, (E) Telomere of 4L, and (F) *cyclin E* locus. These telomeric regions have limited annotation in the *Drosophila* genome (assembly Release dmel_r5.9 or dm6). (G, H) Identification of additional top-matching consensus motifs using Homer Known Motif Enrichment analysis (50bp window): (G) 'E2F3 (E2F)/MEF-E2F3-ChIP-Seq (GSE71376)', enriched for Dp; (H) 'TEAD2 (TEA)/Py2T-Tead2-ChIP-Seq (GSE55709)', enriched for Sd/dTEAD.

Fig. S13.

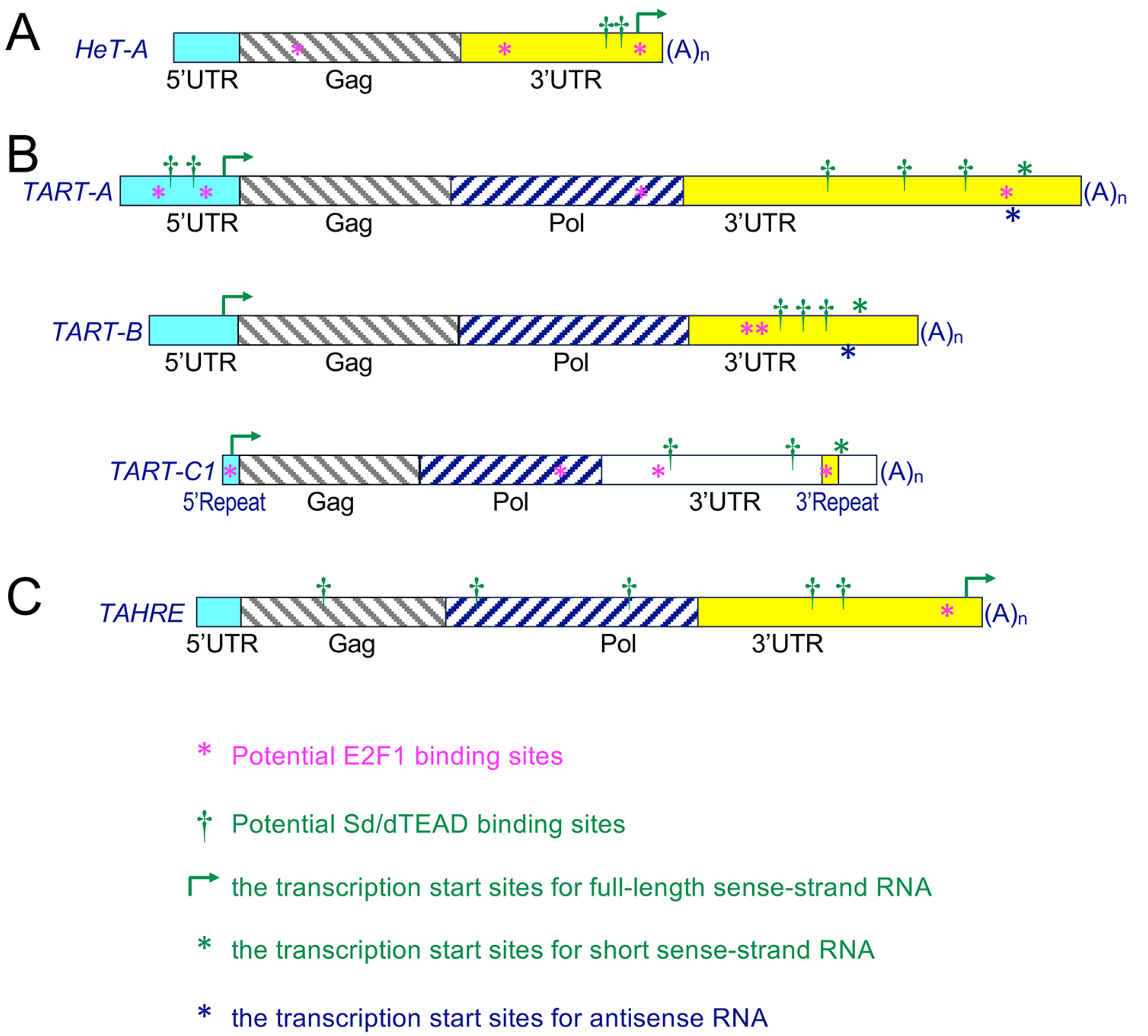

Fig. S13. Identification of potential E2F1- and Sd/dTEAD-binding sites in TRs. Magenta asterisks (*) indicate potential E2F-binding sites, while green daggers (†) mark potential Sd/dTEAD-binding sites. The 5' untranslated region (5'UTR) is shown in light blue; the 3'UTR is in yellow; green bent arrows represent transcription start sites (TSS) for full-length sense-strand RNA; green asterisks above *TART-A/B/C1* elements denote the TSS for short sense-strand RNA, while dark blue asterisks below *TART-A/B* indicate the TSS for antisense RNA. This structural overview of the TRs is adapted from reference (*2*) and the GenBank annotation (see Suppl. Figs. S14-S19). (A) *HeT-A*: contains two potential E2F1-binding sites and two potential Sd/dTEAD-binding sites. Additionally, one potential E2F1-binding site is located within the sequence encoding the Gag protein. (B) *TART-A*, *TART-B*, and *TART-C*: potential E2F1- and Sd/dTEAD-binding sites are identified in the 5'UTR, 3'UTR, or within the sequence encoding the Pol protein. (C) *TAHRE*: contains one potential E2F1-binding site in the 3'UTR; multiple potential Sd/dTEAD-binding sites are located in the 3'UTR and within sequences encoding the Gag and Pol proteins.

Fig. S14.

Drosophila melanogaster **retrotransposon HeT-A subfamily D, clone01D09**

GenBank: AJ635224.1

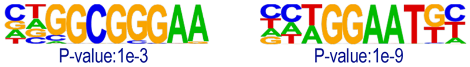

E2F1(E2F)/Hela-E2F1-ChIP-Seq(GSE22478)

TEAD(TEA)/Fibroblast-PU.1-ChIP-Seq

3'UTR (1..427)
5'UTR (428..1120)
Gag (1121..3976)
Reversed TEAD binding site: ATTCC
E2F1 site: GCGGGA
3'UTR (3977..>5743)

TT[C/G][C/G]CGC motifs:
TTCCCGC or TTCGCGC or TTGGCGC

>AJ635224.1 *Drosophila melanogaster* retrotransposon HeT-A subfamily D, clone01D09

GCGGCCGCAACAGACCACAGCCATCATACAACAAACACATACCTGTGACTACTCCTGAGGTTCCCAGCGACTCGGTGCTTCCGTCCTTCTGGAGGGGGTACCTAAAA
AGAATCAATCAAGCAATGTTAGTCTTAAATATCAATGTCTTTGGTAAAATAATTTTATTTATAAAATGTAAACACAACATACAATGTGATAATGTTACCAGTCCATG
TTACTGTCGAAAACCCAATGTTTACAAAAATACTTACTTTTAATATTAATACTAAATCTATGTCCAATCCCCAAACTCACCCCACGCAATGTACTCTCAAAAATTTA
AATTATTGTACCTACATATTGCATACTATGTAATCAAAGGCAAAATAAATTGTGGATGCGGAACAGAACTCATTCTGTCTCCGTACCTCCACCAGCAAAGTTAAAAT
TAACCAGCAAAGTTAAAAACGCGCAAATATAATTTAAATCGCCTTTTCATTTTGACAAATTAAAAGTTTAAATATTGTCTTCCGGCCGCAAAGTTAATACCGCGACA
ATAAAAAACAATTAATCGACAAACGAAAAGCGAACAGTTATACTTGTGCAAAATCGACAAGCTGCCGTTATAACAAAAAGGAGACGAAGAACCAAAGATTAAAGTTA
AATCCACCAGCAAAGTTAAAGTTCCAACCAAACAATAAAACAAATTAAATAAATAAATTAAACTAATTAAATAAACTAGTAAATTGAACCTATTTAACAAAATAATT
CGCGCTTCCACCTGCATAATTTTCTCACGCGCAATTTAATTTTAAATCGCCTTTTCGTACGAGAAATTAAAGTTTAAAATTGTTTTCCGGACGCAAAGTTAAAACCG
CGACAACAATAAACATTTAGTGGACAAACGAAAGGTGAACAATTAATTGTGTAATAATTGTGCAAATTGACAAGCTGCCGCCATAAAAAGAAAATCCATAGAGACGA
AAAGAAGAAAGACGACCGCAACAAGGAAAATTTAAAGGAAGGAGAGGAGAAAGGCCAATAAAGGAGACGAACTAAACGAGAGGAGACGCCACAAATTAAAAGCCAGG
GTATTTATACCTAAAAGTATCATTTATTACATTAAAAATTAGTAAATACC**ATG**TCTGACCACCACCTATTTACTGATTCCTCTTCTGACGGCGAAATACACTCAATT
ACATCAAGCCCAGAACAGCGAAATTCCCCATTCCACCTTGAAATATCGCCCATGTCCCACGGATCTAATAATTCTCAGTCTAATACAAGCATTATTAGTCTGAAGAA
ATTGCCCTTACAACCAGCAAATAAAAGTTCAAAAAACTCTTCTGGGGCTGCCATAAAAAATTGTTAATTCCCTTTCACATAAGGGGAAAGAGAATTCAAACACTAATA
ATGCCCAAAACGACCCCCTCTCACTCACCAATACTGCTGCAGTCACCAAAGGCGCCAAAAGCAGCATCTCGAAGGGGAAATTGCCTTCTCCTCTATCCACCTCATAC
ACATTTAAGGGGAAATTACACCCAAAAATGACTCACACAAATGCAACTGCACTCACTCATACCGACGCTTCTCAAAGAGAGAAAATACCCACCCCAGCGACTTGCAC
TAACGCAGCTGCAGCAACTAATACGGACACAGATGTAAAAGGCGCCAAACTGAGCGATGCCATGGGAAATTTCCCCTCTCTTTCACACAGCGATAGCATGGACAAAA
ACCTCAGCTCATCCACCAAAATTGGACCCAATGCTCTTTCCCCTCCTACTTACACACACACACAAACAAGCAAAGCAACTTACACAAACTTAGAAGGCTGCTCTAAA
A**TTCCCGC**GCTTGCCAATTCTGACGCACATTACAAGAATGCCAATGTTAGTAACAGTGGGGAAATCTTCTCCCCACAAATACAAATCGACGAACACAAACAAGAGGA
AAGGCCGAGCACAACTCTCAATGCTTTTTGGTCAATTTTCAACCCCAAGCCGGACATTTCTAAACTTAGTCTAAACAAGAAACCCGCCAACCGCTCTGGAAATACTG
GGAAAAGAAGCATTTCCCCACATCAAAGGAATGCTTCCTTACGCTCTTCTGCTCAGGTTGATTCAAATTTAAATCCCAACACTAGCGCCATGCCCACTGTGGGGAAT
TTACCAGCAGCCCGCACACTTAGCCGGCCTGCTGCAAAATTTATTTAATTCATCCTCCAAGAGCCCAAACGAGCATCCTATGAGTTTTTCGGAAGTGGTAGCTGGAA
CAGGTCCAGTTTCTGTGGCACCCTCTAATCCGGCACCACTGACGAAAACTCCAGGCAAGAGGACAAATGGCGAACTGGACTCCTCCAGCTTTAAGACTCCCAACAAA
CGACTATGCGCTACATCAAACTTTAAAACTCCCAGCATTTTTCCTCCTCTTATCACACCTGTTTTTAAAAGCAAGGCAGCTCAATCTGTTTATGAGGAATCAAAAGC
CAGAAATGGACCCCCCGCCAGCCGTTACCCTGCAGCACCAATGCCTCTGCTCGCAGCGCAGCGGCGCCACCCGGGATTGCCCCCCTACCCCCTCAAAATACAGATG
CAGAGCTGCCACCATGGAAAATCTGTGCCCAAGAGCCGTAGAGCACCCCCTATCCTAGTCAACGACGTGAAGGAAATTGTCCCGCTGCTGGAAAAGCTGAACTACACA
GCAGGAGTATCCAGCTACACCACCAGGGCAATAGATGGGAATGGGGTCAGGATCCAGGCCAAGGATATGACTGCCTTCAACAAAATTAAAGAAGTCCTGGTGGCAAA
CGGTTTTCCATTATTCACCAATCAGCCCAAATCTGAGAGAGGATTCCGAGTCGTCATCAGACACCTCCATCATTCCACACCATGTTCGTGGATAGTCGAGGAGCTGC
TGAAGCTCGGATTCCAAGCACGCTTCGTTAGAAATATGACGAATCCAGCTACAGGTGGCCCCATGCGAATGTTTGAAGTTGAGATAGCCATGGCCAAAGACGGCAGT
CACGACAAAATAATCTCACTCAAACAAATCGGTGGGCAAAGAGTGGACATTGAAAGGAAAAACAGGACACGGGAGCCGGTCCAGTGCTACAGATGCCAGGGCTTCAG
GCATGCCAAAAACTCTTGCATGAAGCCGCCACGATGCATGAAATGCGCTGGCGATCACCTGTCATCCTGTTGCACCAAGCCAAGATCCACCCCTGCCACCTGCGTCA
ACTGCTCTGGGGAGCATATTAGTGCATACAAGGGATGCCCTACCTACAAGGCCGAAAACAAAAGCTGGCGGCAAACAACATAAACCTAAACAAAATAAGAACATTC
AAGAACGCAACAAATAACTATTATAGACGTCAAGGCCCCCCTCCACACAATAACACCCCTCGGCTGCCACATAGCTCAGCAATCCTGAGCAAATCAATCGCTGCAGC
GCGCCAGGAGGCCGCCAGAAAGTCGATGCTAAATCCTTATTGGCAAAACTCGAACGACAGAAAGCCACGCTTCTCCTCCCATGACATGGCCATTCAGAAAAGACTGA
ATAAATGGCGCCGGAACTCAAGCAAGATACCCAAAAAGGGTAGGATAGCTTCAAAGGACAACGCAAAGCCACGACCGGTATCCAAGACAAGCAACCCAGCGCAAAAA
CATCTGGAAAACTACCAGGACATGCTCCAAAAAGAAAGGAGTGAACTAAATGACCAGGAACCTAAAAAAGGTACCACAAATCCCATGCGAGTCGGCAAAGATAGCCC
TCCGACCACTAGCAGAGCCGCCAGAGCTAGCTTCAAGCCAAGAATCATTGACGAAATGACGCCATCCCCTAGAAATCACAATCTGTATTTGCAAAAAGGCTACTCGG
ATGACCCCACCACAAACCTAGCCAATAGAGTCGACAACTTAGAGCAGAAAATTGACATTTTAATGGCGCTAATCATACAAGGAAGGAATAATAACAGTGACATGGAT
ACATCCACTTAAATTTAGATATGCTTATATCTATTCTTAATTATATATTTATATCCGATAATAAGCGCACTTTCGCCTACTCCCAAATACATTCATCGACACCACCTTC
CTCCATGCAAAGCCTAAAACAAATAAATGGAAAAATAAATTAATTAGACGGAAGGCATAAAAACTCAAACACTACTCTCACCTCAACGTACCTGGATAATTAAGCAC
ATGACAACGCCCACCAGCTGATCCTGACGAAGCGATACGAAGAAGAACAACACCAAGAATCGGAAGATCGATATAAATAAGATTAGTGCGGCAAAAACAGGATGAGT
ATAAGGCGACTCGCTGCAGCAATATATGCACAACGTCACTTACCTGAATTTTCTTGCCGCTCTGTCTTCTGAAGTATCCTTCCTCACCGTTGCAATCTACACATGTT
GACGAACTGCAAAAGATGGGCCATCTATGTTGCCTCTGCGTCGATCAGGACACTGTGCAGCTCACATAAACTTCCAACTTAACGAGCGCCAACAGCGAGTTGACGCT
AAAACCAAAACCCTAAAAACAAACAAATCATTAAATACAAACAAATAAAATAAAATTAAAACAAACTACCTACCTCAACGACCGCAGCAAATTGCTGACCCACATCC
AACGCAACAGACAACAGGAGACGGGCTCCGCAACGCAAAACAAAATCGCCAATTTTGCGATTTTAAACACAAAAAATTGACAATCTTACGACGCCGTCTCCATCTCC
TGATGCCACTGCCTTAATAAGGATCATTA**GCGCGAA**GCTGACGTCACATTAACAAGCTGCAAAAATCATCCTCAAAATCTATACTTCTCGTCAATACCGTATCTTCC
ACGAATTTTCCGCTTACCTGCAATGAAAGGAAAATCAATAAGAATGCGATACAAAAATTAATTAATGACATATAAATAATAGCAAACCGGACAGACAAAGTAGCAGA
TACTAATAGACGGCTCCATCCTGCTGACGACAAACACGCAACTCCTTCTTCCAAGACTGCAATGCTGAAACAAGGGAACACAAGCTAATACTGGGAAATACAAAATT
CAAAATATAATACTTATCTAATCGCCAACTTAATGATTCTAAATCTACGGCATCCCAGTCGCGACGACACAAAAATAAAGGTCTTCCAATTCAGCCTTTACTAAACG
TACCTGATAAACAAACAATTAATGCAATTATAAAAACAAATATAATACTCACCCCAGACTAGCTCCCTCCAAACGTACCTGAAAAACAAAATCAAAATTAAATCAAA
CATAAAAACAAATAAATAATAAAATAATACTTACCTCAAAACTACCTCTCACCAAATGCACCTGAAAAAATAAAATAAAAACAAATTAACGCATTCACAAAAACAAA
TAACTAATGTAATACTTACCTAATTATAACTTTACATTTATTTCATGCCCGTATCGTTGCGGCGGTCCTTGGCAACAAATCCCGGTCCGGCGGCTCCAAGCTGCCAA
TCCTGACTCAATCGCCACAAGACGCGGCGCACCTGGCTACTCTCGGCGAACAACCGAGCTGC**AATTCC**CTCCGACGACTTCTTGGCCACAACAAGACGGATCCTCC
TACATATTGCCAGCTGTCTATAAAAATGCAATGACTGCTGCGCTGGACCCTCACACTGACGACACAACGA**AATTCCA**CCAAATGACAGC**GGCGCGGGATGC**TGCAAC
CGAGAACAAATTATCTGCAACGCCAGCCGGACATACTTGTTGCAAGTGGCGCGCTTCCAGCGGCCGC

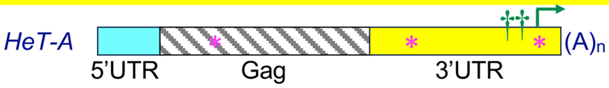

Fig. S14. Identification of potential E2F1- and Sd/dTEAD-binding sites in *HeT-A*. The nucleotide sequence of *Drosophila melanogaster* retrotransposon *HeT-A* subfamily D (clone01D09; GenBank accession #: AJ635224.1) is annotated to highlight key regions and potential binding sites: the 5'UTR is marked in light blue, the Gag coding sequence is underlined, the 3'UTR is highlighted in yellow, potential E2F1-binding sites are highlighted in green and underlined, and potential Sd/dTEAD-binding sites are highlighted in grey and underlined. Key observations: potential E2F1- and Sd/dTEAD-binding sites are predominantly located within the 3'UTR; a potential E2F1-binding site is located within the sequence encoding Gag, presented in reverse complementary form, matching the CGCGGGAA motif. The binding motifs for E2F1 and TEAD are shown in the upper-right corner for reference. The potential dE2F1-binding motifs (TT[C/G][C/G]CGC, such as TTCCCGC, TTCGCGC, or TTGGCGC) are highlighted in magenta.

Fig. S15.

***Drosophila melanogaster* HeT-A non-LTR retrotransposon 23Zn-3**, partial sequence; HeT-A non-LTR retrotransposon 23Zn-1, complete sequence; and HeT-A non-LTR retrotransposon 23Zn-2, partial sequence

GenBank: U06920.2

3'UTR: <1..961

polyA tail of HeT-A non-LTR retrotransposon 23Zn-3: 959..961; 7092..7097

HeT-A 23Zn-1: 962..7097

5'UTR: 1015..1745

gag: 1746..4511

3'UTR: 4512..7097

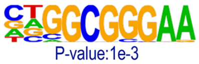

P-value:1e-3

E2F1(E2F)/Hela-E2F1-ChIP-Seq(GSE22478)

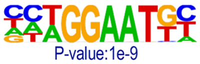

P-value:1e-9

TEAD(TEA)/Fibroblast-PU.1-ChIP-Seq

GenBank: U06920.2

>U06920.2 *Drosophila melanogaster* HeT-A non-LTR retrotransposon 23Zn-3, partial sequence; HeT-A non-LTR retrotransposon 23Zn-1, complete sequence; and HeT-A non-LTR retrotransposon 23Zn-2, partial sequence

```
CTGCAGATGTACCTGAAAAATAAAAACAAAAATCAAAGCAATGATAAAAACAATTAAATACAAATATTACACTTACCTCCAATTTCCCATCTAATGCACTTGAAAAACAAAAATCAATA
CAATCATTAAAACAATTACAATGTAACACTCACCAGATTTTAATTTTAAATTCTTTTCCATGGCCTCAATTGTGACGACGGGCCTCGGCAAAAAATCCTGTTCCGGCGGCCTCAAGCTGC
CAATCCCGACGCTATGGCCATAAGACGCGGCGCTCCTGGCAACTCTTGATGAACGACTCCTCTGCCACAAGAAGACAGATTCCTCCACCATAATGCCAACAGCTCCAAATCAATGTAGAC
GGCTGCGCGGGACCCTTCTTCAAAACTTCCTCTTCCTGACGACTGGCGAAGTTTCCCTAGGGTGAAAGAGATACCCACCAAATGACAGCGGCGCGGGATCCAACCACCACGAATAAACTA
CCTGCAATGCTGGCTGCATATACATACTGCAAGTGGCGCGCATCCATCGCCCGCAACATGCCCCAGCCAAAGTACAACACTTACTCACCTGCAATGTCACCTGAGGCCTCCAAAGACTTG
GTGCTTCCGTCCTTCTGGCGCGTGTACCTGAAAGTAAATCAATTAAACAATGTTAGTCTTAAATCTCAATGTTTTTTGTAAAAATTTTTCAATTGTTAAAACGCTAAACAAACCGCGCAA
TATGATTATGTTACCAGACCCATATTTCTGTCAAAAGCCTAAATTACAAAAATACTAACTACTTTAACATTAACATTAACATTAACTATGTCCAACTCCCAAACTCACCACATGCAATGT
TAAACTCTAAAACTCAAATTATTGTACCTATATATTACAAAAATTGTAATCAAAGGCAAAATAAATTGTGGATGCGGAACAGAATTCACTCTGTCTCCGTACCTCCACCAGCAAAGTTAA
ATTTACTCTGTCTCCGTACCTCCACCAGCAAAGTAAAAAAAAAAAAAAAAAAAATAAATAAATAAAATAAATTAAACAATTAACTAAATAATTAAATAACTAAAATTAATAATATAATCC
GTTCGCTTGCCAAAGACTCTCACGCGCATAACTAATTAAAATCGATTTTCAAGTTGACAAATAAATGGTTTAAAATTGTCCTCAGGCTGCAAAGAAAAGCCGCGGCAACAATAAACATTT
AGTGACACGCGAAAAGCGAACATTTGATTAGTGTAATACTTGTGCAAACCGACAAGCTGCCGCCATAACAAAACGGAGACGAAGAATCATAAAGAACAAAAGCTAAATCCACCAGCATAG
CAAAAATAAATTAACAAATAAAATAAAAGCAAATTTAAATAACATAATAAATTAAACTTATTTAATAAACCAATTAATTTTAATTAATTCAATTAAACGCTAAATCTACATAATACTCCA
CGCGCAAATTAATTGAAATCGTCTTTCTAGTTAATAAATTAAAAGTTTAAAAATTGTCTCCGGCCGCAAAATTTGAACCGCGACGATAAAAACATTTAATTGACAAACAAAAAGCGAACA
ATTATTCAGTGAACTATTTGTGCAAAATTGACAAGCAGACGCCATAATTAAAAGGAGAAGAAGCCAAAAGACGAAGAGAAGAAAGCAACCAGAAGAACTCAAAGAAGAAAAGGAGGAAAG
CCCAATTAAAGAAAGCCAGGGTATTTATACCTTACACTTATCGTTTAATATAACAAAAACCCAACATGTCCATGTCCGACAACCTTTTTTCTGACGATGAGGTACTTTCAATTTCCTCAA
GCCCAGAACAGCGATCTTCTCCGTTCTACCTCAATATATCGCCCATGTCCCACGGATCAGACAATTCTCAGATTAATACAGTCATCATTAATTCGAAGAAATTGCCCTCAAATCAAGCAG
ACATAAGTTTAAAAAACTCTTCTGGGGCTGCTATAAAAATTGTTAATTCCCTTTCACACAAGAAGAAAGAGAACACAAACGTTAATAATGCCCAAAAAGACCCCCTCTCACTCACCAATA
CTACTGCAAGCACTTGTGGCGCCAAAAGCAGCATCTCAGAGGGGAAATTGTCTTCTCCTCCGTCCACCTCACACACATATGAGGGGAAATTACTCACAAAACTTACTCACACACACACAG
ACTTTAGAGGCGCCAAAACGAGCGATGCAATGGGAAGTTTCCCCTCTCTCTCGCACAGCGACAATAGCATAGAGAAAAATCTGAGTTCTTCCACCAAAATTGGACCAAACGCTTCTTCCC
CTCCTTCTCATGCACACACTCACACTAGCAAATCCACTGATATAAGCTTAGAAAGCCGCTCAAAACATCCCGCGCTTGCCAATACGGACGCACGCTCTATAAAAGCCAATGCTAATGACA
ATGGGGAAATTTTCTCCTCACTTATACAAATTGACGAACGCAAGCAAGAGGAAAGGCCTTGCACAACTATCAACGCTTTTTGGTCTATTTTTAAACCCAAGCCGGACGTTACTAAACTAA
GTCTAAAGAGGAAACCCACCAATCCCACTAAAAACACTGGGAAAAAATGCATCTCCCCTCATAAAAAGAGCGCTTATTTATGCCCTTCCGCTCAGGATGATTTAAATTTAAATTTAAACC
CCAAATCTAGCGCCAAGCCCACTGTGGTGAATTTACCAGCTGCCCGCATCCTAAGCCGGCCTGCAGCCAAGCGGGATTTATTTAAATCATCATCCTCCCGAAGCCCAGACGAGCAGCCTA
TGAGTTTTTCGGAAGTGGTCGCTGGCACGGGTTCAATTTTTGCGGCACCCTGTGTCCCGGCACCTTTAACGAAAACTCCAGGCAAGCGGACAAACGACGATCTGGACTGCTCCAACTTTA
AGACGCCCAATAAAAAATTATGCGCGACTTCCAACTTTGTAACTCCCAGCATTTTTCCGCCGCTCATCACTCCCGTTTTCAAGAGCAAGGCAGCTCAATCTGTTTACGAGGAATCCAAAG
CCAGAAATGGACCCCCCCCGCCGGCCCTCGCCTGCAGCATCAATGCCTCTGCTCGCAGCGCAGCGGCGCCACCCGGGATCGCCCCCCTACCCCCTCATAATACAGATGCAGAGCTGCCTC
CATGGAAAATCGTGCCCCAGAGCCGTAGAGCACCTCCTATACTCGTCAATGATGTAAAGGAAATTGTACCTCTACTGGAAAAGCTGAACTACACAGCAGGAGTCTCCAGCTATACTACTA
GGGCTATAGAAGGAAACGGGGTCAGGATACAGGCAAAGGACATGACCGCCTATAACAAAATTAAAGAAGTCCTGGTGGCCAACGGACTTCCTTTATTCACCAACCAGCCCAAGTCCGAGA
GAGGCTTCCGAGTCATCATCAGACATCTCCACCACTCCACACCATGCTCGTGGATAGTCGAGGAACTGCTGAAGCTCGGATTCCAAGCGCGATTCGTCAGAAATATGACGAATCCGGCTA
CAGGTGGCCCCATGCGAATGTTTGAAGTGGAGATCGTCATGGCCAAAGACGGCAGTCATGACAAAATACTCTCACTCAAACAAATCGGTGGGCAAAGGGTGGACATTGAAAGGAAAAACA
GGACACGGGAGCCAGTCCAGTGCTACAGATGCCAAGGCTTCAGGCATGCCAAAAACTCTTGCATGAGGCCGCCAAGATGCATGAAATGCGCTGGCGAACACCTGTCTTCCTGTTGCACCA
AACCAAGAACCACCCCCGCCACCTGCGTAAATTGCTCTGGGCAGCATATTAGCGCGTACAAAGGATGCCCTGCATATAAGGCGGAAAAACAAAAGCTGGCGGCAAACAACGTTGACATAA
ACAAAATAAGAACAATCAAAGACGCAACAAATAACTTTTATAAACGTCAAGGCCCCCCTCTACGCAACAACACCCCTCGGCTACCGCACAGCTCAGCAATCCTGAGCAAATCAATTGCCG
AAGCTCGCCAGGAGGCAGCCAGAAAGTCGATGTTAAATCCATTCCGACAAAATATAAACGACAGAAGACCACGATTCTCCTCCCACGACACGGCCATTCAGAAGCGTCTGAATAAATGGC
GCCGAAACACCAACAAAATACCCAAAAAGGGTAGGATAGCCTTAAAGGATAATGCAAAGCCACGACCGGCACATAGGACAAGTAACCCAGCGCAAAGACATCTGGAGGACTACCAGGACA
TGCTCCGAAGGGAAAGGAGTGAAGAAAACGACCAGGAATCTGAGAAGGGCACCCCCAATACCAAGCAGGTCGGCAATGACAGCCCTCCGACCACGAGCAGAGCAGCCAGAGCCAGCTTTA
AGCCAAGAATCATTGACGATACCACGCCATCGCCAAAAATCTGCAATCCCAACTCACAAAAAGGCCTCTTGGACGACCCCACAACAAGCTTAGCTAATAGAGTCGACAATTTAGAAAAGA
AAATTGACATTTTAATGGCCTTAATCATACAAGGAAGAAATAACAATCTTGACATGGATACATCCAATTAATCTTACAACTACTTATATATTCTTTAATAAATATATCCAATAGAAAAGC
GCACGTCGGTCTGCTTTTAAAATCCTTCACCGTCATCACCTTCCTCGACGGAGCCTAATTTATTGGAAAAATAAATCAATTATATGTTGGCACAAAAATGTAAACACACACTCACCTAAA
CGCACCCGGACGAACAAGCCTATGACAACGCACTCCAGCTGATCTGTAAGAAACAAAAAATATGAATAGATAGATCGATATGAAAAGGATATGTGCGGCAGAAACATGATGAGCAAAAGG
CGACTCGCTGCAGCAACTTATGCACAACGTCACTTACCTGAAATTTCTTGCCGTACGATCTCCTGTAGTATCCCTTATCACAGCTGCAATCTACTTGCAATGCTGCACTGCAATAAACGT
ACTACAAAAGCTGCATACGTTTTGATCAGGACACCTCGTGCGGACGTGCTAAAAAAAATTTCCTTTCTGCTGCTCTTATTGACGCTAAAACCTTAAAACCTACAAACAAAACAATTAAAT
AATAACAAATCAAATAAGACAACCAAATAATACACTTACCTCATTGACTGCAGCTAAATCGCTGACCCACATTCAGTGCAGCCGACAGCAGGAGACGGGCCCGCAAAAGCAAAACAAAAT
CGCCAATTTTGCGATTATAAACACGAAAAATTGACAATTTTGCGATGCCGTCTCCGCCTCCTGATGCCACTGCATTGACAAGCATCACTAGCGAGGAGCTGACACCACACCAAAAAGCTG
TAAAATCCGTCCACAAATTGTATATTTTGCCTCAGTGTCGTATCTGCAATGTTTTTCCGATAACCTGTAAGGAAAGAAAAATTAATAAGAAAATTATACAAAATTAATTAAGGACGACAG
AAAATAGCAAACCAGACAGGCAAATTAACAGATACAAATATGAGACTCCATCCTGCTGCCGACACACAAGTAAATCCTTCAACTCGACAACAGGAGACGGGCCTTGCAAAAGCAAAACAA
AATCGCCAACTTTTGCGATTATAAATACAAAAAATTGACAATTTTGCAACGCCGTCTCCACCTCCTGTTGCCACTGCATTAATAAGGATCACCAGCGCGGCGTGACGCCACACTAAAAGG
CTGCAAAATCCGTCCACAAAATGTATACTTTTCCTCAGTACAATACTTTCTAATGAACTTCCGCCAACCTGCAATGAAAAGAAAAGAAATAGGTATATAAAACAAAACAAACAAAAGGAC
AACCTAAAATTAGCAAACCAGACAGGCATACTAGTAGATGCTAATATGCAGCTCCATCCTACTGACGACAACCACGCAACTCCTTTCTCCAAGACCGCAAATACTGAAACAAGGAAGCAC
AAGCTAATACTGGGAATTATTTATTTAAACAAAAATACTTATCTAATTGCCAATTCGACGACTCCAAATCCGCGGCTAACCGGCGGCGATGGCCCATAAATAAAGGGCCTCCTAATTAAT
TACAAAATGTACCTGAAAAACATAAAATTAACGCAACTATAATTAACGCAATTAATAAATCAAATAAATACAAGTATAATACTTACCTCCAAGCAAACGTACCTGAAAAACAAAACCAAA
AAAAAAATTAATGCAATAAATAAATCAAATAAATACAAACATAATACTTACCTCCAATTTACCTCCCAGCCAATCTACCTGAAAAACATAATCTAATACAATCTCAAAAACAAATAACAA
ATGTAATACTTACCAAATTTTAATTTTGTATTCATTTCCATGACCCCAACGCTGCAACTGTCCTCGGCAACAATTCCTGTTCCGGCGGCTCCATGCTGCCAATCCTGACGCACTGGCCAC
AAGACGCGGCGCTGCTGGCAATCTCTCGATGAACAACCGATCTACAATTTCCATGACGACTCCTCTGTCACGATGAGACAGAAGACACCACCAACGCCAGCAGCTCCAAAACAATACAAC
AACGGCCGCGCGGAACCCATCTTCAGAATTCCCTCTTCCTGACGACCGGCGAACGAGTTCTGGAATAAACAATGTATTAATTGCAAACATCTACCGATGAGGGTAGAAGAGATACTCACC
AAACGACTGCGGCGCGGGAACAAACTAACTGCAACGCCGGCCGGACCTATTTGTTGCAAGTGGCGCGCATCCAGCGCCTGCAACATGCCCCAGCCCAAGTACACAACTACTTACCTGCAA
CGTCGCCAGAGGCTCCCAGCGAATCGGTGCTTCCGTCCTTCTGGCGGGGGTACCTGAAAAGAAACAAATTAAACAATATTAATCCTAAATTTCAATGTTTTTTGTAAAATAATTTAAATT
GTTAAATGTAAACAAGCCTTGCAATATGTTAATGTTACCAGTCCATGCTACTGTCTAAAAGCCAAGAATACAAAAAATACTAATTATAAACTAACTCACCACGCCCAACCCCCAAACTCA
CCCCATGCAATGTTAAACCTATAAATTCAAATAATTGTACCTATATATTGCACATACTGTAATCAAAGGCAAAATAAATCGTGGATGCGGAACAGAATTTACTCTGTCTCCGTACCTCCA
CCAGCAAAGTTAAAAAATACCTCCACCAGCAAAGTAAAAAAAAAAAAAAAAAAAATAAATAAATAAAATAAATTAAACAATTAACTAAATAATTAAATAACTAAAATTAATAATATAATC
CGTTCGCTTGCCAAAGACTCTCACGCGCATAACTAATTAAAATCGATTTTCAAGTTGACAAATAAATGGTTTAAAATTGTCCTCAGGCTGCAAAGAAAAGCCGCGGCAACAATAAACATT
TAGTGACACGCGAAAAGCGAACATTTGATTAGCGTAATACTTGTGCAAATCGACAAGCTGCCGCCATAACAAAACGGAGACGAAGAATCATAAAGAACAAAAGCTAAATCCACCAGCATA
GCAAAAATAAATTAACAAATAAAATAAAAGCAAATTTAAATAACATAATAAATTAAACTTATTTAATAAACCAATTAATTTTAATTAATACAATTAAACGCTAAATCTACATAATACTCC
ACGCGCAAATTAATTGAAATCGTCTTTCTAGTTAATAAATTAAAAGTTTAAAATTGTCTCCGGCCGCAAAATTTGAACCGCGACGATAAAAACATTTAATTGACAAACAAAAAGCGAAC
AATTATTCAGTGAACTATTTGTGCAAAATTGACAAGCAGACGCCATAATTAAAAGGAGAAGAAGCCAAAAGACGAAGAGAAGAAAGCAACCAGAAGAACTCAAAGAAGAAAAGGAGGAAA
GCCCAATTAAAGAAAGCCAGGGTATTTATACCTTACACTTATCGTTTAATATAACAAAAACCCAA
```

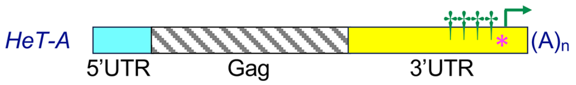

Fig. S15. Identification of potential E2F1- and Sd/dTEAD-binding sites in *HeT-A*. The nucleotide sequences analyzed include the following non-LTR retrotransposons: 23Zn-3 (partial sequence); *HeT-A* 23Zn-1 (complete sequence), and *HeT-A* 23Zn-2 (partial sequence) (GenBank: U06920.2). Annotations follows the same conventions of Fig. S14; the polyA tails of *HeT-A* non-LTR retrotransposon sequences are underlined and shown in dark blue.

Fig. S16.

Drosophila melanogaster **retrotransposon TART-A, clone17G23**
GenBank: AJ566116.1

5'UTR:1..3934
Gag: 3935..6847
Pol: 6885..10127
3'UTR:10128..15576

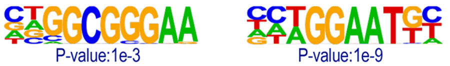

E2F1(E2F)/Hela-E2F1-ChIP-Seq(GSE22478)

TEAD(TEA)/Fibroblast-PU.1-ChIP-Seq

TTCCCGC or TTCGCGC or TTGGCGC

>AJ566116.1 *Drosophila melanogaster* retrotransposon TART-A, clone17G23

[illegible]

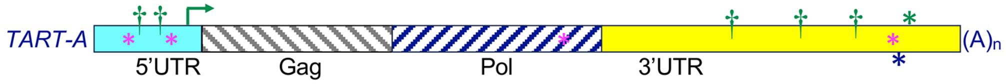

Fig. S16. Identification of potential E2F1- and Sd/dTEAD-binding sites in *TART-A*. The nucleotide sequence of *Drosophila melanogaster* retrotransposon *TART-A* (clone17G23; GenBank: AJ566116.1) is annotated using the same conventions as Fig. S14. Potential E2F1- and Sd/dTEAD-binding sites are present in both the 5'UTR and the 3'UTR. Additional details: within the sequence encoding the Pol protein, a potential E2F1-binding site is identified; however, it contains only the core GCGGGAA sequence, lacking upstream elements. Potential dE2F1-binding motifs (TT[C/G][C/G]CGC) are specifically highlighted in magenta.

Fig. S17.

Drosophila melanogaster **TART-B1 transposon putative single-stranded nucleic acid binding protein and putative reverse transcriptase genes**
GenBank: U14101.1

5'UTR: <1..961
ORF1: 962..4093
ORF2: 4131..7385
3'UTR: 7386..10654
polyA_signal_sequences: 10479..10484; 10600..10605
3' oligo(A) tail: 10637..10654

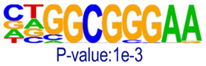

P-value:1e-3

E2F1(E2F)/Hela-E2F1-ChIP-Seq(GSE22478)

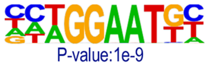

P-value:1e-9

TEAD(TEA)/Fibroblast-PU.1-ChIP-Seq

TTCCCGC or TTCGCGC or TTGGCGC

>U14101.1 Drosophila melanogaster TART-B1 transposon putative single-stranded nucleic acid binding protein and putative reverse transcriptase genes, complete cds

GATCCGGCCGGATGGCAGCTCGACGACGGTAGCAGGCCACCATGCCCGCCAGCCCGCAGCTAGGATAGCAGCAACATCAGCGTCTTATTAACGCGAACGGCGGCACTGGCCCTTAGCTGACCAGCTGCAACAACATCAGCGACG
CAGTCTCCACCAGCAGCAACAACAGCAGCGGCGCTTGCGACAGCAGCAGCAGGGAATGGTTCTGCCGCAGTCGCCTATTGCTGCCTATAAAGAGCATTCCTTGCCAATTGGCTTTCTGTACGACGACGATGATCCTGCGGACAA
ATCCACCTTTCCGGACGCCTGAGTATTATGGCGAGCCTGCTGATCATTGTTTCTCCGACTCCTTACTTGACCACTGGACAATCATACAATCACCGGCTTTTCACATCTGGAAGAAAGAAACCTGGATTAGTATTTGCCCTTCAG
ATTTGTATGAAACTGCATTTCTGGCCAACCGGAGTGTTTCCTGCAGACGACGCTGGACTGCGACGCACTTTTTTCTACCAACACAGATGGGACGCTTGCATCAGATCTGACGGGCCAGACTGGCCGATGCCTGGTACACGATGG
CATCTCCGCGGCGGCGAGATGTGCAGCGACGCTGAAAGCAATAATAATTCTTTTACTTTGCAGTATCAAAAATGAGAAGCCACATCACAATTTACATCAGCAAAAATTACACGTGATATCTTTTTCTTTTGCCTAGGAGTAGTG
AGACCTGCTACCAAAATGACCAGTAATACCGGCTAACGGAAGCGAGATATCGATAATCATTCTGGCGGTAAAATAGCCAGAACATCGATAACCAGCTGTGCATTTTCCCTTCGATTTATCGAAGTCAAATAGCCCGCCTAGGCT
ATCGGACCGGATCGGCAAGTCAGTAGTGATTAAAAATCCGGCAGAGGCGCCTGTGCAGCAGCTACCCAAAGTGAACACGACGAAACGCAACAACAAA**ATG**GACGGACATAATGGAGATCAAAGTGAAGGATGGGCAACAGTGCTATCTAT
CTCCTCGGATGATAGTAACTCCTTGTCGTCGCCGCCGTCTATAATAGTTTCATCGCTGGACACCACGCCAACTTCGCACGAAACCACCATAGTAAGAAGAAGCCTATATCAAACAAATGCTGACATGAAATCTTACGATTTTGAAAATATTGTATTAAATG
AGAATAAAAATACCATATTGCCAGACCCTTTATTTGTTGATAAATGCGGGAGTACTGCTAATACCACAGAAGCCAATGAAAAAAAGCCTGCCAATAGCCCCTTCCCCATTTCCATTAGCAAGAATTTTAGCACTTCCTCGCCACTAACACATGTAGACACG
CCTACACAAGAAGATGACGCCAGTGCGTTCAATACATTGAAAGCAGCTAAAACAGCTAGAATAATTTTTCCCACACACACACATATAAAGCCCACCAAACCCTCGCCCCCCTCCAAAGAATTAAGCACAAACTCAGCACTAAAAACTCTCAGCTACACAGA
CAAAATAACAGGAACACAGAAAAATCTACCCGACAAAACACATGTAGACACACCTACACAAGATGACGACATTAATGCAACCAAAGCATCAAAAACCGCTAAAATAATTTCTACACAGACACACCTAGGTGAAACCAAGCCAATACAGCCCGCCAAAGACC
CCTCACCCCGCACCCAAAAGCCTATTGCACACAAAGCTGACGAGACACTTACCCACACAGACAAACTCACAGCATCACAAAACCTCGTTCCCGCCAAAACACATATAAACACACCCACACAATATAACGACACTAATGCAACCAATGCATTAAAAACCGCT
AAAATAAATTTCTCTTCACACTCACACCAAAGTGAAATCAAGCCAACACAGTCCGCCAAAAACATTTCACCCCTCACACAAAAGCAATTCACAAGCGAATCAGCCGGGACACACACCCATACAGACAAACACAAAAACACAGCATCACAAAATCTCTTTTC
CGCCAAAACACACATTAACTCACCCACACAACATAACTACACTAGTGCAGCCACAGCATCAAAAACCGCTAAATTAATTCTCTCCCCACACTCACACCTAAGTGAAACCAAGCCAACACAGCCCGCTTTAAGCCCCTCGCCCCTCTCCCAGAAGCAAATAA
CAAGCATAGCAGCCAAGACACTCACCCACACAAACAAACACACAGCATCACAAAACTTTATTCCCGCCAAAACACATATAAACATACCCACACAATATAACGACACCAATGCAACCAAAGCATTAAAAACCGCAAAAGCAGCTTCTCCCTCCCACACATAC
TCACGTCAGACAAAACCAATAAAGTCCGCCATAAACGCATTGCATCCCGCCCAAGACACAAACCCAAGCCCAGCAATCAGTGCTGTCACTTACACAGACAAACCCACAGCTACTCAGAATATTTTTCCTGTCAAAACTTTTGCAGAGCTGGTTAGGGAAAA
TGCAAAACGCTTACCAACTCCAATGCAGAATTCCCATCAAGCTAAAAATGACTCTGCCGCTCTCGGACGCCCCCCGACTGCAGCTAGAAAAAATCTAAATAAAACACTGATTTCTCCTAAAACTCCTGGGAAGCGCCGTGGGGACTGTCTTGATGAAGGCC
TACTACAAACCTCTAACAAAAAGGTTAGAATACGCGATGACTTCTCTGACGATGATCTTGGGGTCACAAATCTACTCTCTGAAACACCCTTATTCAAAAGCAAAGCAGCTATCAAGATCCGGCAAGACTCGAGAAGAGATTCCCTGCAGAAGTCAGCCGAA
ATGGACACAGCTCCAGCAATTAGTCCCTCAAACACAGCAGCCGATTCCGACCTACCGCCCTGGAAAACTGTTCCAGCTAGCAGAAAACCACCATCAATCTTCTTGTCCAATATACAGCAGATTATCCCGCTAATAGAAAAACTAAACTATAAAGCCGGGGT
AAATAGCTTTACCACCAAATCTGAACTTGGCAACAATATTAGAATCCAGGCTAAAACGATGGACGCCCACAATGCAATTCAGAATGTTCTCCTTGAGGCAAATATTCCCCTACACTCCCACCAGCCAAAGAGTGCAAAGGGCTTCCAAATTGTAATTAGGC
ACCTCCACCAGTCAACCCCGACCAAATGGATTGAAAGCCAACTTCAAGACATCGGTATAGCTACAAAATTTATCAGGGCAATGCAGTTTAGGGACACGAGAAATCCTATGCGCATTCATGAGGTTGAAGTTGTACCCAAGGCTGACGGCAGCCATCTCAAG
GTCCTGCTACTAAAATCCCTTGGAGGTCAAACGGTCAAGGTTGAAAGGAAACGGGTATCGAAGGATCCCACACAATGTCATCGCTGCCAATGTTTTGGGCACACAAAAAATTATTGTAGAAACCCGTTTAAATGTATGAAATGTGGCCAGCTGCACGCCAC
GGTTTCATGCACTAAACCCAAAAACCTTCCGGCTACTTGTGCAAACTGCAATGGAAGCCACGTAAGCAGCTATAAAGGATGTCCCGCTTTCCAAGAAGCAAAGCAAAGACTATCTATCAACAAAATTCAATCCCTACACTCACAACCCACGCACCTTCAGA
CCCCCCGCAATAAACATCCCTACCCAAAACCCACCCACCTTCAGACGCCCCGCAATAAGCAGCCCTACACACACCCCCTCCCTCGCACATCAGTAAACAACACAAAACTACCTGCCAAAAGAATCCAAGGAAAGAAGATATCGCAAAGGAATCTATCTATA
AACAAACGCTTACACAGAATGAAAAAACCGAGGAAGGAGACAAGCCCGCCGACAACTAGCAAAAAGGTCTTGGCCTCTCTAGAAGAAAGCAGAAAAAACCCAAATAGCGTCCTAAACCCGGCCAACACCCATCTCACTCATTTCCGCCCACCCCCTTTAGC
ACAAAATATTCCTAATGACGAACCTAAGGAGCTGAGTGGGGAGCAATACCTTCTAAATCGCATTGAAGGGATGGAAAAGAAGATCAACAACCTTCTTGAAATCGTCACCCGCCTACTAAGACAAGGAAAAGACTGTCCAAAGTCTCCAAAAAATCCTTTCC
GAGATCCAATCTTCGTGTAAATGCTCTTTCTAGTAACATCAGAAAGTGACGTTTCCT**ATG**ACTCGGGCTGACAACAGGGACATCCTTAAAATCGCTTTCTGGAATGCTGGTGGGATTAACAATAAAATAGATGAGCTTAAGCTGTTCATTCTAAATATTGA
TGCCCACATAATCATAGTCACCGAAACTAGACTAGACAACAATTCTACCAAACTAGAGCTGCCAGGATATTTCACATACTTAGCCCAAAATCCTGTCTCTAGCAAGAGAGGAGGGGTCGCTACTATAGTAAACAGCAGTATCCGACACATGGCCCTAAAAC
CGATCGAAAAAGAATGCATACAAAGCGCCCCAATAGTCCTGCTGCCTGAAAACAACAGACGCAGCGAAATGATAGTAATAGCATCTGTCTACTGTCCGCCCTCGCTAAGGTGGTTGCCCCACCACTTTACTGACGTTCTCAATTTTGCAGAGAAAACTTTA
GGAGGGCAGACCAAGTTTATCCTATGTGGCGACTGGAATGCAAAACATAGACAATGGGGTTGTACACGCGCCTGCCAACGTGGCACCGCACTCTACGAAGCAGTCCAAGCCGACTCCACTGCAGAGATCATTGCCACTGGCTGTGCTACACATTTCCCACA
TGACACAAGAAAAAACCCGTCAGCAATAGATTTCTCCATTTGCAAAGGGCTTGGCAGGTTTGAAAAAAGAATTTCTTCAGGGGCGGATCTATCCTCAGACCATCTTCCCATCTTGTTAGAGATAAACCTAGACACAAACACCCTCTTCTTGCAAAAACAAA
ACAACAATATACTCAAGAAAAATACAAACATCGAGCTGTTTAAGAAAGTTCTAGAAAGAAAGATACTATTAAACACTGAGATAAGGGTAGCAGAAGATATAAATGACGCCATAAGCACCTTTATGAAAAACATCAAGGACTCGGCTGCGGAATCAACTCCC
TCCCCAAGAATACGTGACAACCCCAGAAGAAGACATAGGCAAGCCAACAGAAACAGTCATACCCTCGCACTAGACGAAAACACAAGCAGACTGCTTGAGGAAAAACGCATATTAAGTAGAGTTTTCAAAGCTACCAAAAATTACGAGGACAAAGCTAAACT
AAAGGCAGCTGAAAACCGACTAAAAAAAGCGATAAAAATATTGAGAGAAAACAGAATCAATGAGCAAGTTGAAGGAATAGATACAAGTAACCCGGACAGAATGAGAAAAATGTGGAAACTGCTAGATGAAGGGAAAAGGACAAATCAGCCTAATTTTCCCC
TCAAATTAGAAACCCAAAAAGGACCCAAATGGACAAAAACGATTAAGGAGACAACAGAAACATTTGTCTCCCACTTGGAAGGAAGATTTAAGCCAAACAATAATGTACCTGATTACCACATAGATAGAGTCAATACTGGACTAAGAATAATTAAGGAAAGC
ATGCTAACTGAACGACATAATCTGAACAAAAACCCCCATAACCAACCTATTACTCTAAAGGAATTAAATGACGAAATAAAAAACTTAAAGAATAGTAAAGCACCGGGAAAAGACCTTATAACAAACCAGCTAATAAAAACCCTACCGACTAAAGCTACCCT
TTACCTTATCCTAATCTATAACTCCATACTTAGACTAGGATACTACCCAGAAGCCTGGAAACATGCGCAGGTAAAAATGATTCTCAAGCCAGGGAAAAGTGCAAACGAGCCAAGGTCATATAGGCCGATTAGCCTACTCTCGGGACTCTCAAAAATCTTTG
AAAGACTACTCTTAAAAAGACTTTTTAAGGTAGATTTATTCAAAAAAGCTATTCCTCTGCACCAATTTGGCTTTAGAAAAGAACACGGATCGGAGCAGCAAATAGCCAGGGTCACCCAGTTCATTCTTGAGGCCTTCGAGAGAAAGGAATACTGCTCCGCG
GTCTTCCTTGACATCTCTGAGGCTTTCGACAGGGTATGGCACGAAGGCCTTTTACTTAAATTAGCTAAGATCCTACCTTACAACTTGTACATTATACTGGAGAGCTACCTAACAAATAGAACGTTCGAAGTTAAAGACCAAGCTGGAGAGACTTCGAGAAC
AGGACAAATTGGTGCTGGTGTGCCTCAAGGAAGCAATCTTGGACCACTACTCTACTCTATCTTCTCCTCAGACATGCCCCTTCCATATATCTACCGCCCCTCACCAACAGAAAGAATCATGCTCTCAACATACGCAGACGACACTATAGTCCTCAGCTCAG
ACACACTAGCAACTGCCGCTACAAGAAACAACGAAAACTACCTCAAGTCATTCTCGGACTGGGCGGACAAATGGGGTATATCAGTAAACGCTGCTAAAACCGGACATGTCATTTTTACATTAAAAAACGACTTACCTACAAGTCTAAGGACTATGAAGATC
AAGGGTCAAGTAATAAAGATTGAAAGCAAACAATCATACCTTGGCGTAATCCTTGACAGCAAGCTAACCCTCAGCTCCCACGTCACAAAGCTGATGGGCAAATACACTACAGCCTACAGAAAAATGACATGGATTCTAAACAGAAGAAGCAAACTCCCTAC
CAAGACTAAAATGCTGATCCTTAAGTCAGTCCTTTCACCAATATGGCAGTATGCCATAGCAGCTTGGGGTCCCCTTGTGACGGATGCACAGATAAGACGGATCCAAGTCGAGGAAAATAGGAAAATGAGAGACATATGCAGAGCGGGTAGATACACGAGCA
ACCAAACTATAAGGGACCGCTATGGAATCAAAACAGTAGAAGAATTCTATCAACAGGCACTCCACAGGTTCTCAGAAACAATAAAATCTCACCCAAATATAGCTGTACGCAGGATATTCACAAGGCACTATATCCCGAACAGACTAGAAAGAAGCAGGCAG
AGGTACTTGAAAATGACAAATGAACACATCACGCAAAAACAGACTGGACAAACACTCTCACCTAAACTCCTAAAAATCCCTGATCTAAATGACTGCAGAACCCTTAAAAAGCGGAATGAAAGAGATAAAATAAGGCAAACCCATCTAATTGAACTCCCCAC
CCTGCTGAGACTAGAGGAAGAAGAGGAAGAGCTTAGAAGAATAAAAAAACAGGAAGAGAGGGAAAGAAGGGAAAAGGAAACCCAAAAGTGGCCTCCAGACAGATGGTGTGAATTAGAAATTAACCTATATAATAAGCAATATAGAAGGGGCGATCTAACCA
GGCAGGAAATCATACAAAAATTTAGAGGACAACCACTAAATGTACAGAGGATAATTTTACCCGACTATAAAGGAGACCAGGAACACAATTAAAAGTAAATCATGGGAAAGCAGGGGAAAAAAAACAGAAGTAACAAAATGGCGGA
AGGGGTGGCAAAATATATATAAAAATAAGAAAAGATAGAAATAAAAAGGCTAAAGGCTCACTTACAGGTTAATTCAAAAGGGAAACGTGCTTACATATCTAATGGTAAAAACTAACTTATAAC
ACCTACTGGATGACATAGTGAGGATGCCACAGGTTGCGCACTTGAATCACGCTTTCCTGGCGAGATTGTTAAGGCATCACCGGGATCCCGTCTCTCTTTCCTAACTGGAGGACTCATGCCTGA
AATCTTTTCCGCTTCACCGGCACCTGATGGCCATATCTGCTACTGCGCCTTGCGTCCACGGACGCTCCTTCCTGAAAGAAAGAGAATTTGTTTTAGTTTTCCTTTCTCTTCTTAAAAATATAA
CAAAAACAGCAGCAGTAGCCGACCAACAACATACTTAGCTTACCAAGGCAGCAGCAATGATACCAACAGAAGCCCTACAGTGTGCTGAACAATATGCCGCCCACGCCCACCACTTCTGGAACT
CAGGAATGGGGGGTACGGAATGCCGGAAACTGGAGGATTCGCCTCAAATGTTACGCTTACATCTTTGAGCAAATCGCCTGCCTCCTCGTCTAACGGCCCGGCGTCTTGGTACGCCCAGAAGTA
GCAGCGGTAGCAGGATTAGCAGCCTCCACAGAGATATGTTCATCCCAGCATCAGTCCAGTTGGCATTGTGATGCCGCCGTAGCGTACGCCCTCAGAGACCCGACTGGAATCCCAGGACAAGAG
ACGGCAGCACGCCTGGGCTTAGGC**GCGGGA**CAACATGCAGAGATTCGTCAATGAGAATGGCGGCACTGGAATCACCTTTCAGCTGA**GCGGGA**TAATGAGTCGGCAACGCACCGCACTATCAGC
AGCAATAACAACATCGGCAGATGACCTGGAAGAAAGGAAAAAACATGTATCAGTCTTTTCTTTTCTTTTTTTTTCCCATTAGCATGCGTTTTTAA**ATTCC**ATTCCTGAAAACCGGCGTTTTCC
TGTAAAAGACACTGGACGGCGGCGTTTGTTTTAAATGCAAAGATTGGATGCTTGCATCAGGGCTGACGTCCTTGAATAGCCGATGCCTTGTAATTGCCTGCATAGCCGCGGAGGTCCTGATGA
CGGATGCTGGCCTGTATTTGGCCACCACTCCGATGCCATGACAGCAGCAACAGCAGCGGTTTTTGCAAATGAGAGGGATCAGGAGGCGGCAACACATTCACCCGTAGCAGCAACAACAAGCAG
TGGTAGCATCAGCAGGGAGTCTTCTCGCCACAATAGCCATTTGGAAATTTCTCGACGACCCGACTTCCTGCTTGGCCCATGGTCAATCACACTGGACCAGTTAACAGCGGCGTTTTACCCTGG
AAGAAAGAAAACTTGTATTATTCATTCCCTTTTCTCTCACAGTTGGGTATTTAAGTGC**ATTCC**TGGCCAACCTAGCGTGTCTCCTGGCAATGACGTCGGACTCAACGCATGCTTCCCTTCCGA
CACAGTAAGGATGTTCGCATCTGGGCTGACGGGCCAGGCCGGTCCGTTGCCATACATGCTAGCGTTTCCGCGGCGGCGTAGTGAACATCAAACCGTATAATGGCGAAGACGACGATGATCCGG
CCGGATGGCAGCTCGACGACGGTAGCAGGCCACCATGCCCGCCAGCCCGCAGCTAGGATAGCAGCAACATCAGCGTCTTATTAACGCGAACGGCGGCACTGGCCCTTAGCTGACCAGCTGCAA
CAACATCAGCGACGCAGTCTCCACCAGCAGCAACAACAGCAGCGGCGCTTGCGACAGCAGCAGCAGGGAATGGTTCTGCCGCAGTCGCCTATTGCTGCCTATAAAGAGC**ATTCC**TTGCCAATT
GGCTTTCTGTACGACGACGATGATCCTGCGGACAAATCCACCTTTCCGGACGCCTGAGTATTATGGCGAGCCTGCTGATCATTGTTTCTCCGACTCCTTACTTGACCACTGGACAATCATACA
ATCACCGGCTTTTCACATCTGGAAGAAAGAAACCTGGATTAGTATTTGCCCTTCAGATTTGTATGAAACTGCATTTCTGGCCAACCGGAGTGTTTCCTGCAGACGACGCTGGACTGCGACGCA
CTTTTTTCTACCAACACAGATGGGACGCTTGCATCAGATCTGACGGGCCAGACTGGCCGATGCCTGGTACACGATGGCATCTCCGCGGCGGCGAGATGTGCAGCGACGCTGAAAGCAATAATA
ATTCTTTTACTTTGCAGTATCAAAAATGAGAAGCCACATCACAATTTACATCAGCAAAAATTACACGTGATATCTTTTTCTTTTGCCTAGGAGTAGTGAGACCTGCTACCAAAATGACCAGTA
ATACCGGCTAACGGAAGCGAGATATCGATAATCATTCTGGCGGTAAAATAGCCAGAACATCGATAACCAGCTGTGCATTTTCCCTTCGATTTATCGAAGTCAAATAGCCCGCCTAGGCTATCG
GACCGGATCGGCAAGTCAGTAGTGATTAAAAATCCGGCAGAGGCGCCTGTGCAGCAGCTACCCAAAGTGAACACGACGAAACGCAACAACAAAATGGACGGACATAATGGAGATCAAAGTGAA
GGATGGGCAACAGTGCTATCTATCTCCTCGGATGATAGTAACTCCTTGTCGTCGCTGCATATATAAAATAATAAATTTCTTAAAATAATTTCATCGCTGGATTCCTGCGCCACCATAAATTAC
TTATAGAATACACTTAACACTGTACCCACTAATGCTAAAAATTTTCTTATACAATTTTACTTTAAAGCTAAAACTACTTAAACTATGGCGCGTAAAACGGCCGTCAAAAATATCCAAAAATTG
TACTTGCCATAAACAAATTCTAACATAATGTAAATCCTAAATCGTAAAATCATGGCGAACATAACCGCCGCCAAGCAAATCAAAAAATGTTCCTAGAAATTGTAAATCTTAAACCTACTTCTC
TAACTAAAGCGCGCACAACTGCCGCTGATCCGAAATTGTAAAATGCCATAAATTGTAACTATATATTTTATAAAAAAAAATCAGAGTACAATAAAATGACAGCGCCGAAAGGCACTGAAAATAT
TTAAAAAAAAAAAAAACTGCCGCTGATCCGAAATTGTAAAATGCCATAAATTGTAACTATATATTTTATAAAAAAAAATCAGAGTACAATAAAATGACAGCGCCGAAAGGCACTGAAAATATTT
AAAAAAAAAAAAAAAAAA

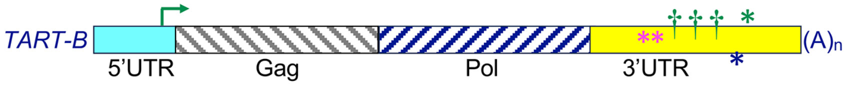

Fig. S17. Identification of potential E2F1- and Sd/dTEAD-binding sites in *TART-B1*. The nucleotide sequence of *Drosophila melanogaster TART-B1* transposon, including regions encoding the putative single-stranded nucleic acid binding protein and putative reverse transcriptase genes (GenBank: U14101.1), is annotated following the same conventions of Fig. S14. Potential E2F1- and Sd/dTEAD-binding sites are identified in the 3'UTR of *TART-B1*. Potential dE2F1-binding motifs (TT[C/G][C/G]CGC) are specifically highlighted in magenta.

Fig. S18.

***Drosophila melanogaster* transposon *TART-C1* gag protein and pol protein genes**
GenBank: AY600955.1

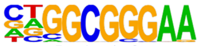

E2F1(E2F)/Hela-E2F1-ChIP-Seq(GSE22478)

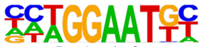

TEAD(TEA)/Fibroblast-PU.1-ChIP-Seq

TTCCCGC or TTCGCGC or TTGGCGC

5' Repeat: 1..331
ORF1: 206..3349
ORF2: 3350..6628
3'UTR is not marked or highlighted
3' Repeat: 10383..10713

>AY600955.1 Drosophila melanogaster transposon TART-C1 gag protein and pol protein genes, complete cds

AAGCTTCGCGGGATATCGATAACAATCAGGTGGTTAAAAATACTATAACATCGAGTACCAGCCGTGTTTTTCCTGTCGATTTATCGAAGTCCAACAGCCCGCCTTCGCTCCGAACACAAATCGGCAAGTCAGT
AGCTTTTTAACCCGGCAGTGGCGCCTGTGCAGCAGCTATTTAAAGTGAAAAACACGAAACGCAACAACAAAATGGACGGCCATAATGGAGACATAAATGAAGGATGGGCAACAGTACTATCTATCTCGTCGGATGAT
AGTAACCAACTTTCGTCGCCGCCGTCAATTATAGTCTCATCGCTGGACACCACGCCAACGTCAAACGAAACCACCATAGTAAGAAGAAGCCTACACAACCCAAAAGCTGACATGAAATCTTACAGATTTGAAAATATTGTATT
AAATGAAAATAAAAATACCATATTGCCAGATCCTTTATTTGTTGATAAATGCGGAAATACTGCTAATACCACAGAAGCCAATGAAAAAAAGCCTGCCAATAGCCCCTTCCCCATTTCCATTATCAAGAATCTTAGCACTTCCT
CGCCACTAACACATGTAGACACGCCTACACAAGAAGATGACGCCAGTGCATTCAATACATTGAAAGCAGCCAAAACAGCTAGAATAATTTTTCCCACACACACACAGATAAAGCCCGCCAAACCCTCGCCCCCTTCCAAAGAA
TTAAGCACAAACTCAGCACCCAAAACTCTTAGCTACACAGACAAAATTACAGTAACACAGAAAAATCTACCCGACAAAACACATGTAGACAGACCTACACAAGATGACGACATTAATGCAACCAAAGCATCAAAAACCGCCAA
AATAATTTCTACACAGTTACACCTACGTGAAACCAAGCCAACACAGCCCGCCAAAGACCCCTCACCCCGCACCCAAAAGCCTATTGCAAACAAAGCAGCCGAGACACTCACCCACACAGACAAACTCATAGCATCACAAAACC
TAGTTCCCGCCAAAACACATATAAACTCCCCCACACAATATAACGACACTAATGCAACCAATGCATTAAAAACCGCTAAAATAAATTTCTCTTCACACTCACACCAAAGTGAAATCAAGCCAACACAGTCCGCCAAAAACATT
TCACCCCTCACACAAAAGCAATTCACAAGCGAATCAGCCGGGACACACACCCATACAGACAAACACAAAAACACAGCATCACAAAATCTCTTTTCCGCCAAAACACACATTAACTCACCCACACAACATAACGACACTAGTGC
AGCCACAGCATCAAAAACCGCTAAATTAATTCTCTCCCCACACTCACACCTAAGTGAAACCAAGCCCACACAGCCCGCTTTAAGCCCCTCGCCCCTCTCCCAGAAGCAAATAACAAGCATAGCAGCCAAGACACTCACCCACA
CAAACAAACACACAGCATCACAAAACTTTATTCCCGCCAAAACACATATAAACATACCCACACAATATAACGACACCAATGCAACCAAAGCATTAAAAACCGCAAAAGCAGCTTCTCCCTCCCACACATACTCACGTCAGACA
AAACCAATAAAGCCCGCCATAAACGCATTGCATGCCGCCCAAGACACAAACCCAAGCCCAGCAATCAGTGCTGTCACTTACACAGACAAACCCACAGCTACTCAGAATATTTTCCTGTCAAAACTTTTGCAGAGCTGATTAG
AGAAAATGCAAAACGCTCACCAACTCCAATCGAAAATCCCCCTCAAGCAAAACATGACTCTGCCGCCCTCGGACGCCCTCCGACTGCAGCTAGAAAAAATCTAAATAAAACACTGATTTCTCCTAAAACTCCTGGGAAGCGCC
GTGGGGACTGTCTTGATGAAGGCCTACTTCAAACCTCTAACAAAAGGTTAGAATACGCGACGACTTCTCTGATGATGATCTGGGGGTCACAAACCTACTCTCTGAAACACCCTTATTCAAAAGCAAAGCAGCTATTAAGATT
CGGCAAGACTCGAGAAGAGAATCCCTGCAGAAGTCAGCTGAAATGGACACAGCTCCAGCAATAAGTCCCTCAAACGCAGCAGCCGATCCCGACCTACCGCCCTGGAAAACTGTTCCAGCTAGCAGAAAACCACCATCAATCTT
CCTGTCCAATATACAGCAGATTATCCCGCTAATAGAAAAACTAAACTATAAAGCCGGGGTAAATAGCTTTACTACCAAGTCTGAACTTGGCAACAATATTAGAATCCAGGCTAAAACGATGGACGCCTACAATGCAATTCAGA
ATGTCCTCCTTGAAGCAAACATTCCCCTACACTCTCACCAGCCAAAGAGTGCAAAGGGCTTCCAAATTGTAATTAGGCACCTCCACCAGTCAACCCCGACCAAATGGATTGAAAGCCAACTTCAAGACATCGGTATAGCTACA
AAATTTATCAGGGCAATGCAGTTTAGGGACACGAGAAATCCTATGCGCATCCATGAGGTTGAGGTTGTACCCAAGGCTGACGGCAGCCATCTTAAGGTCCTGCTAATAAAATCCCTTGGAGGACAAACGGTCAAGGTCGAAAG
GAAACGGGTATCGAAGGATCCTACACAATGCCACCGCTGCCAATGCTTTGGACACACAAAAAATTATTGCAGAAACCCGTTTAAATGTATGAAATGTGGCCAGCTGCACGCCTCGGTCTCATGCACCAAACCCAAAAACCTTC
CGGCTACTTGTGCAAACTGCAATGGAAGCCACGTTAGCAGCTATAAAGGATGTCCTGTTTTCCAAGAAGCAAAGCAAAGACTATCTATCAACAAAATTCAATCCCTTCACTCACAACCCACCCACCTTCAGACCCCCCGCAAT
AAACATCCCTACCCAAAACCCACCCACATTCAGACGCCCCTCAATAAGCAGCCCTACACACACCCCCTCCCTCGCACATTAGTAAACAACACAAAACTACCTGCCAAAAGAATCCAAGGAAAGAAGATATCGCAAAGGAATCT
ATCTATAAATAAACGCTTAAACAGAATCAGGACATTGGACAGAAAACCGAGGAATGAGACAAGCCCGCCGACAACTAGCAAAAAGGTCTTGGCCTCTCTAGAAGAAAGCAGAAAAAACCCAAATAGCGCCCTAAACCCGGCCA
ACACCCATCTCACTCATTTCCGCCCACCACCATTAGCACAAAATATTCCTAATGACGAATCTAAGGAGCTGAGTGGGGAGCAATACCTTTTAAATCGCATTGAAGGGATGGAAAAGAAGCTCAACAACCTTCTTGAAATCGTC
ACCCGCCTACTAAGCCAAGGAAAAGACTGTCCAAAGTCTCCAAAAAATCCTTTCCGAGATCCAATCTTCGTTTAAATGCTCTTTCTAGTAACATCAGAAGTGACGTTTCCTATGACTCGGGAGTGCAACAGGGACATCCTTAA
AATCGCTTTCTGGAATGCTGGTGGGATCAACAATAAAATAGATGAGCTTAAGCTGTTCATTCTAAATATTGATGCCCACATAATCATAGTCACCGAAACTAGACTAGACAACAATTCTACCAAACTAGAGCTGCCAGGATATT
TCACATACTTAGCCCAAAATCCTGCCTCTAGCAAGAGAGGAGGAGTCGCCACGATAGTAAACAGTAGTCTCCGCCACATGGCCTTAGAACCGATTGAAAAGGAATGCATACAGAGTGCCCCAATAGTATTACTGCCTGAAAAC
AACAGACGCAGCGAAATGATTGTAATAGCATCTGTCTACTGTCCGCCTTCGCTAAGCTGGTCGCCCCACCATTTTACTGACGTTCTCAATTTTGCTGAGAAAACTATGGGAGGGCAGACTAAGCTCATTCTATGTGGCGACTG
GAACGCAAAACATAGACAATGGGGTTGTATACGCGCCTGCCAACGTGGCGCCGCACTCTACGATGCAATTCAAGCAGACTCCATGGCTGAAATCGTCGCGACTGGCAGCGCTACACATTTCCCGCACGATACAAGGAAAAGCC
CGTCAGCAATAGACTTCTCGATATGTAAACGGCTTGGCAGGTATGAAAAAAGAATCTCCTCAAGTGCACACCTATCCTCAGACCATCTTCCCATATTACTTGAGATAAACCTAGATATAAAAACCATCTCCCTGCAAAAACAA
AACAACAATATCCTCAAGAAAACAACGAACATTGAGCTCTTTAAGAACGTTCTAGAAAGGAAGATACTTCTAAACACTGAGATAAGGGTAGCAGAAGACATAAATGACGCCATAAACATCTTTATTAAAAACATCAAGGACTC
GGCTGCTGAATCAACTCCCTCCCCAAGAATTCCTGATAACCACAGAAGAAGATATGGGCAAGCTAACAGAAATAGTCATACGCTCACACTAGACGAAAACACAAGCAGATTGCTGGAAGAAAAACGTATACAAAGTAGAATTT
TTAAAGCTACTAGAACGAACGAGGACAAAACTAAACTAAAAGCAGCTGAAAATCGACTTAAAAAAGTAATCAAAATCTTAAGAGAAAAGAGAATCAATGAGCAAATTGAAGGAATTGACACAAATAACCCGGACAGAATGAGG
AAAATTTGGAGGCTGCTGAGTGAAGGGAAAAAAATGAATCAACCCAACTTTCCCCTCAAATTAGAAACCAAAAAAGGCCCTAAATGGACTAAAACAATTAAGGAGACAACAGAAGCGTTTGTCTCCCACCTGGAAGGAAGATT
CAAGCCAAATAAAATTGTACCTGATTACCACATAGATAAGGTTAACACCGGACTAAGAATAATTAAGGAAAGCATGCTAACAGAACGACATAATCTAAACAAAAACCCCCATAACCAACCCATTACGCTAAACGAATTAAATG
AAGAAATAAAAAACTTAAAGAATAGCAAAGCACCTGGTAAAGACCTTATAACAAACCAGCTCATAAAAAACCCTACCGACTAAAGCTACCCTGTACCTTATCCTAATCTATAACTCCATACTTAGATTAGGATACTACCCTGAA
GCCTGGAAACATGCACAGGTAAAAATGATCCTGAAGCCAGGGAAAAGCTCAAACGAGCCGAAGTCATACAGGCCGATTAGTCTACTCTCGGGACTCTCTAAAATGTTTGAAAGACTACTCCTAAAAAGACTTTTTAGGGTAGA
TCTATTCAAAAAAGCCATACCACTGCACCAATTTGGCTTCAGAAAAGAGCACGGAACTGAGCAGCAAATAGCCAGGGTCACCCAGTTCATCCTCGAGGCCTTCGAGCGGAAGGAATACTGCTCAGCGGTTTTCCTTGACATCT
CTGAGGCCTTTGATAGGGTATGGCACGAAGGCCTTTTACTTAAATTAGCTAAGATCCTACCTTACAACCTATACATTATACTGGAGAGCTACCTTACAAATAGAACGTTCGAAGTTAAAGACCAAGCAGGAGAGACTTCGAGA
ACAGGACAAATAGGCGCAGGAGTGCCTCAAGGAAGCAATCTCGGACCACTACTTTACTCTATCTTCTCCTCTGACATGCCCCTCCCATATATCTACCGCCCTTCACCAACACAAAGAATTATGCTCTCAACATACGCAGACGA
CACTATAGTCCTCAGCTCAGACACACTAGCAACTGCCGCCACAAGAAACAACGAAAACTACCTCAAGACATTTTCGGACTGGGCGGACAAATGGGGTATCTCAGTAAACGCTGCTAAAACCGGACATGTCATTTTTACATTAA
AAAACGACTTACCTACAAACTCAATGAATGTGAAGATCAAGGGTCAAACAATAAAGAAGGAAAGCAAGCAATCATACCTTGGGGTAACCCTTGATAGCAAGCTAACCCTTAGCTCTCACGTCACAAAGCTATTGGGTAAATAC
TCTACAGCCTACAGAAAATTGACATGGATCCTAAACGGAAGAAGTAAACTCCCTACTAAAACTAAGATACTGATCCTTAAATCAGTTTTATCACCAATATGGCAGTATGCCATAGCAGCTTGGGGTCCCCTTGTGACAGATGC
ACAGATAAGGAGGGTCCAGGTTGAGGAAAACAGAAAAATAAGAGACATATGTAGAGCGGGAAGATATACGAGAAACCAAACTATAAGGGACCTTTTTGGCGTCAAAACAGTAGAAGAATTCTATCAACAGGCTATGCACAGGT
TCTCAGAAACTATAAATCGCACCCAAATATAGCTGTTCGCAGGATTCTCTCTAGGCACTATATCCCGAACAGACTAGAAAGAAGCAGGCAGAGGTACTTTAAAATGACAAATGATCATATCACGCAAAAGCAGACTGGACTT
GCCCTCTCACCTAAACTCTTAAAAATCCCTGATATAGATGACTGCAGAACCGTAAAAAAGCGTAGCGAGAGAGAGAAAATAAGACAAATGCATCTAACTGAACTCCCCACCTTGCTGAGACTAGAGGAAGAGGAGGAAGAGCT
CAAAAGAATAAAAAAACAGGAAGAAAGGGAAAAAAGAGAAAGGGAAAACCAAAAGTGGCCTCCAGATAGATGGTGCGAATTGGAAATAAACCGATATAATAAACAATATAGAAAGGGCGACCTAACCAGGCAGGAAGTTATAG
AAAAATTCAGAGGGCAACCATTAAATGTACAACGAATAATCCTACCCGACTATGAAGGGGACTAAAATTAAATCAAAACAAACCAGGACAGGAGCAGAAGGCAGAAATAATCAAAACTGGCGGAAGGGGTGGCAAAAT
ATTGAAAAGAGAGGAAAGAAATATAAAAAGGCTAAAGGCTAAGTTACAGGTTACATAAAAAGGGAAATCTGCTTATAGATATTATGGTAAAATTAACTTAACTAAACACCTACTGGTTAACAAAATAATTATG
CCTGCATGGCACAAGCTGCGTACTCAAATCATTTCTCCTGACGCTATTGAAAATCCATCTTTACTTTCCAACCGAGGGACTTGCGACTGCGGTCTTTCCGCCTTATTGGCTCCTTATGGATCCATCTGCTGCC
GTATTGGGCGACACACCAGCGCTCCAACCTAAAAGAGAGATAACATGTTTTAATTCACTTTCCTTTTCTTATAACTAAATCACAACAACAGCAACAGCGCATCGGGCGACTGACAAAAGCATTAGCTCACCAA
GTCAGCAACAACAGCAGCAGCAAGACCAGAATCAGTTGAGGAGGAGGCCTGGTGGTGTGCTGAACATTTCGCCGCCCACACCCACCATTTCTGTAGGCCTGTATGACCCTGAGCCCAACGCCGGCATGGCAAA
TCCGGTCTTCCTGAGGAGGCGGGGCTCTAGTGTTAGGCGCGCTTTTGCCCCAAAAGCAACAACGACAGCGGCAGTAGGGTCGGCGCCCCCTGCGTGACCGAGTCCATCTTAGCAATCGGTCCTTTTGGCGGTG
TTATGCCGACGCGGCGGTCGCGCTTATGAGGACTGCCTGCAATGCTTGGCCATGAGACGGCGTCATCGACAACATTTCAATCACGCTCAGCTGGTTGACCGGGGGCAACATGCTTGCCATGCAGCAACAATGG
TGCATGCAGTCCAGCATGCAGCAACAACAACAATATTGGCAGCAGCAGACTAATGTTTCGCAGCAGATTCCATCTGGCAACTTCCGACGACCAGACTCCTCGCTTGGCCCCTGGAGACTCGCTCTGGAAAAAA
AAAGAAAGAAGCATGGATTAGTTTTTTTTTTCCCTTTAGCTATTGGATTAAATTTCTTTCCTGGACAACCGGCGCCTTCATGCAGATGACGCTGGACTGCGGCGATTCTTTCCCTCCAGCCACAGATGGGAA
GCTCACATCATGGCATATATGCGGCATCGTGCTAATCAGCTCCCCATGCTGAGCGAGATCTTGATGCGGCGATGCACTCACTAGCGAGCAGCGGGAACAGCATACAGCGTCAGCAACAGCAGGAAGAGACTTT
GTAGCAGAAGCCTTTCGGCATTCTCCGACGACCCGACTCCTCGATCGGGGTCAGGCAAATTCTCCTGGACCGACAAATTTTCCCGGCGTTTCGTCCTGGAATAAGGAAGGAAGCACGGATTAATTTCTCCCCT
TCAGCTTACGACCCTTCTCACCAACAGCAGCAACTACAACAACATCGGCAGCAGACGACTTGCAGCTGCTTGGCTCACCTAATGCGACAGCAATAATCCCGGCAAGGCCAGAAACGGCACCGAGCAGAAGCAC
GACAGGGTGAAGAACAACATGCCGCCCACGTCCACCAATCCGGAAGCCCTGCATTAGGGGTCTGAAGCGCCATTAACTGGAGGATCCGGCAGCGTATGCCTACGCAGAAGCGGGGCGTCGTAAATCCGGTGGC
CGGAATAGCGCCTTGAACAGCATGCTGTGTATGTTTGCCCCAAAAGCAGAAACTGCCGCAGCAAAGGAACCGCAAGCAGGATCATCACTCGTGGCAAACTGGCGTATTCCTGTTGAAGACGCGGGACTGCGGC
GCATTCTTCCTTTCCAACCGGGGGCTTTAGCGCTGCGGTATTTCTGCTTTCACGGCTCCATTTGCTGCTGCATCTGACGCCTCATCAGCGCTCCTCCCTGAAAGAAAGAGAATATGTATTAGTTTTCTTTCCA
TTCCTTACAATCTCTCTCACCAAACCTCCACGACACAACAACAGCAGCAGAAACAGGTCTAGCCCACAATGGCAGCAGCTACAGCAATCAGCAGAAGCAAGGCCGGCAAAAGCAACAACAAAAGCCCTCTCTG
AAAGCCCGGATGTGGAGACTGAAGCGCCGGAAACTTGAGGATCCGCCCCCGATGCACGCCCACCAACAATAAGCTGGAGGAGAAGTTACTGAAGCATGGGAAGCAGCACCAGGGGTCGCATACGCAGCGTCAG
GGCTTTGCGAACCAGATGAATGAGGAGAGACTGGAACAGCATAACATCAGCGGCAGCAACAACAGCGCCACGAGGGTCAGCGGCGTCTGCATGGCTGTGTCCAAAACAACTCCAATTCAACTGCTGTATGATG
CAGCGCACCAGCGGCTTTCACAGGGACCGTCTGAAACGCTTGGCAGGAGCCGGCTGAAGGCCTGGGCGAAGACACTGGAGACACCAATGCGGCTCCTTTCGGCCGACGGCAACAGGCTTGCCATGCAATAAGA
ACAGTAAATCCTGAAATAACAGCAGAAGATACAGACGTCCAAATTGCGAACACTGACGCCAGCCCTCACCTGAACGACTCACTGTGCAGCAGCAGCAACATCAGCACCGATGCCCTCAGCATCGGCAGCAGAG
GGTGTTTTTGGCCTTTGACAATTCCCTGCGACTTGACTTCTTCTTGGCATCTGGTCAATCATCACGTGCCAGCAAACAGTGGCTTGTCACCCTGGAAGAAAGAAGACTGGATTAGCTTCTCCCCTTCTAATAC
ATTTTGTGTGTTGAAATGCATCAGGCGGCGACACACTCACCAGCAACAGCAGCAACAACACCAGCGGCACCGGTAGCAGGAAATGGATCCTCAGCAAAAACCATCGGCACTTTCAGACGTCCCGTACTCCTCG
CCTGGCCCTGGCCGGTAACAGGGGCTTGTTAGATGGAGAAGACGACGGTCATCCGACGGACAGCAGCCTGAAGATGGAAGCAGGCCTACGCTGCCCACCTCTCCGATGCCTGCAGCAGCAACGGCAGCGGCTC
ATAAATGCAAACTGGCGCCAGCCCTCGGCTCTTCGGGCTCATGTAGGCGGTGACACACTCACTAGCAGCTAAAACAGCAACGGCGGAATTAGTAGAAGCAATGTTTTGCCGCAGCTGCCAGATGGCATACTCC
TAGCTTGGCCCCTGCCCAAACCGTATTGGACCGGCAAATGATTCGACAATCCGATCTGGAAAAAATAAGTATTGATTAATTTTTCCCCCACAGATTGGTATAAAATTGCATTTACGGCCAACCGGCTTCTTCC
TGTTGATGACGTCACGCTGCGCCGCATGCTTTTCGCCAGCCACAGTTGGAATGCATGCATTGGGGTTGACGGTCCGGATGGGCCGTTGCCTGTTATATGCTGGCATCTCCGCAGCGGCGTTGTGAGCAACGAC
ACTGAAAGCAACAATAATATTGTTAATTACTGCTCAAAACTATAACACATTTAATCACTTACACAATTTACACACGCTTCCTGCCTAGGAGTTGTGGGACCAGCTACCGATAATGCCAACAATACCGGGAAAC
GCGTCGCGGGACATCGATAACAATTTGGCGGTTAAAATGCTAGAACATCGAGTACCAGACATGTTTTTCCTGCCGATATATCGAAGCCCAATAGCCGCCAACGCTCAAAACATAAATCGGAGTGTGAGACCAG
ATACTGAAGCCGTTAAAAGCTTTTACCGGCATCGGTAGCTGGTCTCACACTCCGATTTAAATTTGGAGCGTTGGCGGGCTGTTTGACGCGAAGCTTCGCGGGATATCGATAACAATCAGGTGGTTAAAAATAC
TATAACATCGAGTACCAGCCGTGTTTTTCCTGTCGATTTATCGAAGTCCAACAGCCCGCCTTCGCTCCGAACACAAATCGGCAAGTCAGTAGCTTTTTAACCCGGCAGTGGCGCCTGTGCAGCAGCTATTTA
AAGTGAAAAACACGAAACGCAACAACAAAATGGACGGCCATAATGGAGACATAAATGAAGGATGGGCAACAGTACTATCTATCTCGTCGGATGATAGTAACCAACTTTCGTCGCCGCCGTCAATTATAGTCTC
ATCGCTGGACACCACGCCAACGCCTGTCTAATGCAAATCAACTTACATATTACTCTAAAATCTACTTATAACTGTCCCCTCTAATGATAAGCAATTTTTATGTTTACTTTAAACTTAAACCGATACCTTAAAC
TATGGCGCGCAAAATGACTACCAAGAAATCTAAAACTGTAAATTGTTGCAAACTATTTCTCCTAGTAATGTTAAATTTAAATGCGCGCATAACCGCAGCCAAGTTAACCAAAAATGTCACTCGTAAATGTAAT
TCCTAAATTAACACTTGTAACTAAGCGCGTATAAATACCGCAAATCCAAAAATGTAAATATGCCATAAATTGTTACTATATATTTTTAAAAAAGAAAAATCAGAGTACAATAAAAATGCCAGCGTCTATTAGG
CGTTGAAAATTTAAAAAAAAAAAAAAAAAAAAAAA

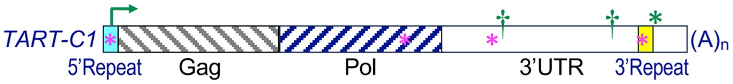

Fig. S18. Identification of potential E2F1- and Sd/dTEAD-binding sites in *TART-C1*. The nucleotide sequence of *Drosophila melanogaster TART-C1* (GenBank: AY600955.1), which includes the Gag protein and Pol protein genes, is annotated to highlight key structural and regulatory features. Key annotations: 5' Repeat is marked in light blue; 3' Repeat is marked in yellow. Potential E2F1-binding sites are located in: the 5' Repeat, the 3' Repeat, within the sequence encoding Pol/ORF2, and the 3'UTR. Potential Sd/dTEAD-binding sites are identified in the 3'UTR. Potential dE2F1-binding motifs (TT[C/G][C/G]CGC) are specifically highlighted in magenta.

Fig. S19.

Drosophila melanogaster **Telomere-Associated and HeT-A-Related Element (TAHRE) from the distal region of the XL telomere**

GenBank: AJ542581.2

5'UTR: 81..532
Gag: 533..3430
Pol: 3466..6777
3'UTR: 6778..10543

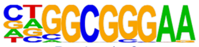

P-value:1e-3

E2F1(E2F)/Hela-E2F1-ChIP-Seq(GSE22478)

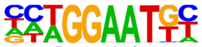

P-value:1e-9

TEAD(TEA)/Fibroblast-PU.1-ChIP-Seq

TTCCCGC or TTCGCGC or TTGGCGC

>AJ542581.2 *Drosophila melanogaster* Telomere-Associated and HeT-A-Related Element (TAHRE) from the distal region of the XL telomere

GTAATCAAAGGCAAAATAAATTGTGGATGCGGAACAGAATTTTATTCTGTCTCCGTACCTCCACCAGCAAAGTTAAAAAATTAAAAGCAAAGTTTTTTAAAAGTTCAAAAATATAATAAAATAAAGCAAATTAAATTTAATTA
ATAAAACAATTAATTTTATTTAATAAAATTAAACGCGCTTCGTCGGCAAATAACTCTCACGCGCAAATTTTATTAAATTCGCCTTTCAAGTTGAAAAATTAAAAGTTAAAATCGTCTTCCGGCCGCAAAGTTTGAACCGCGAC
GATACAAACATTTAATAGACAAACAAAAAGCGAACAATAAATCAGTGAATTATTTGTGCAAGCTGCCGCCATAACCAAAAGGAGAAGAAGCCAAAAGACGAAGAGGAGAAAGTAAACCAGGAGAAATTAAGAAGACAAGGAGG
AAAACTTATTTAAAAAGACGACCTAAACTGGAGGACAAATTACAAATTAAAAGCCAGGGTATTTATACCTTACAAGTATCGACCTAATATAATATAAATAAATATGTCCACGTCCGACCACCTATTTTCTGACGATGAGGTTCACTC
AATCTCCTCAAGCCCAGAACAGCGAAATTCACCATTCCACCTTGAAATATCGCCCATGTCCCATGAATCTGACAATTCTCAGTCTAATATAAGCATTATTAATCTGAGGAAATTGCCCTTAAAACCAACAAATAATATTTCAAAATGCTCTTCTGGGAC
TGCCATAAATATTTTTCATTCCCTTTCACACAAGGAGAAAGAGAACATGAACACCAATATTGCCCAAAAAGACCCCCTCTCGATCGACAATACAGCTGCAGACACGGACGGCGCCAAAAGTAGCATCTTGAAGGGGAAATTGCCTTCCCCTCCGCTCTC
ATCACACACATACAAGGGGAAACTACCTCCAGCAACGACCCACACTAACATATCTGCTCTTACTCACACTGACGCTTCTCAAAGAGAAAAAATACCCACCTCAGTGATCTGCACTAATGCAGCTGCAGCTACTAATACAAATGCAGATCTAGGCGCCAA
AACGAGCGACGCCTTGGGAAATTTCCCCTCCCTCTCACACAGCGATCATAGCATGGAGAACAACCTAAGTTCCTCCACCAAAATTGGACCCAATACAAATTCCCCTTCTTCTCACATACTCACCAACACAAGCCAGGCCACAAACATAAGCGCAGAAAG
CCGCTCAAAATTTCCCGCGCCCACCAATACTGACGCACGCCTCAAGAAGGCCATTACTAGTGACAAAGGGGAAATTCACACACAAATTCAAACAAACAAAAGCAAGGAACACAACCAAGAAAATAAACCATTTAATTACTTAAGTTGCTACGCTTCATG
GTCAACTTCAAACCCAAAGCCAGACATTTCCAAACTAAGTTTAACTAGGAAATCCACCAACCGTACTGGAAATAGTGGGAAAAGAAGCATTTCCCCCCACCAAAAGAATGCTTCGTTATGCCCTTCTGCTCAGGGTAATTTAAATTCAAATTTAAATTC
AAATTCAAATCCCAAATCTAGCGCCACTCCCACTGAGGTGAATTTATCAGCAGCCCGCACCCTCAGCCGGCCGGCTGCCAAGCGCGATTTATTTAATTCATCTTCCAGGAGCCCAGAAGAGCAGCCTATGAGTTTTTCGGAAGTGGTTGCTGGAACAGG
TCCAGATATAATAGCACCCTCCGCTCCTGCACCACTAACGAAAACTCCGGGCAAACGAACAAACAGCGATCTGGACTGCTCTAGCTTTAAGACACCTAATAAAAGATTACGTGCGACTCCTAACTTTGAAACTCCAAGCCTTTTCCCCCCGCTCATTAC
ACCCGTTTTTAAAAGTAAGGCGGCTCAATCTGTTTATGAGGAGTCCAAGGCCAGGAATGGACCCCCCCGCCAGCCGTTACCCTGCAGCAACAATGCTTCTGCTCGCAGCGCAACAGCACCACCCGGGATTGCCCCCCTACCCCCTCAGAACACAGATGT
AGAGCTGCCCCCCTGGAAAATCGTTCCCCAGAGCCGTAGAGCACCCCCTATACTAGTCAACAATGTGAGGGAAATTGTCCCACTGCTGGAAAAGCTGAATTATACAGCAGGAGTCTCCAGCTACTCCACCAGGGCAACAGAAGGAAACGGGGTCAGGAT
CCAGGCCAAGGATATGACTGCCTACAACAAAATCAAAGAAGTCCTGACCGCCAACGGTTTTCCTTTATTCACTAACCAGCCCAAATCCGAGAGGGGCTTCCGAGTCATCATCAGACACCTCCATCATTCCACACCATGCTCGTGGATAGTCGAGGAGCT
GCTGAAGCTCGGATTCCAAGCACGCTTCGCTAGAAACATGACGAATCCAGCTACAGGTGGCCCCATGCGAATGTTTGAAGTGGAGATAGTCATGGCCAAGGACAGCAGCCATGGCAAAATTATCTCATTGAAACAACTCGGTGGGCAAAGGGTGGATAT
CGAAAGGAAAAACAGGACTCGGGAGCCGGTCCAGTGCTACAGATGCCAGGGCTTCAGGCACTCCAAAAATTCATGCATGAGGCCGCCCAGATGCATGAAATGCGCTGGCGGTCACCTGTCATCCTGTTGCACAAAGCCAAGAACCACCCCTGCCACCTG
CGTCAACTGCTCTGGTGAGCATATTAGTGCGTACAAGGGATGCCCCGCTTATAAGACTGAAAAACGAAAACTGGCGGTCAACAACATTGACATCAATAAAATAAGGACAATCAAGGACGCGAACATTACCAACTATGGACGACAGGGCCCTCCCTCTCG
CAACAACTTTCCCCGGCTACCATTTAGCTCCTCAACCTCCAACAGGACAACAGCCGAATCCCGCCAAGACACAGCAAGAGCACGGCGGAATAACCCCTTCCGGCAAAACAGAAACGAAGCAAGGCCAATCCAGCCACGCTTCTCCTCCCACGACTTTGC
CATCCAGAAACGTCTGAATAAATGGCGCCGCAACTCTGACAACGTCTCCAAAAAAGGCACGATAAATCCCAAGGACAAGCCAAAGCCACGAACACCTAACATGACGAGCAATCCTGCACAAAAACACCTGGAAATGTTCCAGGAAAAGCTCCGCAAAGC
AAGATGTGAACGCAAGGAACAGGACCCGGAAGAAAAGAAGACAAACATCAGGATGGGAGACGATGAAAGCCCGCCAACCACCAGCAGAGCTGCCAGAGCATTCCTCAAGCCAAGGATCATAGACGACAACATCCCCACGCCTATGGATACATACTCTAA
CCCGCAAAAGAGTCCCTCTGACTTCGACAGCAAAAGCCTTACACAACGAGTGGAAAATATTGAAAAGAAAATTGATAATCTAATGGAACTATTATTCAAGTGCCTCGAATCCACGAAAGAGTCTACCTTAGCACACCTCATGACCTCCTAAATCACTAA
ATTCTCTACGCAAATTAAAATACTACTATGACCTTTAATCTAAACAACGGACAAGCTTCCAATACCCTAAAAATAGGGTACTGGAACTCATGTGGAATTACAAATAAAACAAATGAACTGGAGGCCTACATCAAAAAAGAGGGTATTCAAATTATGTTA
GTAACTGAAACCAGGTTAGAGCGAAATTCCAACGCACTTAATATCAAAGGCTTCCACACCTACCTAGCACAAAACCCTACATCCCACAGAAAAGGCGGCACGGCCACCATAGTCAGTCAAAACATTAGACATGCCTGCCTAAATCCCATAGAGACTGAC
TTCATGCAGAGCGCACCCATCGCACTTATCCCCTCAAGTCGAATACGCGCAGACATGACAATTGTTGCCCCAATATACTGTCCGCCTGTATATAAATGGACCACTGAACAATTCTCGAAGCTCTTCAACCACTTCGAGATGCTTCTCGATGGTAAGTCC
AAATTTATACTTGGCGGCGATTGGAATTGCAAACACAGACTCTGGGGTAACTATTTATCCTGCGCTAGGGGAAGATCTCTGTCACAGTCAATCCTTGCAAGGAAAGACCTTGACATAGTTGCTACCGGCCATGCCACACACTTTCCCTTCGACAAGAAA
AAACAGCCCTCTGCACTGGACTTTGCAATATGCAAGGGCTTCCACACTCAAAAACTAAAAACCTACTCCACAGATGAACTCAGCTCTGACCACCTTCCCATCCAAATAGTCCTAGATCCTGACGACTCAGACTGGCAACATAATAAGACAAACAATGCT
ATTATACAAAAAAGAACAGATCTCACTAAATTTAAGAAAAACCTAGAAAACAAAATATTACTAAACACCGAAATTCGCACAGGACAGGACATCGACGACTGCATAGACATTCTTATCAATAACATAAAATCCGCAGCCAATGAAGCCACCCCACCTAAT
CGCCCCCAAAATAACCGCACCTACAATTCTTCAAGAAGATCCACAAACCTAAGGCTAGATGAAGCAACTAAAAGGTTACTTGAGGAGAAAAAGGAGCTAAATCGAATCTTTAGAGCAGTAGGAACTGACGAAGCCCGAAGATGGTTTAAGAACGTGCAA
AATAGACTGTCTAAAGAAATCAGAAAGCTCAAACAAAAACTACTCAACCGCAACCTACAAGATATTGACACTACGGACCGATATAGAACGCAAAAACTATGGAAAACCACAAATACAATTAAAATGCAACCTCGACCCTGCTGGCCGATAAAAAAGGAC
AATGACGACAGCCGTCAAAGAACTGACTACCCCTGGACCAGGACTTTAGATGAAAAAGCTGAAGCCTTTGCATCTCACCTTGAGTCCCGCTTCAGGACAAACCAAATTAACGACGCCAAAGACAGGGAATTCGTCAGAAATGAGCTAAACAAATTTAAA
TCATTAAATGCGAGCGGCGAATCCGGAAACAGCAACTTCAAACCAGTCACTCTGGCTGAACTAAATGGCCTGATAAACTCACTGGAATTAAAGAAAGCCCCAGGAACTGACAATCTTAACAACAAGACCATAATAAACTTACCTACAAAGGCCAGAATA
TATTTAATACTTATTTATAACAACATCCTGAGAACTGGACATTTCCCGAACAAATGGAAGCACGCTAGCATCTCAATGATTCCCAAACCAGGGAAATCACCATTTGCTCTAAATTCATACCGCCCAATCAGCTTACTCTCTGGTCTTTCCAAACTACTC
GAAAGAATACTACTGAAACGACTGTATGACATTGACTCTTTTGCCAAAGCAATCCCTTCCCATCAATTTGGTTTCAGAAAGGATCATGGAGCGGAACATCAGCTGGCCAGGGTGACCCAATTTATTCTAAAAGCTTTTGATGAAAAAGATGTCTGTTCT
GCCACATTCCTTGACATTACGGAAGCCTTTGACCGAGTATGGCACGACGGCTTGCTATATAAACTATCCAGACTCATCCCCAGATACCTATTCGACCTACTTGAAAACTATTTATCTAATAGAACCTTCTCAGTAAGGATCGACGGTGAAACAACGTCT
AGGATAGGTAATATTAGAGCAGGAGTGCCCCAGGGCAGCATACTGGGACCGGTCCTCTACTCAATATACTCATCCGACATGCCCTATCCCATCGTAAAAGACTATATGCGTAACATATCCTTCCCTGATTACCACCCAACTAATATTATCTTAGCTACA
TATGCAGATGATACCATAATTCTTAGCCGGTCCAAATATACCAAGCTTGCGATCAACCTAAATCAAAACTACCTTAACGTCTTCTGTAGGTGGTCAAAAAAATGGGACATAGCAATTAATGCAAAAAAAACCGGACACATTCTTTTCTCCCTAAAAAAA
GAACAAACTAATATATACACTCCCCCACTAATCAACGGACAAAGAGCTGCCAAACTAAACAAACAACGCTATCTCGGACTTATGCTAGACAGAAGACTGACCTTTTGTGCACACATGACGCTGCTAAAGGGAAAGACTATAGCTGCATATAAAAAACTG
GAATGGCTAATAGGAAAAAACAGCCACCTACCCAAAAATGCAAAAATTCTCCTCTGGAAGCAAATTGTCTCCCCCATCTGGCATTACGCCATAGCAATCTGGGGCTCGCTGGTATCTGACACCCAAGCAAAGAAAATTCAAACAATGGAAAACAAATAC
ATCAGACGAATCATAAACGCCAGCAGATACACGAGACAAGCAGACATAAGGACAAAATATAACATTAAATCATTTGATGAAATTTTTGACAAAGCAAGCCAACGCTACGCCAACTCCCTCACTGACCATGAAAACCCTTTAATATATGACCTCCTTATC
AACGCCTACAAGCCGAACAGACTGGAACTAAGCAAAAACAGATACGTCAAGCAATTATCAAAATATATACTGCCCCTTCAACAACACCGACCTAAACCACCCGCAGAACCCATCTACAGCTCCATACATAATTATACAAAAAAGGAAGAAGCTGAAATA
GTCGCCAAAATGAGAACCAAATTCAGACAGACCCTTCCAACCCTCCTCCGGATCGCTGACCAAGAATTGGAAATAAGACAATCCATAGCTGAGGAAAAGAGAAAGGAAAAAGAAAAATCCGAAAGGAGAAAAGCCTTAGAGAAGGGACCGCCAGATAGA
TGGTGTGAACTTCAAATAAACAAATACAGCAAATTATACAGAAAAGGACTAAGGGCCAGAGAGGAAATCCTTGAATTAATGCTCGGACAACCCGCCACTGTAATCAAAATAGTAATCCCAGACTATGAACCCGAAGACGACCTAACAAAAAAGTCTTGA
AAACTAAATAAAAGCAAATAAAACCAAAAGTACATGTATTTACAATAATCATTGATTGTATAATTGGTGATTATAATATAACAATTATAATTATTAATATAATTGATTGTCATAA
TTGTTAGCTATTGATTATAAATAACTAATCAAAATACAAACTACAAACTATGACCGACGGAAAGACGCACGCCGACCTGCTTCTCTTCCTACAATAGCGATACACATCTCCTTCT
CCATAGTCAGCATCTTTCTGTGGAAAAACAAACCAATTAGATGGATGATACAAAAACACAAATAATAACCACACCTCAACGCATCCGGATAAAAACAACCAACGACAACGCATTC
CAGCTGATCATGACGAAGTGATGCGAATAAAATCACCACCTGGACATAAAAGAAGAATCGGTAGATGGATATGAAAAGGATTGGTGCGGCGAAAGCATGATGAATATAAGGCGAC
TCGCTGCAGCAATATATGCACAACGTCACTTACCTGAATCTTCTTGCCGCACAGTCTTTTGAAGATCCTTATCACCGCTGCAATCCACACACATCGCCGCATTGCTAAAGACAGG
CCATCTAAGCTGACCCAGCGCCGATTAGGACACTCTGTTCGACGAGCGCCTACAGCGAATTGCCGCTAAAACCTAAAAACAAAAAATTTATTAACAAATGAAATACAAATATACA
AAATTCAAATAAAACAAAGCACTTACCTCACTGACCGCATCCAACTGCACGCAACACATCCATATCCAACATAACAGACAAGAGGAGACGGGCCCTCAAACGTAAAACAATATCG
CCAACTTTGCGCTTACAAACACAAAAAAATTTACAATTTTATGATGCCGTCTCCTCTTCCCGATGCCACTGCCTCAATATGGATTACTAGCGCGGAGCCGACAGCACACTAAAAG
GCTGAAAAATTTGTCCTCAAAATTAATATTTTTCCTTAGTACCACTATCCCAACGAATTTTCCGCAAACCTGAAATAAAAAGAAAATTAATAAGAAAGTGATACAAAATTAACTA
AAAACAAATAGAAAATAGCAAACCGGACAAGCAAAGTAACAGATATAAATATGCTACTTCATCCTGCTGAAGACACGCATCCATGCATCCTTCTTCCAAGACTGCAAAACAGAAA
GAAGGAAACACAAGCTATACTGGAAAAATATATTAAATCACAATACTTATCTAATTGCCAATTTGAAGAATCCTAAACCACGGCATCCCAGCGGCGATGGCCTTATCTTTAGCT
GCTACAAATGTACCTGAAAACAAAAATCAAAAAACAAAAAGTAATTCAACTATAAAAACAAACATAATACTTACCTCCAGACTATTTTCCTCCCGAAAAACATACCTGGAAAACA
AAAACAATGCAACTATATAAACAAATAAATACACAAATAATACTTACCTCCGAACTGCATTCCACCTAATGTACCTGAAAAAATACAAAAATTACAGAAATCACAAAAATAAATA
ACAAATGCTATACTTACCTAATTTTAATATTACACCCATTCCCATGGCCCAATCTTTGGGCGGTCCCCAGCAACAATTCCTGACCCGGAACATTCTAAAATAATAGGAAAATAAA
TAAGATTGCGACTCAAAATTAAGCAATAACACAAAAAAAAAAACAACAAACCTGGCAGACAAATTAGCTGACGATAAATTACAACACCATCCTGCTGACGTCACGCACGAAAATT
CTCTCTCCTAAAACACGGGATCCCAGCGGCGATGTCCTTAACTTTAGCTGCTACAAATGTACCTGAAAACAAAAAAACAAAAATTAATTCAATTATAAAAACAAACATAATACTT
ACCTCCAGACTATTTTTCCTCCCGAAAAACATACCTGGAAAACAAAAACAAGGCAACTATATAAACAAATAAATACATATATAATACTTACATCCAAACTACATTCCACCTAATG
TATCTGAGAAATACAAAAATTAGAGAAATCACAAAAATAAATAACAAAGGTTATACTTACCTAAAATTTAATATTACATCCATTCCCATGGCCCAATCGTTGCGGCGGTCCTCAG
CAACAATTCCTGACCCGGAACATCCTAAAATAAAAGGCAAATAAAATAACACACATAAAATAGCAAACCTGACAGGCAAACTAGCAGATGCTAATTTGCAACACCATCCCGCTGA
CGTCACGCACGAAAATCCTTTCTCCAAGAGCGCAAAAACTGAAAAAAGAAACACAAGCTAACACTGGGAAATATATACTTCTTAACAAAATACTTATCTAAATTGCCAACCGGAC
GACTTCAAAGCTGCGGCCTAATTTTAATATTACATCTATCTCCGTGGCCCAATCTTTGCGGCGGTCCTCAGCAACAACTCCTGACCCGGAACATCCTAAAATAAAAAGAAAAACA
AATAAGAATGCGACTCAAAATTAAGAAATAACACACAAAAAATAACAAACCTGACAGGCAAACTAGCAGATGCTAATTTGCAACACCATCATGCTGACGTCACGCACGAAAATCC
TTTCTCCTAAGACCGCAAAAGCTGAAAAAGGAAACACAAGCTAATACTGGGAAATATATATTTTTAACAAAATACTTATCTAAACTGCCAAATTGACGACTTCAAAGCTGCGGCA
TCATAACTACAACGACCTCCAAAATAACTTTCAAAAAATGTACCTGAAAAACAGAAACTAATGAAATCATAAAAACAAATATAATACTTACCCCCAGATTAGCTATTACCTGAAA
AACAAAAAATTAATGCAACTATATAAACAAATAAAAACAAATATACTTACCATCAAACTATCCATCACATTGTACCTGAAAACAAAAACAAATGCAATCATAAAAAACAATCCTA
ATGCAATACTTACCTATCTCTAACTTTACATTCATTTCCATGCCCGTATCGCTGCGGCGGACTTCTGCAACAAATCTTGAACCGGCGGCCCCAAGCTGCCAATCCCGACGCAATG
GCCAATAGACGAGGCGCTCCTGGCAACTCTTGGCGAACAACTAAGCTGCATTCTACTGACGACTCCTCTGCCACAACGAGACAGATTCCTCCATTACGATCCCAGCAGCTTTAAG
ACCTTGTAACGACGGCTGCGCTGGACCCTCCTTGTTCTACCTTCTTTTGATGACCGGCGAAGTGGCCCTGCAAATTAACAATTTATCAAACAACTGCAATCATCTGCCACTGAGG
TTGAAATATATACCTACTAAATGACAGCGGCGCGGGATACACTCACCTATAATAGGCTGCTTGCAGCGCTGGCCGGACATGCATATTGCAAGTGGCACGCATCCAGAGTCCACAA
CAAGCCCCAGCCAGAATGCCAAAATTACTCACCTGCAATGTTTCCTGAGGCCTCCAGCGACTCGGTGCTTCCGTCCTTCCGACGGGGGTACCTGAAAAGAATAAATCAATAACAA
TGTTAGTTTTAAATTTCAATGTTTTCTATAAAATCATTTCAATTGTAAATTGTAAACATACCATACAATATGATAATGTTACCTGTCCTAATTACTGTCAAAAGCCTAAGTCTAC
AAAATACTAACTACTTTTACATTATTACTAACTATGTCCAACCCCCAAACTCACCACATGTAATGTTACATTCAAAAATGCAAATTATTGTACCTATATATTACAAACTCTGTAA
TCAAAGGCAAAATAAATTGTGGATGCGGAACAGAATTTATTCTGTCTCCGTACCTCCACCAGCAAAGTTAAAAAAAAAAAAAAAAACCAGCAAAGTTAAAAAAATAATAAATAAAATAAAAT
TAAATAATTTAAACAATAATAACAATAATAAATAAATAATAAATTAAATAAATATTTAATAAAATAATCCGCGCTTTCCCTTGCTAAATATTCTGACGCGCAAATATAATTTAAATCGCCTTTTCTTTTTGACAAATTAAA
AGTTTAAATATTGTCTTCCGGCCGCAAAGTTAAAACCGCGACAATAAAAAAC

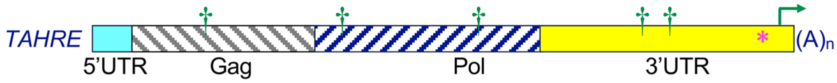

Fig. S19. Identification of potential E2F1- and Sd/dTEAD-binding sites in *TAHRE*. The nucleotide sequence of *Drosophila melanogaster* Telomere-Associated and HeT-A-Related Element (*TAHRE*) from the distal region of the XL telomere (GenBank: AJ542581.2) is annotated in the conventions of Fig. S14. A single potential E2F1-binding site is identified in the 3'UTR, while multiple potential Sd/dTEAD-binding sites are located within the coding region and the 3'UTR. Potential dE2F1-binding motifs (TT[C/G][C/G]CGC) are specifically highlighted in magenta.

Fig. S20.

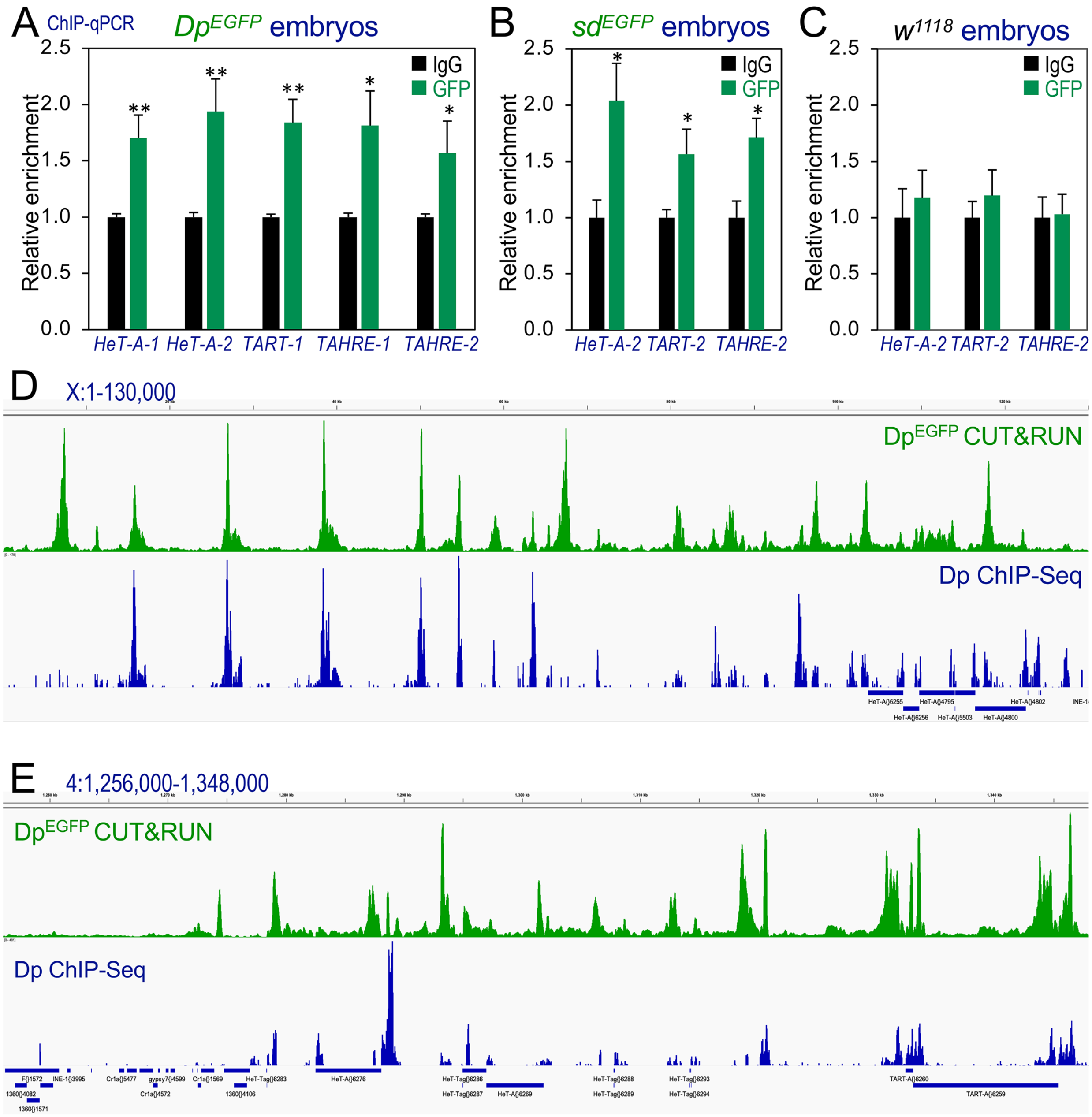

Fig. S20. Validation of CUT&RUN results for Sd and Dp binding to TR elements. (A-C) ChIP-qPCR analysis: Dp^{EGFP} (A) and Sd^{EGFP} (B) homozygous embryos were analyzed for binding to TR elements, using w^{1118} embryos as the control (C). ChIP-qPCR validated the binding of Dp and Sd binding to TR elements. (D, E) Comparison of CUT&RUN and ChIP-Seq data: Our CUT&RUN data for Dp^{EGFP} (green track) were compared with ChIP-Seq data from Zappia et al. (*80*) (GSE102043, dark blue track), which used a monoclonal anti-dDp antibody (#212) in pupal muscle tissue. Overlap of peaks: (D) X chromosome telomere (X:1-130,000): substantial overlap between Dp CUT&RUN peaks and Dp ChIP-Seq peaks. (E) 4th chromosome telomere (4:1,256,000-1,348,000): similar overlap was observed. Several TR elements are annotated and displayed below the tracks, confirming that both datasets identify consistent binding sites for Dp at telomeric regions.

Table S1. List of the *Drosophila* strains used in this study.

Stock #		Genotypes	Comments
BDSC	7011	*w[1118]; P{w[+mC]=Cg-GAL4.A}2*	*dCg-Gal4*
BDSC	6870	*w[1118]; P{w[+mC]=Sgs3-GAL4.PD}TP1*	*Sgs3-Gal4*
BDSC	99568	*y[1] w[*]; P{w[+m*]=GAL4}en[GAL4-33]*	*en-Gal4*
BDSC	30515	*y[1] v[1]; P{y[+t7.7] v[+t1.8]=TRiP.JF02519}attP2*	*Dp-RNAi*
BDSC	67388	*y[1] w[*]; PBac{y[+mDint2] w[+mC]=Dp-GFP.FPTB}VK00033*	*Dp-GFP*
BDSC	4770	*w[1118]; P{w[+mC]=UAS-E2f1.N}3B P{w[+mC]=UAS-Dp.D}1-4b/TM6B, Tb[1]*	*UAS-e2f1 UAS-Dp*
BDSC	56240	*y[1] w[*]; Mi{y[+mDint2]=MIC}MED7[MI10755]/TM3, Sb[1] Ser[1]*	*dMed7[MI10755*
BDSC	34663	*y[1] sc[*] v[1] sev[21]; P{y[+t7.7] v[+t1.8]=TRiP.HMS01140}attP2*	*dMed7-RNAi*
BDSC	34575	*y[1] sc[*] v[1] sev[21]; P{y[+t7.7] v[+t1.8]=TRiP.HMS01049}attP2*	*dMed14-RNAi*
BDSC	34664	*y[1] sc[*] v[1] sev[21]; P{y[+t7.7] v[+t1.8]=TRiP.HMS01141}attP2*	*dMed17-RNAi*
BDSC	34574	*y[1] sc[*] v[1] sev[21]; P{y[+t7.7] v[+t1.8]=TRiP.HMS01048}attP2*	*dMed31-RNAi*
BDSC	36744	*y[1] sc[*] v[1] sev[21]; P{y[+t7.7] v[+t1.8]=TRiP.HMS03004}attP2/TM3, Sb[1]*	*Rbf1-RNAi*
BDSC	31965	*y[1] v[1]; P{y[+t7.7] v[+t1.8]=TRiP.JF03119}attP2*	*dYki-RNAi*
BDSC	1027	*sd[1]*	*sd[1]*
BDSC	29352	*y[1] v[1]; P{y[+t7.7] v[+t1.8]=TRiP.JF02514}attP2*	*sd-RNAi*
BDSC	79614	*y[1] w[*]; PBac{y[+mDint2] w[+mC]=sd-GFP.FPTB}VK00033*	*sd-GFP*
BDSC	6816	*y[1] w[1118]; p53[11-1B-1]*	*p53[11-1B-1]*
		UAS-dCdk8-RNAi CycC-RNAi	*Jianquan Ni*
		UAS-dCdk8-RNAi	*Jianquan Ni*
		UAS-dCycC-RNAi	*Jianquan Ni*
	TH00816	*UAS-dE2f1-RNAi*	*Jianquan Ni*
		w[1118]; +; +; 118E-15	Lori Walrath
		w; +; FRT80B dCdk8[K185]/TM3 Sb*	Henri-Marc Bourbon
		w; +; FRT82B dCycC[Y5]/TM3 Sb*	Henri-Marc Bourbon
		w; +; dCdk8[K185] CycC[Y5]/TM6B Tb*	This work
		w; +; dCdk8[ΔmCherry]*	This work
		w; +; dCdk8-EGFP*	This work

Table S2. Primers used in qPCR and ChIP-qPCR assays, and the generation of the *dCdk8*^{EGFP} strain using CRISPR-Cas9.

	Primer name	Primer Sequence (5' to 3')	References
qRT-PCR and qPCR	*HeT-A-5.2Q*	ATCCTTCACCGTCATCACCTTCCT	(*92*)
	HeT-A-3.2Q	GGTGCGTTTAGGTGAGTGTGTGTT	
	TART-5.1Q	AGAGAGGGAAAGAAGGGAAAGGGA	
	TART-3.1Q	ATTTCCTGCCTGGTTAGATCGCCA	
	TAHRE-5.1Q	TACCATAATTCTTAGCCGGTCCAAATATAC	
	TAHRE-3.1Q	CTCGTGTATCTGCTGGCGTTTATG	
	TAHRE-GAG-ORF-F	CTTCCCCTCCGCTCTCATC	(*47*)
	TAHRE-GAG-ORF-R	CCTAGATCTGCATTTGTATTAGTAGCTG	
	jockey-gag-RT-junc-F	ACGACTCAATCTAGGGCTCGTG	
	jockey-gag-RT-junc-R	CGTCCATTCTCGTATTGATGG	
	TAHRE-5.4Q	TTGTCTCCCCCATCTGGCATTAC	This work
	TAHRE-3.4Q	TGCTGGCGTTTATGATTCGTCTG	
	TAHRE-5.5Q	ACAACCAACGACAACGCATTCC	
	TAHRE-3.5Q	TCGCCGCACCAATCCTTTTC	
	RasGAP1-5.1Q	CATCTCGTCGTGTTTCTTTCTCCG	
	RasGAP1-3.1Q	GAAAAGTGTCTTCCTTCGCCGC	
	Rp49-5.1Q	ACAGGCCCAAGATCGTGAAGA	
	Rp49-3.1Q	CGCACTCTGTTGTCGATACCCT	
Generation of *dCdk8-EGFP* line	*dCDK8-gRNA-L-5.1*	gtcgTTAAGGCTGTGTTATCG	This work
	dCDK8-gRNA-L-3.1	aaaCGATAACACAGCCTTAA	
	dCDK8-gRNA-R-5.1	gtcgAATTTCTAGCCTTCTAA	
	dCDK8-gRNA-R-3.1	aaacTTAGAAGGCTAGAAATT	
	homolog-L-5.1	GGCCGCCATGGCCGCGGGATCACACGATATGTATTCGGCA	
	homolog-L-3.1	CGATAACGATAACACAGCCTTAAGATATCATGGAAAATGG	
	dCDK8+EGFP-5.1	AGGCTGTGTTATCGTTATCGGGAGACAGCTGATTGGCGGC	
	dCDK8+EGFP-3.1	TGTTGTAGAAAAAATGCTGCATGTATCAGTCTCTCACTTGTACAGCTCGTCCA	
	homolog-R-5.1	GCAGCATTTTTTCTACAACACTGCAGAATTTCTAGCCTTC	
	homolog-R-3.1	CAGGCGGCCGCACTAGTGATATTGTTCTGCATCCTGGCTA	
ChIP-qPCR	*Rp49-5.1C*	TACAGGCCCAAGATCGTGAA	This work
	Rp49-3.1C	TCTCCTTGCGCTTCTTGGA	
	Het-A-1-5.1C	CTCTCACTCACCAATACTACTGCAAG	
	Het-A-1-3.1C	CTAAAGTCTGTGTGTGTGTGAGTAAGT	
	Het-A-2-5.2C	CTATCCTAGTCAACGACGTGAAGG	
	Het-A-2-3.2C	GTTGAAGGCAGTCATATCCTTG	
	TART-1-5.1C	CGCAATTGTTCCTGCCAACCG	
	TART-1-3.1C	CTGTTGTTGGTCTCATGCTCC TAC	
	TART-2-5.2C	GCTATTATCTTTTTTTTTTGCCGCCA	(*95*)
	TART-2-3.2C	GATTTTTCTGCTTCGTTTCCGCTT	
	TAHRE-1-5.1C	CAACTGCAATCATCTGCCACTG	This work
	TAHRE-1-3.1C	GTTGTGGACTCTGGATGCGTG	
	TAHRE-2-5.2C	CGAACTGCATTCCACCTAATGTAC	
	TAHRE-2-3.2C	GGTCAGGAATTGTTGCTGGG	
UAS-dE2f1-RNAi line (TH00816) in the *pNP* vector	forward	ctagcagtATCCAACGATATCACAAATTAtagttatattcaagcataTAATTTGTGATATCGTTGGATgcg	This work
	reverse	aattcgcATCCAACGATATCACAAATTAtatgcttgaatataactaTAATTTGTGATATCGTTGGATactg	
